# Novel Sesquiterpene and Diterpene Aminoglycosides from the Deep-Sea-Sediment Fungus *Trichoderma* sp. SCSIOW21

**DOI:** 10.3390/md21010007

**Published:** 2022-12-22

**Authors:** Hongxu Li, Xinyi Liu, Zhangli Hu, Liyan Wang

**Affiliations:** 1 Shenzhen Key Laboratory of Marine Bioresource and Eco-Environmental Science, College of Life Sciences and Oceanography, Shenzhen University, Shenzhen 518060, China; 2 Key Laboratory of Optoelectronic Engineering, Shenzhen University, Shenzhen 518060, China

**Keywords:** Sesquiterpene aminoglycoside, diterpene aminoglycoside, *Trichoderma* sp., deep sea fungus, NO-production-inhibitory activity, anti-fungal activity

## Abstract

Six new sesquiterpene aminoglycosides, trichaspside F (**2**) and cyclonerosides A–E (**5**–**9**), two new diterpene aminoglycosides, harzianosides A and B (**10, 11**), and three known sesquiterpenes, trichodermoside (**1**), cycloneran-3,7,10,11-tetraol (**3**), and cyclonerodiol (**4**), have been isolated from the n-butanol extract of *Trichoderma* sp. SCSIOW21 (Hypocreaceae), a deep-sea-sediment-derived fungus. The structures and relative configurations of the new compounds were determined using spectroscopic techniques and comparisons with those reported in the literature. The absolute configurations of the aglycone part of cyclonerosides A–E (**5**–**9**) were tentatively proposed based on optical rotation and biogenic considerations. Cyclonerosides A–E (**5**–**9**) represent the first glycosides of cyclonelane-type sesquiterpenes generated from *Trichoderma*. The NO-production-inhibitory activities were evaluated using macrophage RAW264.7 cells. Among the isolated compounds, trichaspside F (**2**) and cyclonerosides B–E (**6**–**9**) exhibited the strongest NO-production-inhibitory activities with IC_50_ values of 54.8, 50.7, 57.1, 42.0, and 48.0 µM, respectively, compared to the IC_50_ value of 30.8 µM for the positive control (quercetin). When tested for anti-fungal activities against several pathogenic fungi, none of the compounds exhibited significant activities at a concentration of 100 µM.

## 1. Introduction

Filamentous fungi have larger genomes and more biosynthetic gene clusters (BGCs) compared to bacteria, which leads to greater chemical diversity, making them one of the most important resources for natural product drug discovery [1]. However, with the intense study carried out over the last century, the high rediscovery rate from terrestrial fungi has seriously hindered new drug development from this resource. Research on natural products from marine fungi has received a great amount of research attention in recent years. A total of 346 compounds have been characterized from marine-sediment-derived fungi during 2005–2015 [2,3], in which almost half of the compounds (157) are reported to exhibit anti-microbial and anti-cancer activities. Further, 246 compounds have been reported from marine-sediment-derived fungi during 2016–2020 [4]. Among them, 12 compounds exhibit antiviral activity, 57 compounds have anti-microbial activity, and 62 compounds display cytotoxicity. In addition, 70 compounds have cytoprotective activity, including anti-infection, anti-oxidant, and neuroprotective activities, which demonstrates the diversity of the research being conducted on compound activity. However, anti-microbial and anti-cancer activities are still of significant interest in marine fungi natural products. Approximately 200 more compounds exhibiting anti-microbial and anti-cancer activities were reported in 2019–2022 from marine fungi [1]. Considering the best-selling drug lovastatin as an example, which contains hypolipidemic activity and was first discovered from a *Penicillium* species fungal strain, more bioassays should be prepared to screen for natural products in marine fungi to discover new active compounds with potential applications.

The number of *Trichoderma* spp. fungi isolated from nature was limited to dozens just a few decades ago. However, the introduction of molecular evolutionary approaches has led to the rapid expansion of the *Trichoderma* taxonomy, resulting in the discovery of ~50 new species each year. By July 2020, 361 *Trichoderma* species had been successfully cultivated and their DNA barcoded [5]. *Trichoderma* species are considered to be treasure troves of natural products. By 2008, 186 compounds had been identified from *Trichoderma* [6], while 203 compounds were characterized between 2009 and 2020, including terpenoids, cyclopeptides, diketopiperazines, alkaloids, and polyketides [7]. Among filamentous fungi, *Trichoderma* species are some of the dominant producers of terpenoids. However, glycosylated sesquiterpenes and diterpenes have been rarely reported in the literature [8]. To the best of our knowledge, three trichothecene glycosides, namely trichodermarins L–N, have been discovered in marine algicolous *Trichoderma brevicompactum* A-DL-9-2 [9], while one bisabolane acetamido glycoside, trichodermoside, has been isolated from marine-derived *Trichoderma* sp. PT2 [10]. Three bisabolane acetamido glycosides, trichaspsides C–E, have been isolated from *Trichoderma asperellum* A-YMD-9-2, a marine algal endophytic fungus [11], and trichosordarin A, a diterpene glycoside, has been isolated from the marine-derived *Trichoderma harzianum* R5 fungus [12]. 

Anti-microbial, anti-microalgae, anti-cancer, and phytotoxic activities have been reported for sesquiterpenes and diterpenes isolated from *Trichoderma* species [7]. Cyclonerane sesquiterpenes also exhibit nematocidal activity [13]. However, the effects of sugar moieties on the activities of these compounds remain under debate. The sugar moiety in bisabolene-type sesquiterpenes has no effect on their growth-inhibition activities against marine phytoplankton species [11], and glycosylation of trichothecene sesquiterpenes appears to reduce their anti-fungal and anti-microalgae activities [9]. In contrast, the amino sugar moiety in bisabolane sesquiterpenes is indispensable for their activities against several aquatic pathogenic bacteria [14]. Trichodermoside, a bisabolane sesquiterpene glycoside, has been shown to weakly inhibit the growth of human HeLa cells [10].

**Figure 1:**
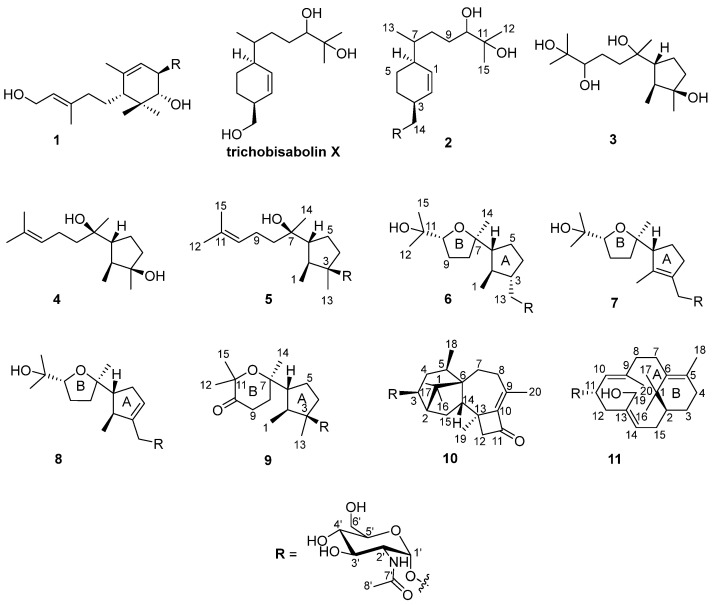
Structures of compounds **1**–**11**.

During our research on anti-inflammatory natural products obtained from deep marine fungi [15,16,17,18], we found that the n-butanol (n-BuOH) extract of the *Trichoderma* species SCSIOW21 inhibits nitric oxide (NO) production in RAW 267.4 cells stimulated by LPS. In the present study, we have characterized six new aminoglycoside sesquiterpenes, trichaspside F (**2**) and cyclonerosides A–E (**5**–**9**), two new aminoglycoside diterpenes, namely harzianosides A and B (**10**, **11**), and three known sesquiterpenes, trichodermoside (**1**), cycloneran-3,7,10,11-tetraol (**3**), and cyclonerodiol (**4**), from the fungal culture (Figure 1). The NO-production-inhibitory activities and anti-fungal activities of these compounds were studied.

## 2. Results and Discussion

The fungal strain was statically cultivated in rice broth containing 3% sea salt and then extracted with n-BuOH. The extract was subjected to silica gel column chromatography, followed by HPLC on an ODS C18 column to obtain 11 compounds (Figure 1).

**Figure 2:**
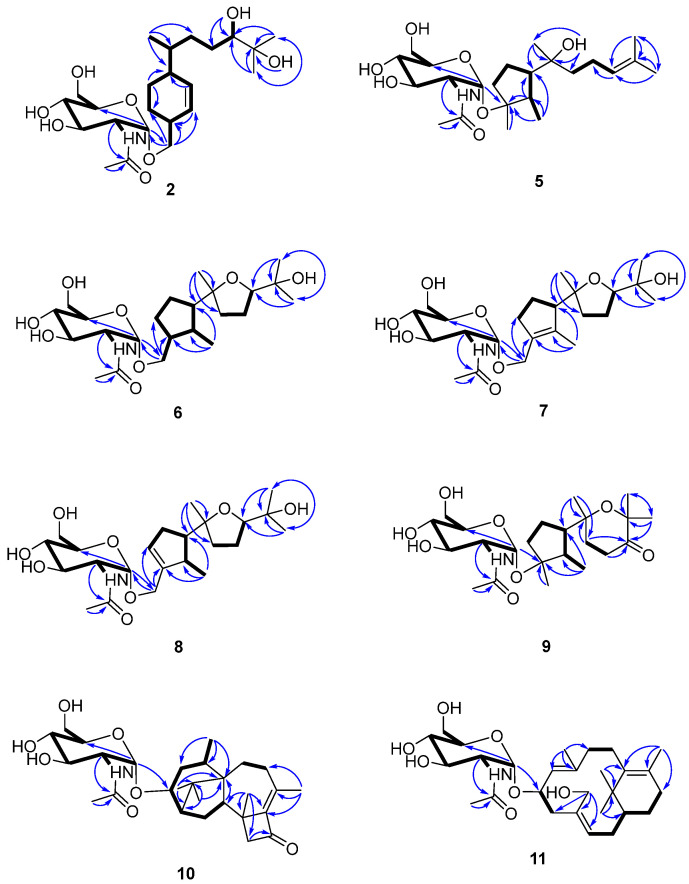
Key 2D NMR spectroscopic correlations observed for compounds **2** and **5**–**11**.

**Figure 3:**
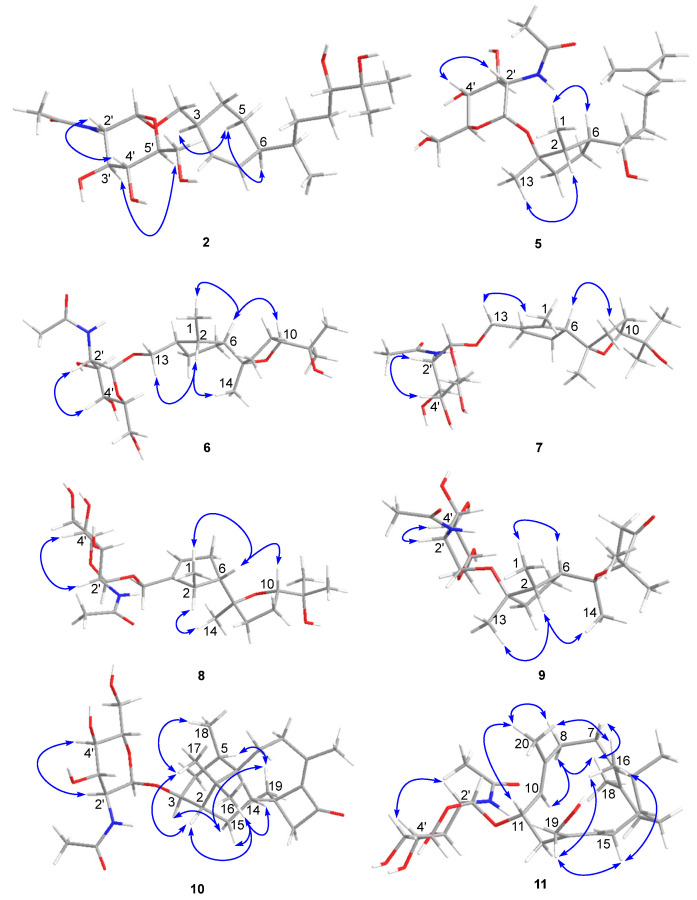
Key NOE correlations observed for compounds **2** and **5**–**11**.

Compound **2** was isolated as a colorless gum and its molecular formula was determined to be C_23_H_41_NO_8_ using HRESIMS. The ESI-MS fragment-ion peak observed at *m*/*z* 204 was indicative of the presence of an acetamido-sugar moiety [9,11,14]. The ^1^H, ^13^C, DEPT, and HSQC NMR spectra show four sp^3^ methyl, six sp^3^ methylene, nine sp^3^ methine, and two sp^2^ methine signals, as well as peaks corresponding to one sp^3^ quaternary carbon and one ketone carbonyl carbon (Table 1). Apart from the additional ^1^H NMR signals observed at δ_H_ [4.67, d (3.0) H-1’], [3.62, m H-2’], [3.45, m H-3’], [3.12, m H-4’], [3.38, m H-5’], [3.59, m H-6’a], [3.48, m H-6’b], [1.83, s Me-8’], and [7.68, d (8.0) -NH], and the ^13^C NMR signals at δ_C_ [97.1, C-1’], [54.0, C-2’], [70.6, C-3’], [70.7, C-4’], [72.8, C-5’], [60.7, C-6’], [169.4, C-7’], and [22.5, Me-8’], the NMR data were almost identical to those reported for trichobisabolin X [19], a bisabolane-type sesquiterpene, which suggests the presence of an acetamido-substituted sugar residue [9,11,14]. Unfortunately, most of the proton signals in the sugar region overlap in DMSO-*d_6_* (Table 1), and consequently the coupling constants could not be determined. The ^1^H and ^13^C NMR data were re-acquired in MeOD-*d_4_*. The chemical shifts and proton coupling constants in the sugar region [δ_H_ 4.81, d (3.0 Hz) H-1’; δ_H_ 3.86, dd (10.0, 4.0 Hz) H-2’; δ_H_ 3.62, dd (10.0, 9.0 Hz) H-3’; δ_H_ 3.36, dd (10.0, 9.0 Hz) H-4’, δ_H_ 3.58, m H-5’; δ_H_ 3.80, dd (12.0, 2.0 Hz) H-6’a; δ_H_ 3.69, dd (12.0, 5.0 Hz) H-6’b; δ_H_ 1.99, s Me-8’] are almost identical to the 2-acetamido-2-deoxy-*α*-glucopyranosyl signals in trichaspsides A–E (Table 1) [11,14]. NOE correlations observed for H-2’/H-4’ and H-3’/H-5’ further confirmed the relative configuration of the glucopyranoside. The small anomeric-proton coupling constant (3.0 Hz) suggests that the sugar linkage has an *α*-configuration. The aglycone of **2** was further analyzed using ^1^H-^1^H correlation spectroscopy (COSY) and heteronuclear multiple bond correlation (HMBC) (Figure 2). The ^1^H-^1^H COSY correlations connect C-1 to C-10 and the C13 methyl group (Figure 2). The two singlet methyl groups are not correlated in the COSY spectrum. The two methyl groups (δ_H_ 0.98 s and 1.03 s) show HMBC correlations with C-10 at δ_C_ 77.4 and another quaternary carbon at δ_C_ 71.6. One additional hydroxy group at δ_H_ 4.03 also exhibits a HMBC correlation with this quaternary carbon. Therefore, the presence of an isopropanol moiety is proposed to be linked to C-10. The aglycone moiety was determined to be the same as that observed in trichobisabolin X upon further comparison of the NMR spectra (Table 1 and Figure 2) [14]. The sugar residue was determined to be attached to C-14 by the mutual HMBC correlations observed between H-1’ and C-14, and H_2_-14 and C-1’ (Figure 2). H-3/H-5a and H-5a/H-6 ROESY correlations and the identical NMR data obtained for the aglycone suggest the same relative configuration to that observed in trichobisabolin X (Table 1 and Figure 3). Since three bisabolane acetamido glycosides, trichaspsides C–E, have been previously reported [11,14], we assigned compound **2** as trichaspside F. 

Compounds **5**–**11** were isolated as colorless gums. Their ESI-MS spectra exhibit representative base peaks at *m*/*z* 204, which imply the presence of an acetamido-sugar moiety in each structure [9,11,14]. The sugar region exhibits ^1^H and ^13^C NMR data that are almost identical to those reported for trichaspside F (**2**), which reveals the presence of a 2-acetamido-2-deoxy-*α*-glucopyranosyl moiety in each of these molecules (Table 2, Table 3 and Table 4). The small coupling constant observed for each aromatic proton (3.0 Hz) suggests all of the sugar linkages have an α-configuration (Table 2 and Table 4).

**Figure 4:**
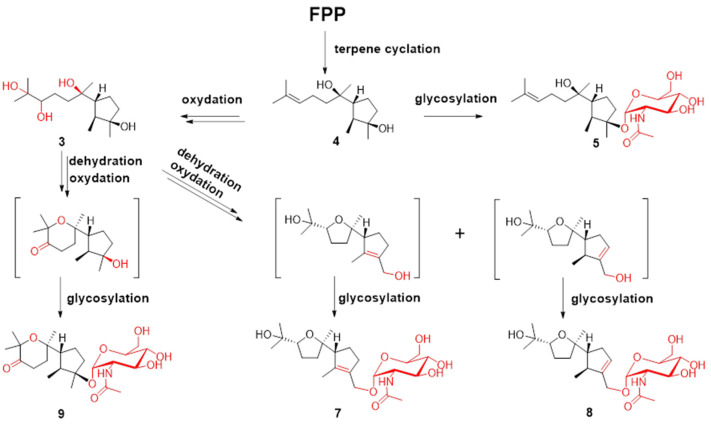
Plausible biosynthesis routes for compounds **3**–**5** and **7**–**9**.

The molecular formula of compound **5** was determined to be C_23_H_41_NO_7_ using HRESIMS. The ^1^H-^1^H COSY analysis shows that the methine at H-10 is connected to the methylene at H_2_-9, and H_2_-9 is connected with another methylene at H_2_-8. The 1-Me methyl group is connected to the methine at H-2 and extends from H-2 to H-6, H_2_-4, and H_2_-5 (Figure 2). From the HMBC spectrum, two methyl groups (δ_H_ 1.56 s, 1.63 s) are linked to the quaternary carbon at C-11 (δ_C_ 130.1). 14-Me (δ_H_ 1.00 s) exhibits HMBC correlations with C-6 and C-7. 7-OH (δ_H_ 3.89) shows HMBC correlations with C-7 and C-9. These correlations connect the partial structure between C-6 and C-9 (Figure 2). Both methyl groups at 1-Me and 13-Me show HMBC correlations with the quaternary carbon at C-3 (δ_C_ 86.3) (Figure 2). Detailed ^1^H-^1^H COSY and HMBC analyses were then conducted to establish the structure of the aglycone (Figure 2 and Figures S10 and S18). With the exception of the sugar region, compound **5** exhibits ^1^H and ^13^C NMR data that are almost identical to those reported for cyclonerodiol (**4**), a cyclonerane-type sesquiterpene (Table 2 and Table 3) [19]. HMBC correlations between H-1’ and C-3 establish that the sugar moiety is linked to C-3 (Figure 2), and the ROESY correlations observed for Me-1/H-6 and Me-13/H-2 reveal that the relative configurations of the five-membered ring are identical to those of cyclonerodiol (**4**) (Figure 3) [20]. Unfortunately, the configuration at C-7 could not be determined. Thus, compound **5** is identified to be 3-O-cyclonerodiol-2-acetamido-2-deoxy-*α*-glucopyranoside (Figure 1). As it is the first glycosylated cyclonerane-type sesquiterpene discovered to date, we have assigned compound **5** as cycloneroside A. Cyclonerodiol (**4**) was obtained in this study with a negative optical rotation of −18, which is similar to the reported −20 of [21]. Considering that cycloneroside A (**5**) and cyclonerodiol (**4**) possess the same sesquiterpene carbon skeleton and presumably the same biogenetic pathway (Figure 4), the absolute configurations of the aglycone part of cycloneroside A (**5**) were thus assigned as that of cyclonerodiol (**4**) based on the biogenic consideration as 2*S*, 3*R*, 6*R*, 7*R* [21,22,23]. The absolute configurations of the sugar moiety were not able to be assigned due to the lack of materials.

The molecular formula of compound **6** was determined to be C_23_H_41_NO_8_ using HRESIMS. Apart from the sugar region and the additional signals observed for an oxygenated methylene group [δ_H_ 3.54 m, 3.15 m; δ_C_ 68.9], the sp^3^ methine signal [δ_H_ 2.11 m, H-3; δ_C_ 43.2], and lack of signals corresponding to the methyl group at C-13 and oxygenated quaternary carbon at C-3 (Table 2 and Table 3), compound **6** exhibits NMR data that highly resemble those reported for isoepicyclonerodiol oxide [19]. The additional oxygenated methylene and methine groups are located at C-13 and C-3, respectively, based on the ^1^H-^1^H COSY correlations observed between H-13 and H-3, and H-3, H-2, and H-4, and the HMBC correlations observed between H-13 and C-3 and C-4, and between 1-Me and C-3 (Figure 2). The 2D NMR spectra were then carefully analyzed, which confirmed the presence of an aglycone, as shown in Figure 2. The sugar moiety is linked at C-13 according to the HMBC correlations observed between H-1’ and C-13, and H-13 and C-1’ (Figure 2 and Figures S19–S26). Further, the ROESY correlations observed between Me-1/H-6 and Me-13/H-2 indicate that ring A has the same relative configuration as compound **5**. The ROESY correlations observed for H-6/H-10 and H-2/Me-14 (Figure 3), and the almost identical NMR data obtained for ring B suggest the same relative configuration as isoepicyclonerodiol oxide [19]. Thus, compound **6** was identified to be a 13-O-isoepicyclonerodiol oxide-2-acetamido-2-deoxy-*α*-glucopyranoside, which we assigned as cycloneroside B. The absolute configurations of the aglycone part of cycloneroside B (**6**) were assigned based on the biogenic consideration as 2*S*, 3*S*, 6*R*, 7*R, 10R.*

The molecular formula of compound **7** was determined to be C_23_H_39_NO_8_ using HRESIMS. The IR absorption bands of amide I at 1639 cm^−1^ (C=O stretching vibration) and amide II at 1555 cm^−1^ (N-H bending vibration) indicated the presence of a secondary amide group [24]. With the exception of the sugar region, and the lack of the sp^3^ methine signals at C-2 and C-3 [δ_H_ 2.22, m, δ_C_ 35.5; δ_H_ 2.11, m, δ_C_ 43.2] and two additional sp^2^ olefinic quaternary carbons [δ_C_ 134.8; δ_C_ 137.5] (Table 2 and Table 3), the ^1^H and ^13^C NMR data obtained for **7** resemble those of **6** (Figures S27–S34). The Me-1 proton signal of compound **7** (δ_H_ 1.66) is down-field-shifted when compared to that of **6** [δ_H_ 0.68, d (7.0 Hz)] and appears as a singlet. The methylene signals at position 13 are also low-field shifted (δ_H_ 3.98 and 4.10) relative to those observed for **6** (δ_H_ 3.54, m; δ_H_ 3.15, m) with a geminal coupling observed (*J* = 12 Hz), which implies the presence of two olefinic carbons at C-2 and C-3 (Table 2). These positions were further validated using the HMBC correlations observed between 1-Me and H-13 and these two olefinic carbons (Figure 2). The sugar moiety is linked at C-13 according to the HMBC correlations observed between H-1’ and C-13, and H-13 and C-1’ (Figure 2). The ROESY correlation observed between Me-1 and H-13 suggests the presence of a trans double bond. The almost identical NMR data obtained for ring B and the H-6/H-10 ROESY correlation suggest the same relative configuration as that observed for compound **6** (Table 2 and Figure 3 and Figures S35–S43). Hence, the structure of compound **7** is elucidated (Figure 1) and assigned as cycloneroside C. The absolute configurations of the aglycone part of cycloneroside C (**7**) re assigned based on the biogenic consideration as 6*R*, 7*R, 10R.*

The molecular formula of compound **8** was determined to be C_23_H_39_NO_8_ using HRESIMS. The IR absorption bands at 1653 and 1555 cm^−1^ indicated the presence of a secondary amide group. With the exception of one additional sp^2^ methine [δ_H_ 5.54 (br s); δ_C_ 126.8, CH] and an sp^3^ methine [δ_H_ 2.46 m; δ_C_ 41.0, CH], the lack of one sp^2^ quaternary carbon at C-2 and one sp^3^ methylene group at C-4, which implies that the double bond is positioned between C-3 and C-4, compound **8** exhibits ^1^H and ^13^C NMR data that resemble those of **7** (Table 2 and Table 3). The ^1^H-^1^H COSY correlations observed between H-4 and H-5 (δ_H_ 2.32, m; δ_H_ 2.06, m) and H-2 and 1-Me [δ_H_ 1.06, d (7.0)] (Figure 3) confirm the position of the new double bond. The HMBC correlations between H-13 and C-3 and C-4, and between 1-Me and C-2 and C-3 also confirm this assignment (Figure 2). The sugar moiety is linked at C-13 according to the HMBC correlations observed between H-1’ and C-13, and H-13 and C-1’ (Figure 2). The ROESY correlation observed for Me-1/H-6 suggests that these protons are directed toward the same face of the molecule. The other ROESY correlations and NMR data obtained for ring B are almost identical to those observed for compound **6**, suggesting they have the same relative configuration (Table 2 and Figure 3). We assigned compound **8** as cycloneroside D. The absolute configurations of the aglycone part of cycloneroside D (**8**) were assigned based on the biogenic consideration as 2*S*, 6*R*, *10R.*

The molecular formula of **9** was determined to be C_23_H_39_NO_8_ using HRESIMS. The ^1^H and ^13^C NMR data obtained for the aglycone are very similar to those of 3,7,11-trihydroxycycloneran-10-one [25]. Detailed ^1^H COSY and HMBC analysis confirms the skeleton (Figure 2 and Figures S44–S51); however, MS reveals an *m*/*z* difference of 18, which suggests dehydration between the two hydroxyl groups at C-7 and C-11 (Table 2 and Table 3). The coupling patterns observed for H-8 [δ_H_ 2.04 (13.0, 10.0, 6.0); δ_H_ 1.88 (13.0, 6.0, 5.0)] and H-9 [δ_H_ 2.53 (17.0, 10.0, 6.0); δ_H_ 2.42 (17.0, 6.0, 5.0)] also suggest a relatively rigid tetrahydropyran for ring B instead of a linear structure [25]. The sugar moiety is linked to C-3 according to the HMBC correlation observed between H-1’ and C-3 (Figure 2). The almost identical NMR data obtained for ring A and the ROESY correlations observed for Me-1/H-6 and Me-13/H-2 suggest that compound **9** has the same relative configuration to that of ring A in compound **5** (Table 2 and Figure 3). The ROESY correlation observed at H-2/Me-14 indicates that the two groups are directed toward the same side of the molecule (Figure 3). Thus, the structure of **9** is elucidated (Figure 1) and assigned as cycloneroside E. The absolute configurations of the aglycone part of cycloneroside E (**9**) was assigned based on the biogenic consideration as 2*S*, 3*R*, 6*R*, *10R.*

The molecular formula of compound **10** was deduced to be C_28_H_43_NO_7_ using HRESIMS. The IR absorption bands at 1742, 1632, and 1563 cm^−1^ indicated the presence of a carbonyl and a secondary amide group. With the exception of the additional signals corresponding to the acetamido-sugar moiety, the aglycone exhibits ^1^H and ^13^C NMR data consisting of one ketone (C-11), two sp^2^ quaternary carbons (C-9 and C-10), and 17 sp^3^ carbons including five methyl (C-16, C-17, C-18, C-19, and C-20), five methylene (C-4, C-7, C-8, C-12, and C-15), one oxygenated methine (C-3), three methines (C-2, C-5, and C-14), and three non-protonated carbons (C-1, C-6, and C-13) by HSQC and DEPT analyses (Table 4). Careful examination of the NMR data, particularly ^1^H-^1^H COSY and HMBC correlations (Figure 2), confirmed the presence of a harziane-type tetracyclic scaffold consisting of a 6/5/7/4-fused tetra-cyclic ring system. The NMR data resemble those of 3*S*-hydroxyharzianone, a harziane-type diterpene (Table 4 and Figures S52–S58) [26,27], except for the additional amino sugar moiety. The sugar moiety is attached to C-3 according to the HMBC correlation observed between H-1’ and C-3 (Figure 2), and the relative configurations of compound **10** are fully established using ROESY, which exhibit correlations for Me-18/Me-17, Me-17/H-2, Me-16/H-2, Me-16/H-14, and Me-16/H-15a. This further suggests that these protons point toward the same side of the molecule, whereas the ROESY correlations observed for H-3/H-15b, Me-19/ H-15b, and Me-19/H-5 indicate that these protons are placed on the other side of the molecule (Figure 3). This compound is assigned as harzianoside A. 

The molecular formula of compound **11** was determined to be C_28_H_45_NO_7_ using HRESIMS. With the exception of the sugar moiety, the aglycone exhibits ^1^H and ^13^C NMR data comprised six sp^2^ carbons including four quaternary double bond carbons (C-5, C-6, C-9, and C-13) and two methines (C-10 and C-14); and 14 sp^3^ carbons including one quaternary carbon (C-1), one oxygenated methine (C-11), one oxygenated methylene (C-19), one methine (C-2), six methylene (C-3, C-4, C-7, C-8, C-12, and C-15), and four methyl (C-16, C-17, C-18, and C-20) groups by HSQC and DEPT analyses (Table 4). Detailed ^1^H-^1^H COSY and HMBC analyses reveal the bicyclic core structure of **11**, which contains cyclohexene and cyclododecadiene rings (Figure 2 and Figures S59–S65). The aglycone exhibits ^1^H and ^13^C NMR data that are almost identical to those of 11R-methoxy-5,9,13-proharzitrien-19-ol (Table 4) [28]. The sugar moiety is linked to C-11 according to the HMBC correlation observed for H-1’/C-11 (Figure 2). The Me-18/H-7, Me-20/H-11, and H-15/H-19 ROESY correlations suggest the presence of 5-6-cis, 9-10-trans, and 13-14-cis double bonds. The NOE correlations observed for Me-20/Me-16, Me-20/H-8b, M-20/H-11, Me-16/H-15, and Me-16/H-19 suggest that these protons are directed to the same side of the molecule, whereas the NOE correlations for Me-18/H-8a and H-8a/H-10 indicate that these protons point to the other side of the molecule (Figure 3). Therefore, the structure of compound **11** is elucidated (Figure 1) and assigned as harzianoside B. 

The structures of the three known sesquiterpenes, trichodermoside (**1**) [10], cycloneran-3,7,10,11-tetraol (**3**) [25], and cyclonerodiol (**4**) [20], were determined by comparison with the data reported in the literature. 

Terpenes are biosynthesized from C-5 building blocks composed of isopentenyl diphosphate (IPP) and dimethylallyl diphosphate (DMAPP). Prenyl transferase assembles IPP and DMAPP into farnesyl diphosphate (FPP), which is the universal precursor for the sesquiterpenes. FPP is cyclized by various terpene cyclases to form different types of sesquiterpenes. The post-tailoring steps further diversify these structures [29,30]. The plausible biosynthesis routes to cyclonerane-type sesquiterpenes in this study are summarized in Figure 4. Cyclonerodiol (**4**) has been demonstrated to be the direct product of FPP cyclization by terpene cyclase [22]. The reactions observed in the post tailoring steps may include complex oxidation, dehydration, and glycosylation (Figure 4). Notably, the N-acetylaminosugar usually needs to undergo additional transamination and acetylation reactions prior to the glycosylation reaction [31,32].

**Figure 5:**
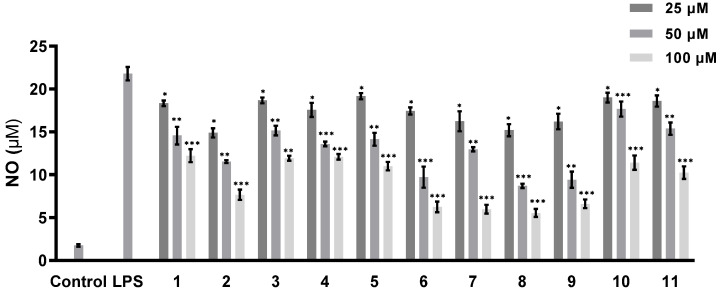
NO-production-inhibitory activities of compounds **1**–**11**. The values represent the mean ± SEM of three independent experiments. * *p* < 0.05; ** *p* < 0.01; *** *p* < 0.001.

**Figure 6:**
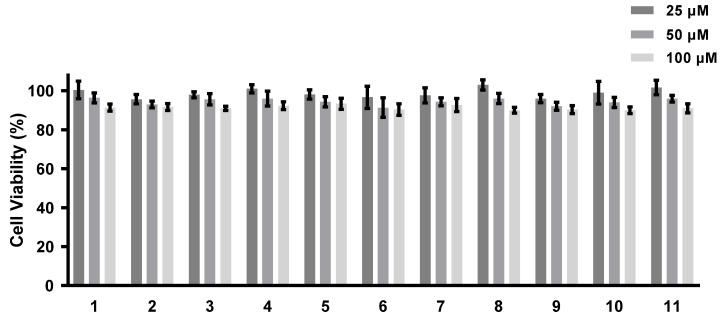
Cell viability of compounds **1**–**11**. The values represent the mean ± SEM of three independent experiments.

The abilities of all of the isolated compounds to inhibit NO production were examined using macrophage RAW 264.7 cells stimulated by LPS [18]. Figure 5 shows that all of the compounds inhibit NO production in a dose-dependent manner. None of them exhibit cytotoxicity at the maximum tested concentration (100 µM) (Figure 6). A comparison of the data obtained for cyclonerodiol (**4**) and cycloneroside A (**5**) suggests that the sugar moiety appears to have little impact on the activity of this type of compound. Trichaspside F (**2**) and cyclonerosides B–E (**6**–**9**) exhibit the strongest abilities to inhibit NO production with IC_50_ values of 54.8, 50.7, 57.1, 42.0, and 48.0 µM, respectively. The other compounds in this study show low activity with IC_50_ values ~100 µM. Our structure–activity relationship analyses reveal that the tetrahydropyran or tetrahydrofuran ring is essential toward improving the activities of cyclonerosides B–E (**6**–**9**) when compared with those of cycloneroside A (**5**), which contains a linear prenyl chain on the right hand side of the structure. The positive control (quercetin) exhibits an NO-production-inhibitory effect with an IC_50_ value of 30.8 µM. 

Inflammatory responses are known to be deeply associated with the pathogenesis of various diseases, such as diabetes, cardiovascular diseases, obesity, arthritis, stroke, and cancer [17]. The over-expression of cellular transduction molecules, such as NO, histamine, and other pro-inflammatory cytokines, leads to chronic inflammation, which eventually induces inflammation-related diseases. Chemical inhibitors of cell signal transduction that can inhibit the excessive release of these harmful cytokines should be useful as potential drug candidates [17,33]. There has been a large number of reports on natural products with NO-production-inhibitory activity isolated from plants, most of which are phenolic-type components [33]. To the best of our knowledge, there have been no reports on the NO-production-inhibitory activities of sesquiterpene aminoglycosides. These compounds do not show any cytotoxicity at the highest test concentration, and further structural modification of these compounds may lead to new, potent, and safe anti-inflammatory drugs.

The anti-fungal activities were also tested against plant pathogenic fungi including *Helminthosporium maydis*, *Gibberella sanbinetti*, and *Penicillium digitatum*. None of the compounds exhibit any obvious activity at a concentration of 100 µg/mL.

## 3. Materials and Methods

### 3.1. General Experimental Procedures

NMR spectroscopy was performed using a Bruker ASCEND 600 MHz NMR spectrometer equipped with a CryoProbe (Bruker Biospin GmbH, Rheinstetten, Germany). Optical rotations were measured on an Anton Paar MCP-100 polarimeter (Anton Paar GmbH, Graz, Austria) and the HRESIMS spectra were acquired on a MaXis quadrupole-time-of-flight mass spectrometer (Bruker Biospin GmbH, Rheinstetten, Germany). Silica gel (200–300 mesh, Qingdao Haiyang Chemical, Qingdao, China) and YMC Gel ODS-A (150 µM, YMC Co., Ltd., Kyoto, Japan) were used for column chromatography and silica gel 60 F_254_ and RP-18 F_254_ thin-layer chromatography (TLC) plates (Merck Millipore Co., Darmstadt, Germany) were used for TLC. HPLC was performed using a Shimadzu LC-16P system (Shimadzu Co., Tokyo, Japan) equipped with a YMC-Pack ODS-A C_18_ column (20 × 250 mm, 5 µm; YMC Co., Ltd., Kyoto, Japan). Analytical- and HPLC-grade reagents (Macklin Co., Shanghai, China) were used for the isolation procedures. 

### 3.2. Fungal Strain and Fermentation

The *Trichoderma* sp. SCSIOW21 fungal strain was collected from deep-sea sediments and deposited at the Laboratory of Microbial Natural Products, Shenzhen University, China. The fungal strain was identified as *Trichoderma* species according to its morphological characteristics and ITS gene sequence (OP854922). The fungal strain was cultivated statically for 30 d in rice broth containing 3% sea salt. The culture was extracted using an equal volume of n-BuOH. 

### 3.3. Isolation Procedure

The n-BuOH extract was subjected to silica gel column chromatography with gradient elution using CH_2_Cl_2_/MeOH/water (100:0:0, 50:1:0, 20:1:0, 10:1:0, 5:1:0.1, 3:1:0.1, 1:1:0.1, and 0:0:100, *v*/*v*/*v*, 2.0 L of each) to yield eight fractions (A–H). Fraction B was separated using a medium-pressure YMC Gel ODS-A (150 µM) column with a MeOH/water gradient (50–100% MeOH) to give five sub-fractions. Subfraction 3 was separated using a YMC-Pack ODS-A C_18_ column [acetonitrile (ACN)/water, 50:50 *v/v*] to give compound **4** (*t*_R_ 19.1 min, 10 mL/min, 0.7 mg). Fraction E was separated using a medium-pressure YMC Gel ODS-A (150 µM) column with a MeOH/water gradient (10–100% MeOH) to give 18 sub-fractions. Subfraction 9 was separated using a YMC-Pack ODS-A C_18_ column (ACN/water, 12:88 *v/v*) to yield compound **3** (*t*_R_ 19.1 min, 10 mL/min, 0.7 mg). Subfraction 15 was separated using HPLC on a YMC-Pack ODS-A C_18_ column (ACN/water, 23:77 *v/v*) to yield compound **1** (*t*_R_ 24.7 min, 10 mL/min, 1.9 mg). Subfraction 16 was separated on the YMC-Pack ODS-A C_18_ column (ACN/water, 25:75) to give compounds **2**, **7**, **8**, and **9** (*t*_R2_ 20.7 min, 10 mL/min, 1.4 mg; *t*_R7_ 24.6 min, 10 mL/min, 3.1 mg; *t*_R8_ 26.1 min, 10 mL/min, 2.8 mg; *t*_R9_ 31.0 min, 10 mL/min, 2.8 mg). Subfraction 17 was separated using a YMC-Pack ODS-A C_18_ column (ACN-Water, 33:67 *v/v*) to yield compounds **5**, **6**, and **10** (*t*_R5_ 16.5 min, 10 mL/min, 0.6 mg; *t*_R6_ 22.0 min, 10 mL/min, 0.6 mg; *t*_R10_ 33.5 min, 10 mL/min, 2.0 mg). Subfraction 18 was separated on the YMC-Pack ODS-A C_18_ column (ACN/water, 40:60 *v/v*) to yield compound **11** (*t*_R_ 31.5 min, 10 mL/min, 1.9 mg).

### 3.4. Spectral Data

Trichaspside F (**2**): Colorless gum; ${\left[\alpha \right]}_{D}^{25}$ + 32 (*c* 0.17, MeOH); ^1^H and ^13^C NMR data (DMSO-*d*_6_, 600 and 150 MHz, respectively), see Table 1; HRESIMS *m*/*z*: 482.2744 [M + Na]^+^ (calcd. for C_23_H_41_NNaO_8_, 482.2730).

Cycloneroside A (**5**): Colorless gum; ${\left[\alpha \right]}_{D}^{25}$ + 74 (*c* 0.14, MeOH); ^1^H and ^13^C NMR data (DMSO-*d*_6_, 600 and 150 MHz, respectively), see Table 2 and Table 3; HRESIMS *m*/*z*: 466.2769 [M + Na]^+^ (calcd. for C_23_H_41_NNaO_7_, 466.2781).

Cycloneroside B (**6**): Colorless gum; ${\left[\alpha \right]}_{D}^{25}$ + 28 (*c* 0.21, MeOH); ^1^H and ^13^C NMR data (DMSO-*d*_6_, 600 and 150 MHz, respectively), see Table 2 and Table 3; HRESIMS *m*/*z*: 482.2743, [M + Na]^+^ (calcd. for C_23_H_41_NNaO_8_, 482.2730).

Cycloneroside C (**7**): Colorless gum; ${\left[\alpha \right]}_{D}^{25}$ + 104 (*c* 0.26, MeOH); IR (KBr) *v*_max_ 3355, 2970, 1639, 1555, 1437, 1377, 1317, 1022, 950 cm^−1^; ^1^H and ^13^C NMR data (DMSO-*d*_6_, 600 and 150 MHz, respectively), see Table 2 and Table 3; HRESIMS *m*/*z*: 480.2581 [M + Na]^+^ (calcd. for C_23_H_39_NNaO_8_, 480.2573).

Cycloneroside D (**8**): Colorless gum; ${\left[\alpha \right]}_{D}^{25}$ + 20 (*c* 0.5, MeOH); IR (KBr) *v*_max_ 3348, 2927, 1653, 1551, 1443, 1373, 1331, 1013 cm^−1^; ^1^H and ^13^C NMR data (DMSO-*d*_6_, 600 and 150 MHz, respectively), see Table 2 and Table 3; HRESIMS *m*/*z*: 480.2576 [M + Na]^+^ (calcd. for C_23_H_39_NNaO_8_, 480.2573).

Cycloneroside E (**9**): Colorless gum; ${\left[\alpha \right]}_{D}^{25}$ + 56 (*c* 0.14, MeOH); ^1^H and ^13^C NMR data (DMSO-*d*_6_, 600 and 150 MHz, respectively), see Table 2 and Table 3; HRESIMS *m*/*z*: 480.2580, [M + Na]^+^ (calcd. for C_23_H_39_NNaO_8_, 480.2573).

Harzianoside A (**10**): Colorless gum; ${\left[\alpha \right]}_{D}^{25}$ + 91 (*c* 0.81, MeOH); IR (KBr) *v*_max_ 3402, 2929, 1742, 1632, 1563, 1436, 1379, 1327, 1046, 972 cm^−1^; ^1^H and ^13^C NMR data (DMSO-*d*_6_, 600 and 150 MHz, respectively), see Table 4; HRESIMS *m*/*z*: 506.3117 [M + H]^+^ (calcd. for C_28_H_44_NO_7_, 506.3118).

Harzianoside B (**11**): Colorless gum; ${\left[\alpha \right]}_{D}^{25}$ + 31 (*c* 0.15, MeOH); ^1^H and ^13^C NMR data (DMSO-*d*_6_, 600 and 150 MHz, respectively), see Table 4; HRESIMS *m*/*z*: 530.3084 [M + Na]^+^ (calcd. for C_28_H_45_NNaO_7_, 530.3094).

### 3.5. NO-Production-Inhibitory Activity

NO inhibition was investigated using LPS-stimulated RAW 264.7 cells [18]. Briefly, RAW264.7 cells were plated into a 96-well plate at a density of 1 × 10^5^ cells/well. The tested samples were simultaneously added with LPS stimulation at final concentrations of 25, 50, and 100 μM. The cells were cultivated for 24 h and the supernatant tested for NO using the Griess reagent. Quercetin was used as a positive control with an IC_50_ value of 30.8 µM. Cells incubated for 4 h were subjected to an MTT assay [18]. 

### 3.6. Anti-fungal Activity

The mycelial-growth-inhibition effects were tested based on an assay protocol used in a previous publication [23]. 

## 4. Conclusions

Glycosylated terpenoids from the *Trichoderma* species of filamentous fungi have rarely been reported. In this study, eleven terpenes, including six new sesquiterpene and two new diterpene aminoglycosides, were isolated and characterized from a deep-sea-sediment-derived fungus, *Trichoderma* sp. SCSIOW21. Cyclonerosides A–E (**5**–**9**) represent the first glycosylated cyclonelane-type sesquiterpenes isolated from *Trichoderma*, which greatly increase the chemo-diversity of this species. The NO-production-inhibitory activities were tested for each of the isolated compounds. Trichaspside F (**2**) and cyclonerosides B–E (**6**–**9**) exhibit the strongest abilities to inhibit NO production with IC_50_ values of 54.8, 50.7, 57.1, 42.0, and 48.0 µM, respectively. Further studies on the structure–activity relationship and mechanism of action of these compounds may contribute to the development of new anti-inflammatory drugs.

## Figures and Tables

**Table 1:** ^1^H and ^13^C NMR data (600 and 150 MHz, respectively) obtained for compound **2**.

Position	2 (m, *J* in Hz) in DMSO-*d_6_*		2 (m, *J* in Hz) in MeOD-*d_4_*	
1	133.1, CH	5.61, d (10.0)	134.5, CH	5.70, brd (10.0)
2	127.8, CH	5.64, ddd (10.0, 5.0, 2.0)	129.0, CH	5.68, brd (10.0)
3	34.2, CH	2.31, brs	36.1, CH	2.39, m
4a	23.2, CH_2_	1.65, m	24.9, CH_2_	1.74, m
4b		1.54, m		1.65, m
5a	19.9, CH_2_	1.31, m ^a^	21.6, CH_2_	1.52, m
5b		1.43, m ^a^		1.44, m
6	40.0, CH	2.01, m	41.7, CH	2.09, m
7	36.4, CH	1.45, m ^a^	38.2, CH	1.52, m
8a	30.9, CH_2_	1.27, m ^a^	32.3, CH_2_	1.44, m
8b		1.36, m ^a^		1.44, m
9a	28.5, CH_2_	1.48, m ^a^	30.0, CH_2_	1.58, m
9b		1.15, m		1.34, m
10	77.4, CH	3.03, dd (10.0, 2.0)	79.6, CH	3.23, dd (10.0, 2.0)
10-OH		4.26, d (6.0)		
11	71.6, C		73.8, C	
11-OH		4.03, s		
12	24.4, CH_3_	0.98, s	24.9, CH_3_	1.13, s
13	15.8, CH_3_	0.77, d (7.0)	16.4, CH_3_	0.87, d (7.0)
14a	70.3, CH_2_	3.15, dd (10.0, 5.0)	72.4, CH_2_	3.25, dd (10.0, 5.0)
14b		3.12, dd (10.0, 8.0)		3.24, dd (10.0, 8.0)
15	26.4, CH_3_	1.03, s	25.7, CH_3_	1.16, s
1’	97.1, CH	4.67, d (3.0)	98.9, CH	4.81, d (3.0)
2’	54.0, CH	3.62, m ^a^	55.6, CH	3.86, dd (10.0, 4.0)
3’	70.6, CH	3.45, m ^a^	72.7, CH	3.62, dd (10.0, 9.0)
3’-OH		4.73, d (6.0)		
4’	70.7, CH	3.12, m ^a^	72.3, CH	3.36, dd (10.0, 9.0)
4’-OH		5.01, d (5.0)		
5’	72.8, CH	3.38, m ^a^	73.7, CH	3.58, m
6’a	60.7, CH_2_	3.59, m ^a^	62.6, CH_2_	3.80, dd (12.0, 2.0)
6’b		3.48, m ^a^		3.69, dd (12.0, 5.0)
6’-OH		4.49, t (6.0)		
7’	169.4, C		173.6, C	
8’	22.5, CH_3_	1.83, s	22.5, CH_3_	1.99, s
-NH		7.68, d (8.0)		

^a^ Overlapped signals.

**Table 2:** ^1^H NMR data (600 MHz) obtained for compounds **5**–**9**^a^.

Position	5 (m, *J* in Hz)	6 (m, *J* in Hz)	7 (m, *J* in Hz)	8 (m, *J* in Hz)	9(m, *J* in Hz)
1	1.01, d (7.0)	0.68, d (7.0)	1.66, brs	1.06, d (7.0)	1.02, d (7.0)
2	1.58, m	2.22, m		2.46, m ^b^	1.53, m
3		2.11, m			
4a	1.71, m ^b^	1.59, m ^b^	2.23, m	5.54, s	1.93, m ^b^
4b	1.47, m	1.56, m ^b^			1.26, m
5a	1.89, m	1.62, m ^b^	1.69, m	2.32, m	1.80, m ^b^
5b	1.22, m ^b^	1.59, m ^b^	1.75, m ^b^	2.06, m	1.47, m
6	1.71, m ^b^	1.92, m	2.69, ddd (10.0, 6.0, 4.0)	2.01, ddd (9.0, 6.0, 3.0)	1.96, m ^b^
7-OH	3.89, s				
8a	1.32, m	1.83, m ^b^	1.60, m ^b^	1.74, m ^b^	2.04, ddd (13.0, 10.0, 6.0)
8b	1.32, m	1.57, m ^b^	1.69, m ^b^	1.57, ddd (12.0, 8.0, 4.0 )	1.88, ddd (13.0, 6.0, 5.0)
9a	1.94, m	1.68, m ^b^	1.64, m ^b^	1.74, m ^b^	2.53, ddd (17.0, 10.0, 6.0)
9b	1.94, m	1.65, m ^b^	1.72, m ^b^	1.75, m ^b^	2.42, ddd (17.0, 6.0, 5.0)
10	5.08, dd (7.0, 1.0)	3.56, dd (10.0, 5.0)	3.52, dd (10.0, 5.0)	3.61, dd (10.0, 5.0 )	
12	1.56, s	1.02, s	1.02, s	1.02, m ^b^	1.19, s
13a	1.02, s	3.54, m ^b^	3.98, br d (12.0)	3.98, m	1.04, s
13b		3.15, m ^b^	4.10, br d (12.0)		
14	1.00, s	1.15, s	1.01, s	1.01, s	1.11, s
15	1.63, s	1.04, s	1.03, s	1.02, m ^b^	1.20, s
1’	4.91, d (3.0)	4.63, d (3.0)	4.58, d (3.0)	4.60, d (3.0)	4.92, d (3.0)
2’	3.49, m ^b^	3.58, m ^b^	3.61, m ^b^	3.64, m ^b^	3.49, m ^b^
3’	3.50, m ^b^	3.47, m ^b^	3.46, m ^b^	3.46, m ^b^	3.48, m ^b^
3’-OH	4.11, q (5.0)				
4’	3.17, m ^b^	3.10, m ^b^	3.12, dd (9.8, 8.5)	3.13, dd (9.9, 8.5)	3.15, m
4’-OH	4.60, d (5.0)				
5’	3.40, m ^b^	3.36, m ^b^	3.37, m ^b^	3.35, m ^b^	3.46, m ^b^
6’a	3.53, m ^b^	3.47, dd (12.0, 2.0)	3.49, m ^b^	3.46, m ^b^	3.50, dd (12.0, 2.0)
6’b	3.53, m ^b^	3.61, m ^b^	3.63, m	3.61, m ^b^	3.55, m ^b^
6’-OH	4.40, t (6.0)				
8’	1.81, s	1.81, s	1.80, s	1.80, s	1.81, s
-NH	7.52, d (8.0)	7.67, d (8.0)	7.72, d (8.0)	7.72, d (8.0)	7.55, d (8.0)

^a^ Recorded in DMSO-*d_6_*. ^b^ Overlapped signals.

**Table 3:** ^13^C NMR data (150 MHz) obtained for compounds **5**–**9**^a^.

Position	5	6	7	8	9
1	15.0, CH_3_	9.11, CH_3_	14.1, CH_3_	20.3, CH_3_	14.3, CH_3_
2	45.5, CH	35.5, CH	134.8, C	41.0, CH	46.4, CH
3	86.3, C	43.2, CH	137.5, C	143.5, C	86.2, C
4	23.7, CH_2_	25.3, CH_2_	32.8, CH_2_	126.8, CH	35.7, CH_2_
5	36.0, CH_2_	21.2, CH_2_	26.2, CH_2_	33.6, CH_2_	24.2, CH_2_
6	53.7, CH	53.5, CH	59.2, CH	54.8, CH	54.6, CH
7	72.6, C	82.7, C	85.5, C	85.0, C	75.7, C
8	41.0, CH_2_	37.5, CH_2_	34.5, CH_2_	34.1, CH_2_	30.5, CH_2_
9	22.4, CH_2_	24.0, CH_2_	25.3, CH_2_	26.1, CH_2_	32.3, CH_2_
10	125.1, CH	87.7, CH	85.4, CH	86.3, CH	214.2, C
11	130.1, C	69.7, C	69.7, C	69.9, C	78.5, C
12	17.4, CH_3_	25.4, CH_3_	25.5, CH_3_	25.5, CH_3_	27.5, CH_3_^b^
13	21.4, CH_3_	68.9, CH_2_	61.6, CH_2_	62.3, CH_2_	21.3, CH_3_
14	24.9, CH_3_	27.1, CH_3_	25.0, CH_3_	25.2, CH_3_	23.0, CH_3_
15	25.5, CH_3_	26.8, CH_3_	26.5, CH_3_	26.3, CH_3_	27.4, CH_3_^b^
1’	91.4, CH	97.1, CH	94.6, CH	94.4, CH	91.0, CH
2’	54.7, CH	54.1, CH	53.8, CH	53.7, CH	54.7, CH
3’	70.2, CH	70.5, CH	70.5, CH	70.6, CH	70.2, CH
4’	70.6, CH	70.9, CH	70.9, CH	70.8, CH	70.7, CH
5’	72.6, CH	72.7, CH	72.8, CH	72.8, CH	73.2, CH
6’	60.7, CH_2_	60.9, CH_2_	60.9, CH_2_	60.9, CH_2_	60.8, CH_2_
7’	169.4, C	169.4, C	169.4, C	169.3, C	169.5, C
8’	22.5, CH_3_	22.5, CH_3_	22.5, CH_3_	22.6, CH_3_	22.5, CH_3_

^a^ Recorded in DMSO-*d*_6_. ^b^ Interchangeable signals.

**Table 4:** ^1^H and ^13^C NMR data (600 and 150 MHz, respectively) obtained for compounds **10** and **11** ^a^.

Position	10		11	
1	45.2, C		36.6, C	
2	47.5, CH	1.79, dd (8.0, 3.0)	42.0, CH	1.57, m
3a	77.8, CH	3.64, dd (6.0, 3.0)	26.8, CH_2_	2.09, m
3b				1.51, m
4a	29.7, CH_2_	2.15, m	30.8, CH_2_	2.24, m
4b		1.27, m ^b^		1.85, m
5	27.3, CH	2.38, m ^b^	125.9, C	
6	50.0, C		135.6, C	
7a	29.3, CH	1.86, m ^b^	25.4, CH_2_	2.16, m
7b		1.22, m		
8a	27.3, CH_2_	2.33, m ^b^	38.2, CH_2_	2.42, dd (10.0, 4.0)
8b		1.90, m ^b^		1.94, dt (12.0, 4.0)
9	145.3, C		138.4, C	
10	149.7, C		127.6, CH	4.57, brs
11	198.0, C		70.0, CH	4.28, td (11.0, 3.0)
12a	59.2, CH_2_	2.61, d (16.0)	41.6, CH_2_	2.56, dd (11.0, 5.0)
12b		2.26, d (16.0)		1.85, brd (11.0)
13	40.0, C		133.1, C	
14	50.9, CH	2.10, m ^b^	130.6, CH	5.25, dd (12.0, 3.0)
15a	27.0, CH_2_	1.84, m	33.0, CH_2_	2.72, ddd (16.0, 12.0, 6.0)
15b		1.03, dd (14.0, 9.0)		1.82, m
16	26.5, CH_3_	0.83, s	24.0, CH_3_	0.78, s
17	23.6, CH_3_	1.24, s	32.6, CH_3_	0.91, s
18	20.4, CH_3_	1.06, d (7.0)	21.5, CH_3_	1.65, s
19a	21.1, CH_3_	1.37, s	57.9, CH_2_	3.98, d (12.0)
19b				3.69, d (12.0)
20	21.9, CH_3_	2.01, s	16.8, CH_3_	1.53, brs
1’	94.7, CH	4.85, d (3.0)	93.4, CH	4.56, d (3.0)
2’	54.7, CH	3.46, m ^b^	53.6, CH	3.61, m
3’	70.2, CH	3.15, m	70.8, CH	3.16, m
4’	70.7, CH	3.48, m	70.7, CH	3.44, m
5’	73.1, CH	3.50, m	72.6, CH	3.46, m
6’a	60.9,CH_2_	3.61, m ^b^	60.7, CH_2_	3.58, m
6’b		3.47, m		3.51, dd (12.0, 5.0)
7’	169.3, C		169.2, C	
8’	22.5, CH_3_	1.81, s	22.6, CH_3_	1.79, s
-NH		7.58, d (8.0)		7.49, d (8.0)

^a^ Recorded in DMSO-*d_6_*. ^b^ Overlapped signals.

## Data Availability

The authors confirm that the data supporting the findings of this study are available within the article and its Supplementary Materials.

## Supplementary Materials

The following are available online at https://www.mdpi.com/article/10.3390/md21010007/s1, Figures S1–S65: NMR and MS spectra of compounds **2**, and **5**–**11**.
