# Supplementary material for: Novel Sesquiterpene and Diterpene Aminoglycosides from the Deep-Sea-Sediment Fungus Trichoderma sp. SCSIOW21
Source: Mar Drugs. 2022 Dec 22;21(1):7. doi: 10.3390/md21010007 (PMC9863909; doi:10.3390/md21010007)
Supplement: Supplementary file 1 [file marinedrugs-21-00007-s001.zip › marinedrugs-2056204-supplementary.pdf]

Novel Sesquiterpene and Diterpene Aminoglycosides from the Deep-Sea-Sediment Fungus *Trichoderma* sp. SCSIOW21

Hongxu Li [1,2], Xinyi Liu [1], Zhangli Hu[1,2] and Liyan Wang [1,*]

[1] Shenzhen Key Laboratory of Marine Bioresource and Eco-environmental science, College of Life Sciences and Oceanography, Shenzhen University, Shenzhen 518060, China;

[2] Key Laboratory of Optoelectronic Engineering, Shenzhen University, Shenzhen 518060, China

* Correspondence: lwang@szu.edu.cn; Tel.: +86-755-2601-2653

List of Supporting Information

Figure S1. ^{1}H NMR (600 MHz, DMSO-d_6) spectrum of compound **2**.

Figure S2. ^{13}C NMR (150 MHz, DMSO-d_6) spectrum of compound **2**.

Figure S3. DEPT (150 MHz, DMSO-d_6) spectrum of compound **2**.

Figure S4. ^{1}H-^{1}H COSY (DMSO-d_6) spectrum of compound **2**.

Figure S5. HSQC (DMSO-d_6) spectrum of compound **2**.

Figure S6. HMBC (DMSO-d_6) spectrum of compound **2**.

Figure S7. ROESY (DMSO-d_6) spectrum of compound **2**.

Figure S8. HRESIMS spectrum of compound **2**.

Figure S9. HRESIMS spectrum of compound **2**.

Figure S10. ^{1}H NMR (600 MHz, DMSO-d_6) spectrum of compound **5**.

Figure S11. ^{13}C NMR (150 MHz, DMSO-d_6) spectrum of compound **5**.

Figure S12. DEPT (150 MHz, DMSO-d_6) spectrum of compound **5**.

Figure S13. ^{1}H-^{1}H COSY (DMSO-d_6) spectrum of compound **5**.

Figure S14. HSQC (DMSO-d_6) spectrum of compound **5**.

Figure S15. HMBC (DMSO-d_6) spectrum of compound **5**.

Figure S16. ROESY (DMSO-d_6) spectrum of compound **5**.

Figure S17. HRESIMS spectrum of compound **5**.

Figure S18. HRESIMS spectrum of compound **5**.

Figure S19. ^{1}H NMR (600 MHz, DMSO-d_6) spectrum of compound **6**.

Figure S20. ^{13}C NMR (150 MHz, DMSO-d_6) spectrum of compound **6**.

Figure S21. DEPT (150 MHz, DMSO-d_6) spectrum of compound **6**.

Figure S22. ^{1}H-^{1}H COSY (DMSO-d_6) spectrum of compound **6**.

Figure S23. HSQC (DMSO-d_6) spectrum of compound **6**.

Figure S24. HMBC (DMSO-d_6) spectrum of compound **6**.

Figure S25. ROESY (DMSO-d_6) spectrum of compound **6**.

Figure S26. HRESIMS spectrum of compound **6**.

Figure S27. ^{1}H NMR (600 MHz, DMSO-d_6) spectrum of compound **7**.

Figure S28. ^{13}C NMR (150 MHz, DMSO-d_6) spectrum of compound **7**.

Figure S29. DEPT (150 MHz, DMSO-d_6) spectrum of compound **7**.

Figure S30. ^{1}H-^{1}H COSY (DMSO-d_6) spectrum of compound **7**.

Figure S31. HSQC (DMSO-d_6) spectrum of compound **7**.

Figure S32. HMBC (DMSO-d_6) spectrum of compound **7**.

Figure S33. ROESY (DMSO-d_6) spectrum of compound **7**.

Figure S34. HRESIMS spectrum of compound **7**.

Figure S35. ^{1}H NMR (600 MHz, DMSO-d_6) spectrum of compound **8**.

Figure S36. ^{13}C NMR (150 MHz, DMSO-d_6) spectrum of compound **8**.

Figure S37. DEPT (150 MHz, DMSO-d_6) spectrum of compound **8**.

Figure S38. ^{1}H-^{1}H COSY (DMSO-d_6) spectrum of compound **8**.

Figure S39. HSQC (DMSO-d_6) spectrum of compound **8**.

Figure S40. HMBC (DMSO-d_6) spectrum of compound **8**.

Figure S41. ROESY (DMSO-d_6) spectrum of compound **8**.

Figure S42. HRESIMS spectrum of compound **8**.

Figure S43. HRESIMS spectrum of compound **8**.

Figure S44. ^{1}H NMR (600 MHz, DMSO-d_6) spectrum of compound **9**.

Figure S45. ^{13}C NMR (150 MHz, DMSO-d_6) spectrum of compound **9**.

Figure S46. DEPT (150 MHz, DMSO-d_6) spectrum of compound **9**.

Figure S47. ^{1}H-^{1}H COSY (DMSO-d_6) spectrum of compound **9**.

Figure S48. HSQC (DMSO-d_6) spectrum of compound **9**.

Figure S49. HMBC (DMSO-d_6) spectrum of compound **9**.

Figure S50. ROESY (DMSO-d_6) spectrum of compound **9**.

Figure S51. HRESIMS spectrum of compound **9**.

Figure S52. ^{1}H NMR (600 MHz, DMSO-d_6) spectrum of compound **10**.

Figure S53. ^{13}C NMR (150 MHz, DMSO-d_6) spectrum of compound **10**.

Figure S54. ^{1}H-^{1}H COSY (DMSO-d_6) spectrum of compound **10**.

Figure S55. HSQC (DMSO-d_6) spectrum of compound **10**.

Figure S56. HMBC (DMSO-d_6) spectrum of compound **10**.

Figure S57. ROESY (DMSO-d_6) spectrum of compound **10**.

Figure S58. HRESIMS spectrum of compound **10**.

Figure S59. ^{1}H NMR (600 MHz, DMSO-d_6) spectrum of compound **11**.

Figure S60. ^{13}C NMR (150 MHz, DMSO-d_6) spectrum of compound **11**.

Figure S61. ^{1}H-^{1}H COSY (DMSO-d_6) spectrum of compound **11**.

Figure S62. HSQC (DMSO-d_6) spectrum of compound **11**.

Figure S63. HMBC (DMSO-d_6) spectrum of compound **11**.

Figure S64. ROESY (DMSO-d_6) spectrum of compound **11**.

Figure S65. HRESIMS spectrum of compound **11**.

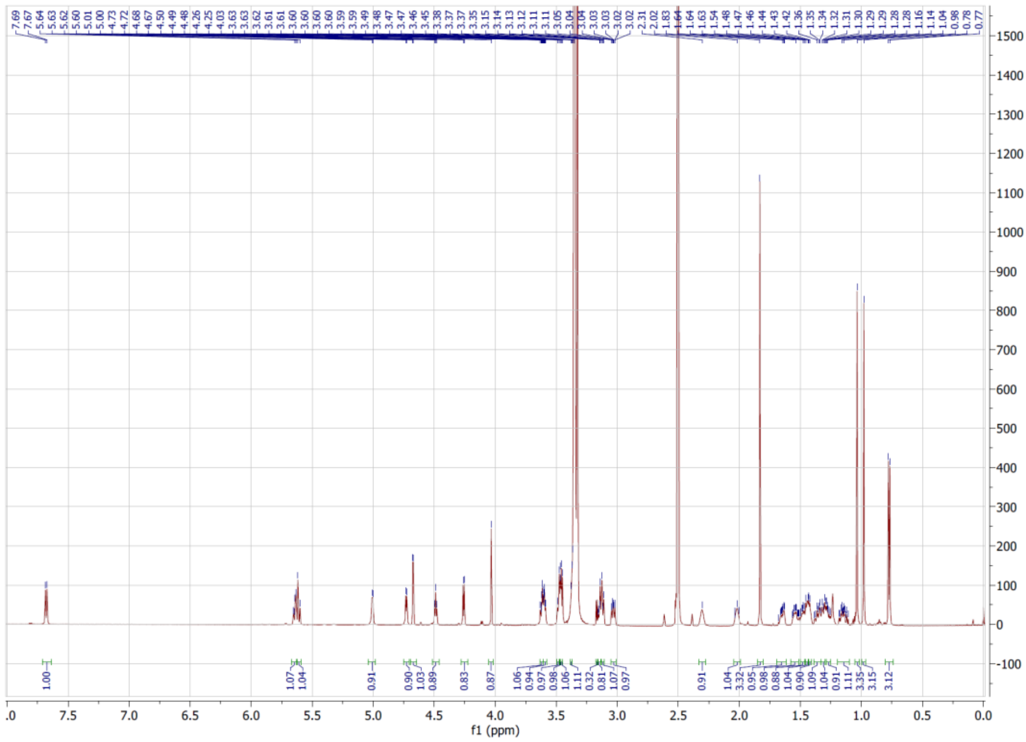

Figure S1. ^{1}H NMR (600 MHz, DMSO-d_6) spectrum of compound **2**.

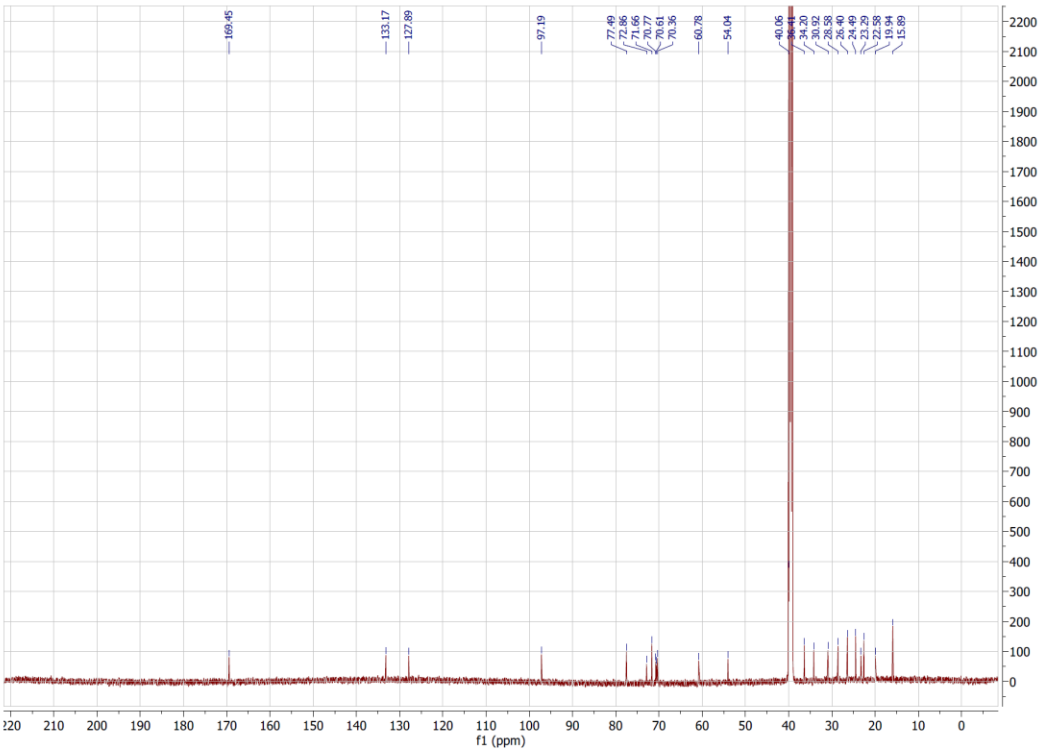

Figure S2. ^{13}C NMR (150 MHz, DMSO-d_6) spectrum of compound **2**.

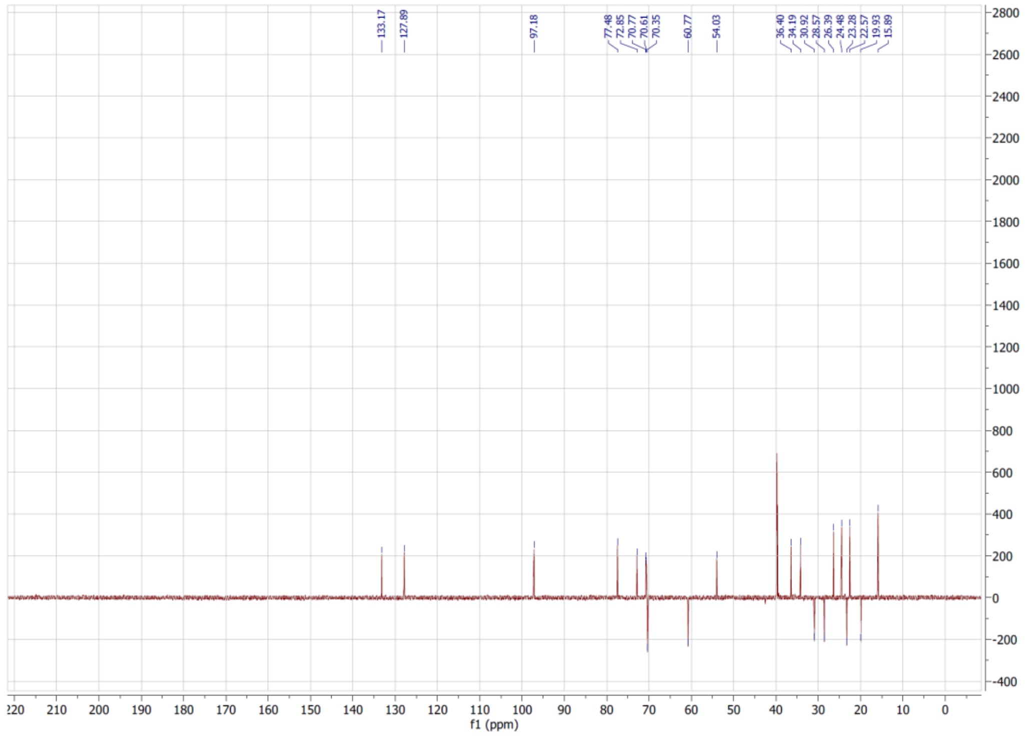

Figure S3. DEPT (150 MHz, DMSO-d_6) spectrum of compound **2**.

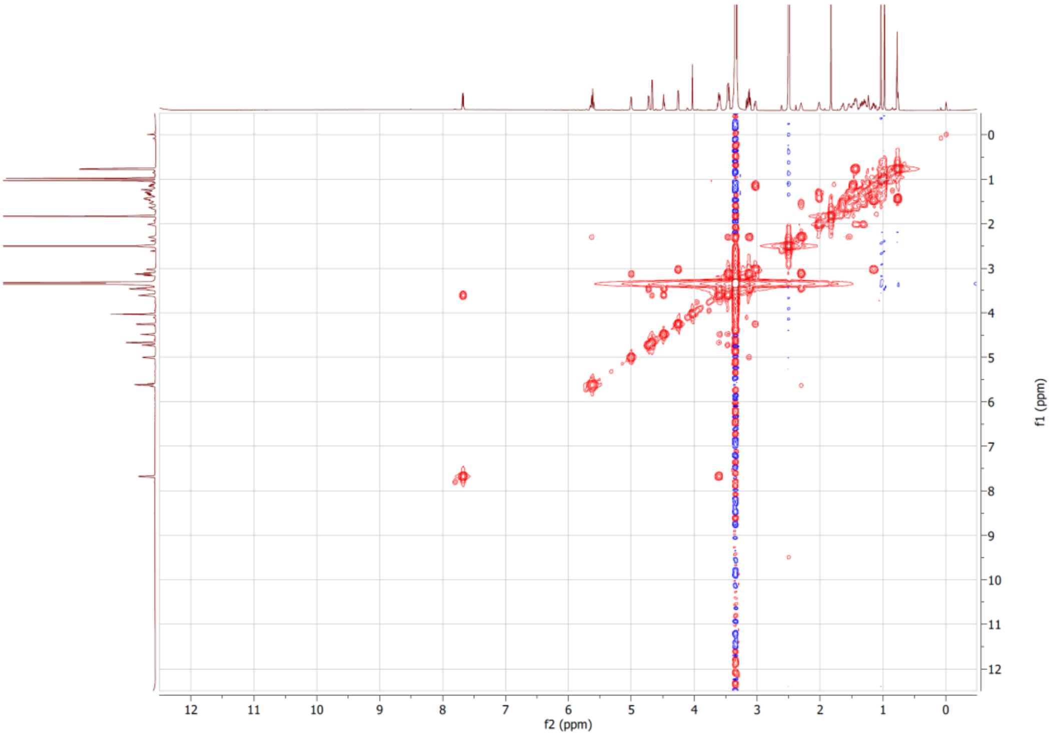

Figure S4. ^{1}H-^{1}H COSY (DMSO-d_6) spectrum of compound **2**.

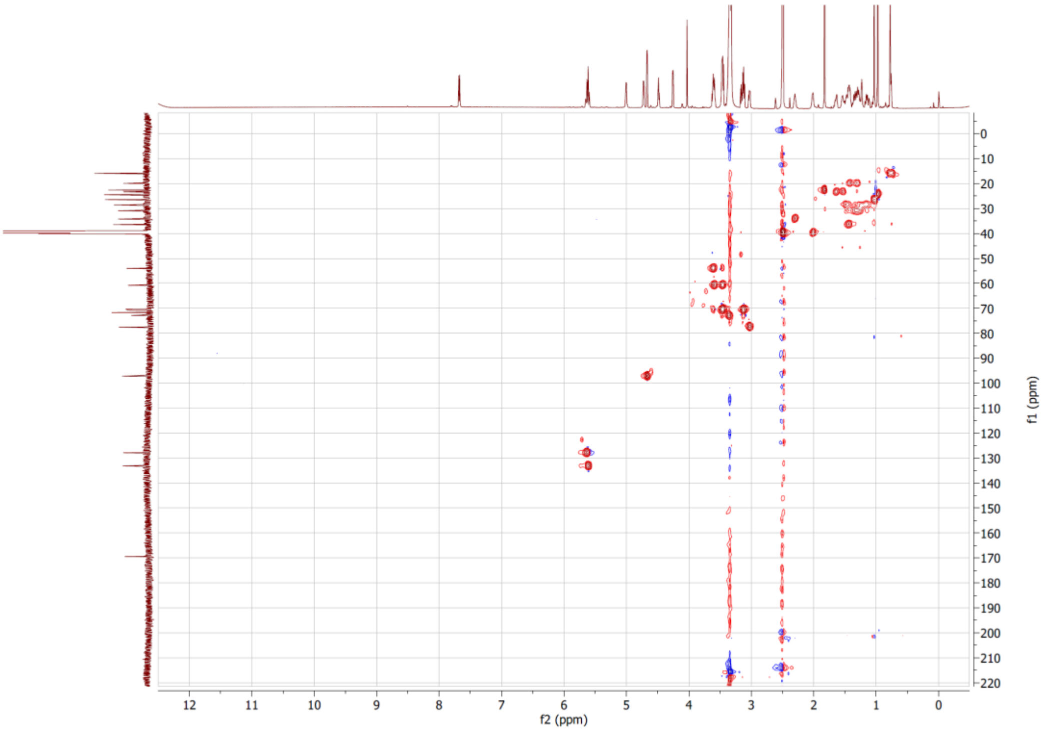

Figure S5. HSQC (DMSO-d_6) spectrum of compound **2**.

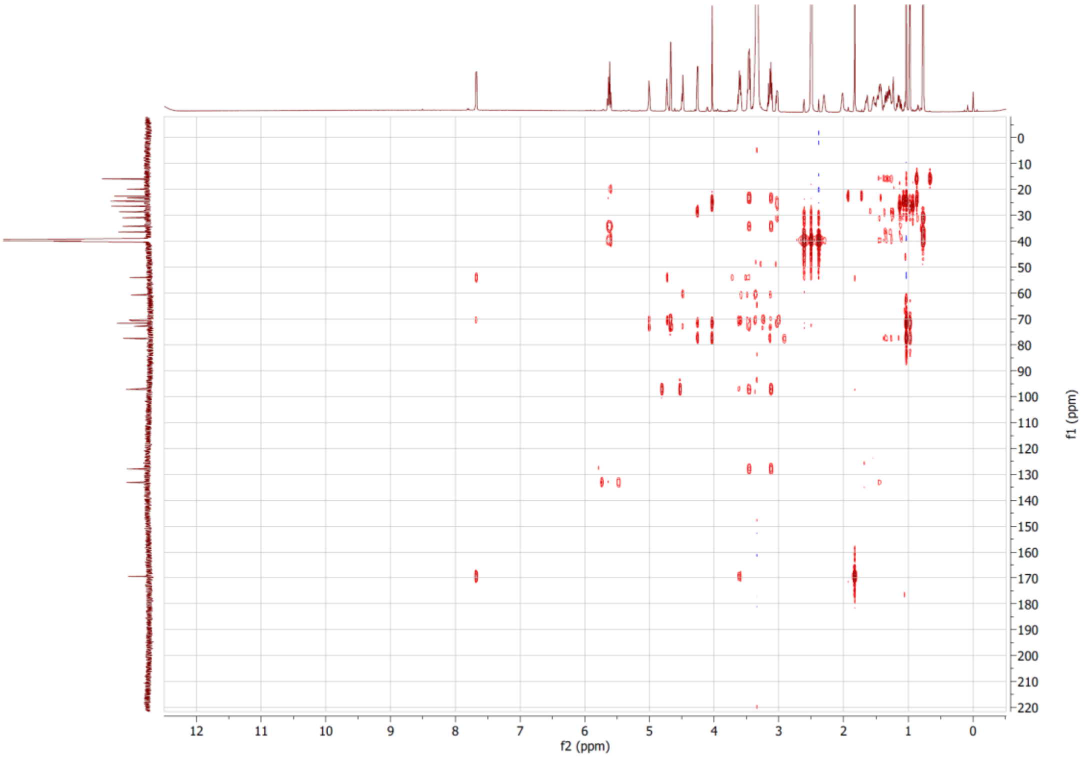

Figure S6. HMBC (DMSO-d_6) spectrum of compound **2**.

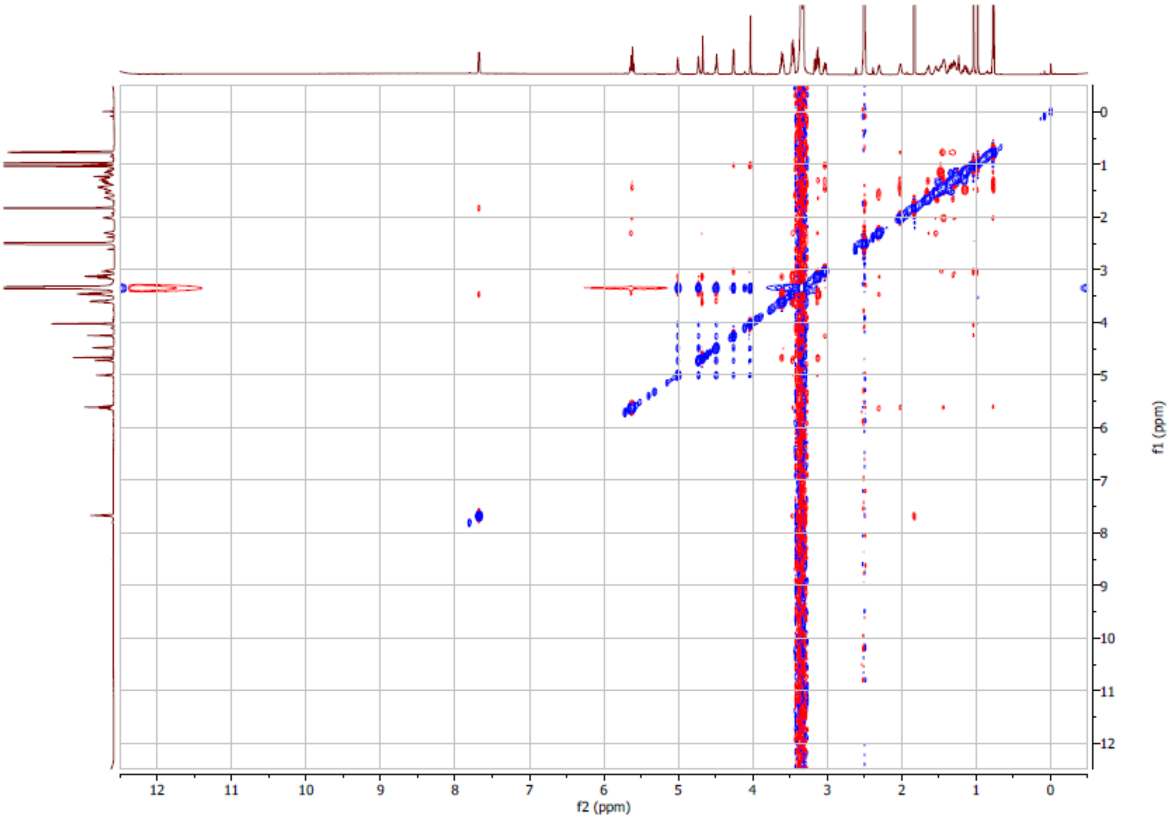

Figure S7. ROESY (DMSO-d_6) spectrum of compound **2**.

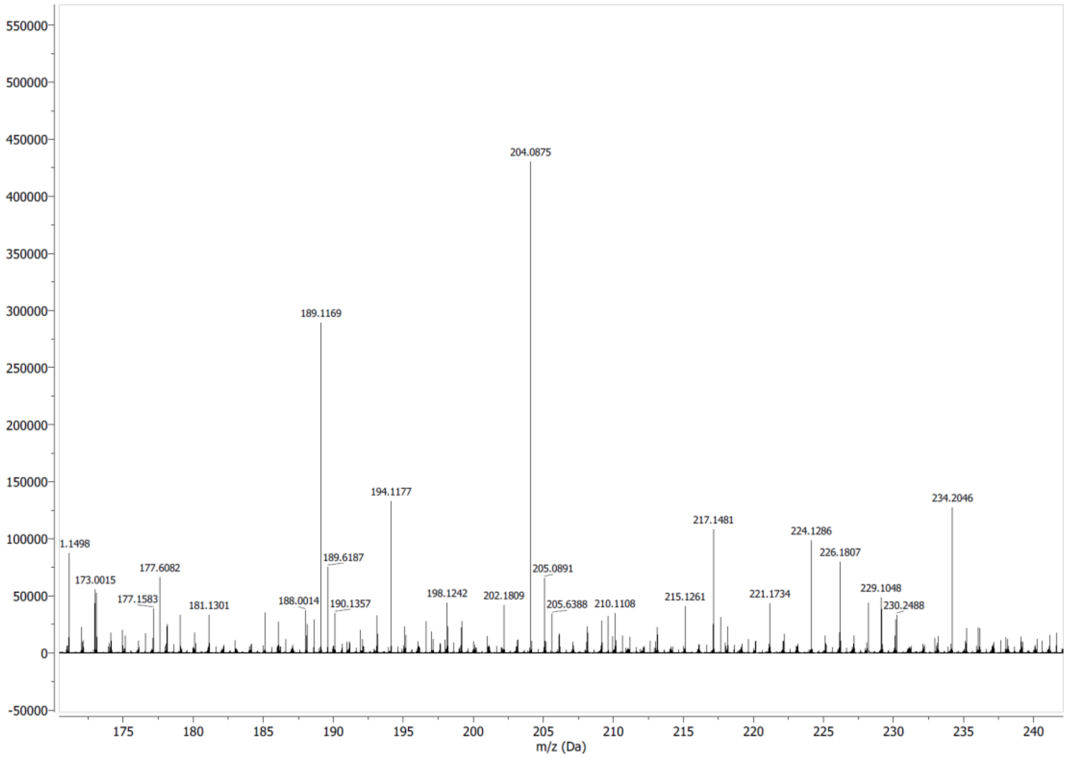

Figure S8. Partial HRESIMS spectrum of compound **2**.

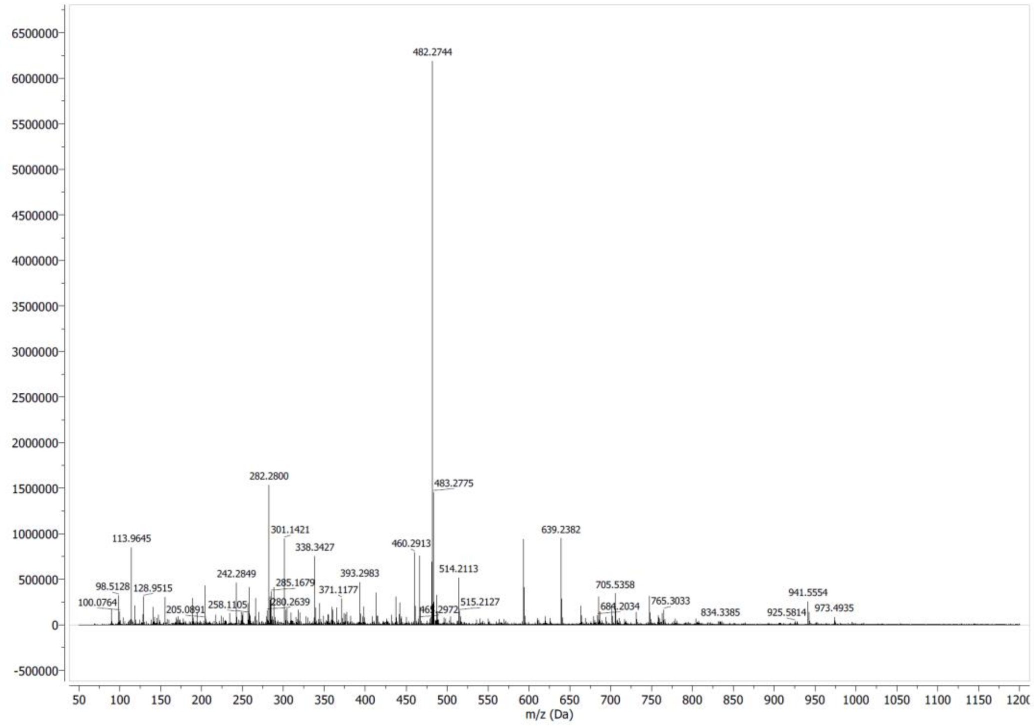

Figure S9. HRESIMS spectrum of compound **2**.

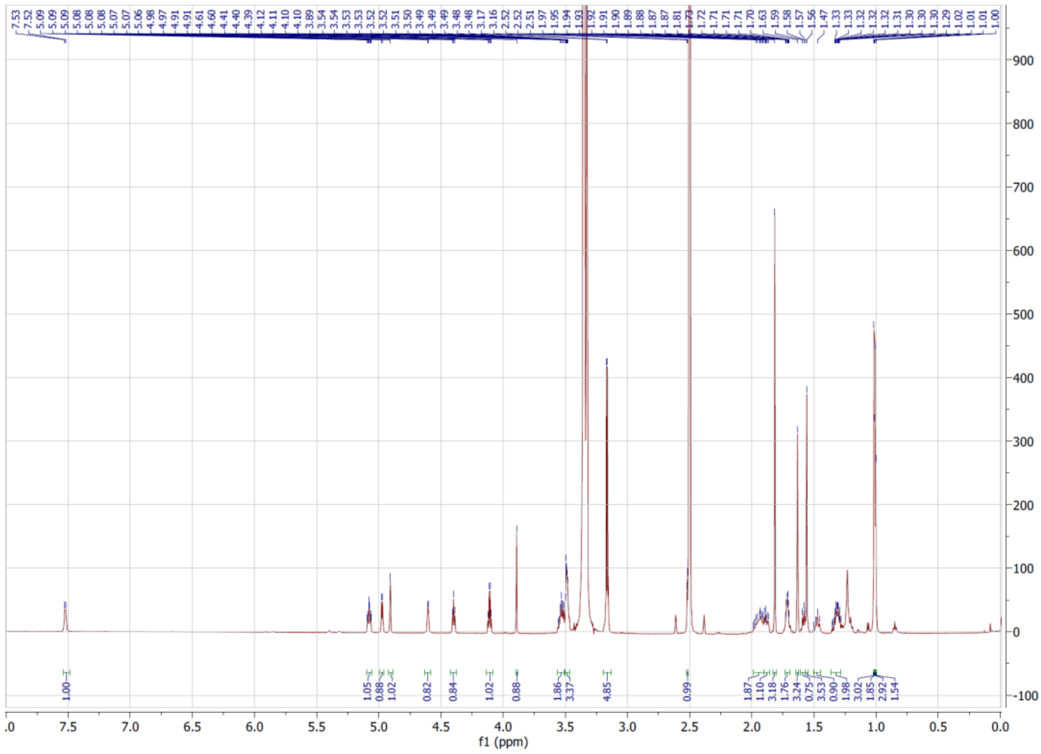

Figure S10. ^{1}H NMR (600 MHz, DMSO-d_6) spectrum of compound **5**.

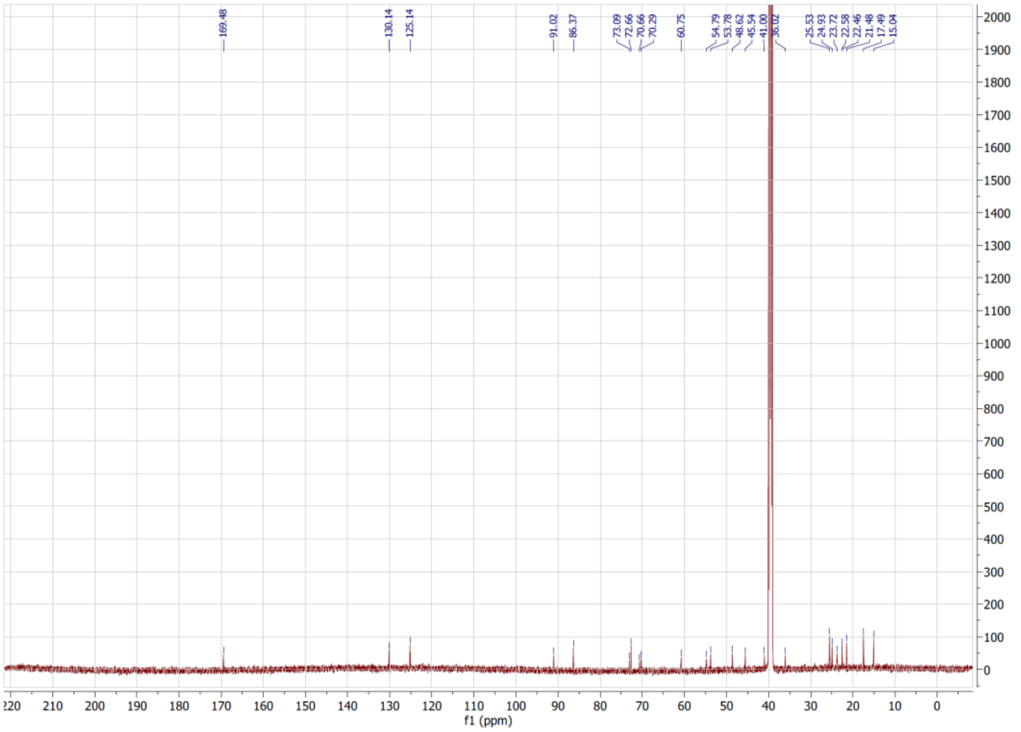

Figure S11. ^{13}C NMR (150 MHz, DMSO-d_6) spectrum of compound **5**.

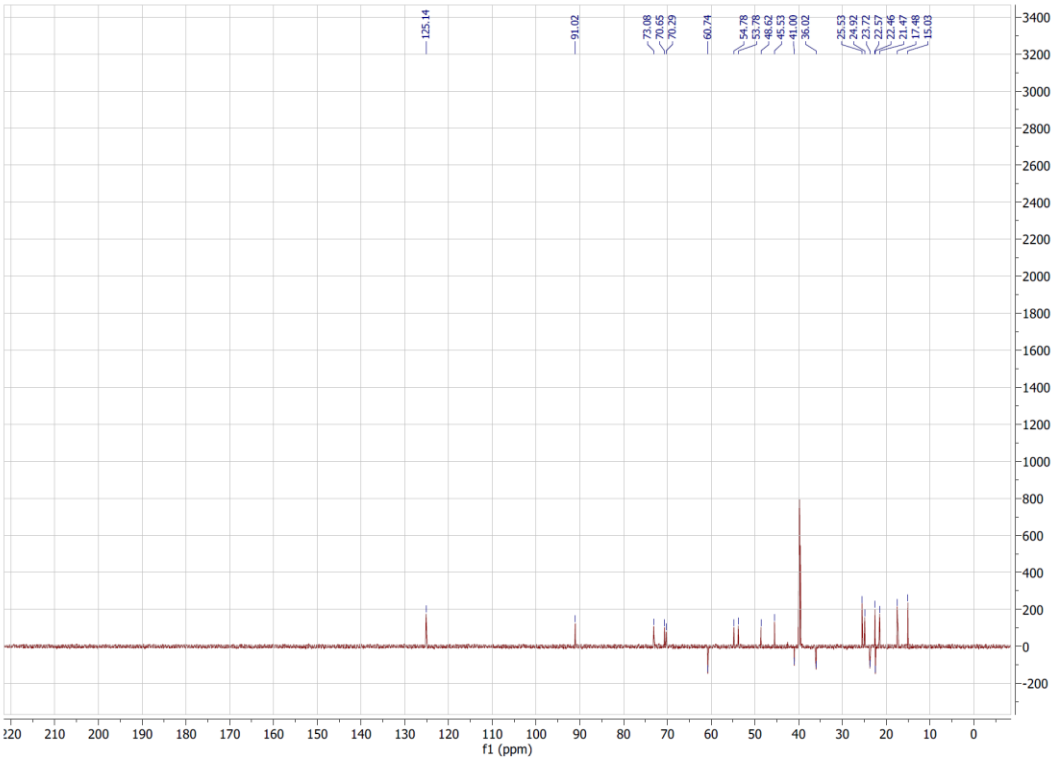

Figure S12. DEPT (150 MHz, DMSO-d_6) spectrum of compound **5**.

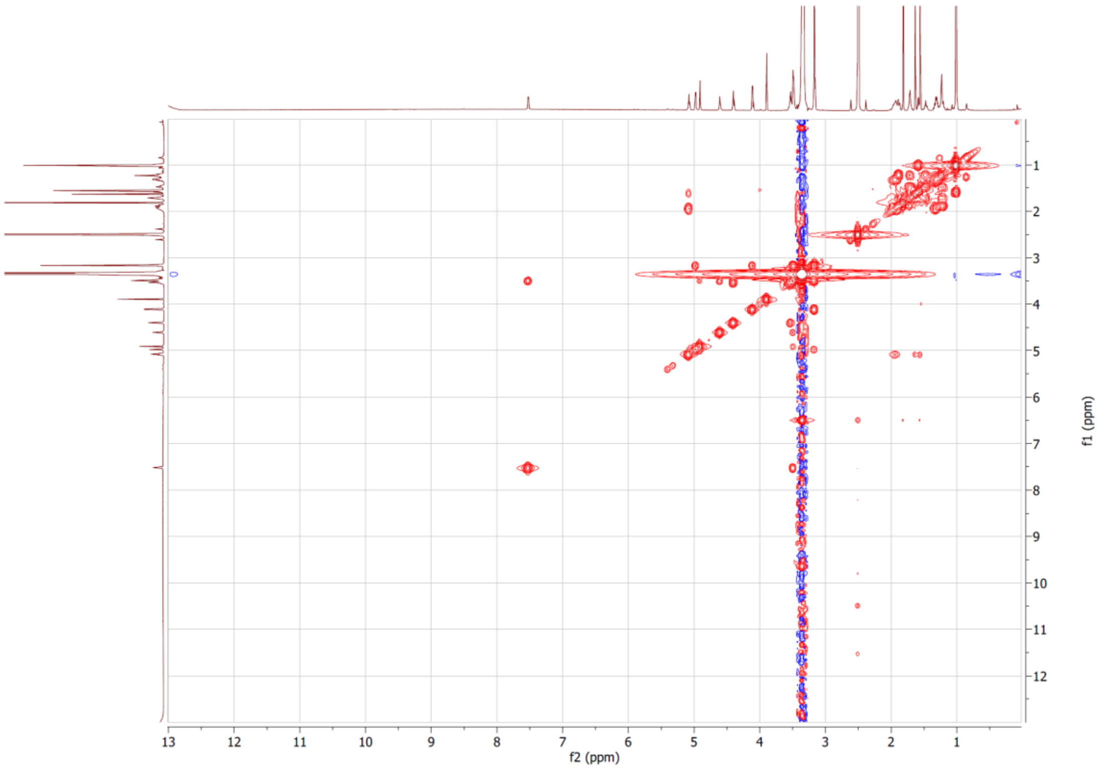

Figure S13. ^{1}H-^{1}H COSY (DMSO-d_6) spectrum of compound **5**.

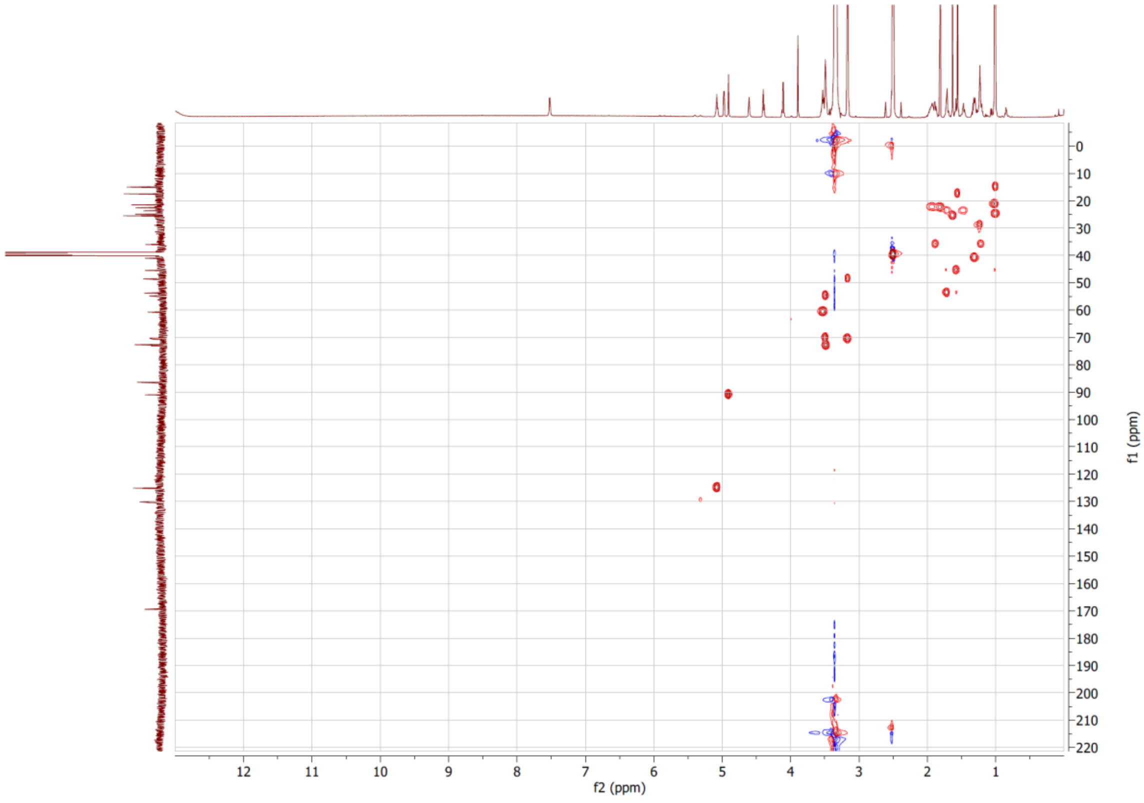

Figure S14. HSQC (DMSO-d_6) spectrum of compound **5**.

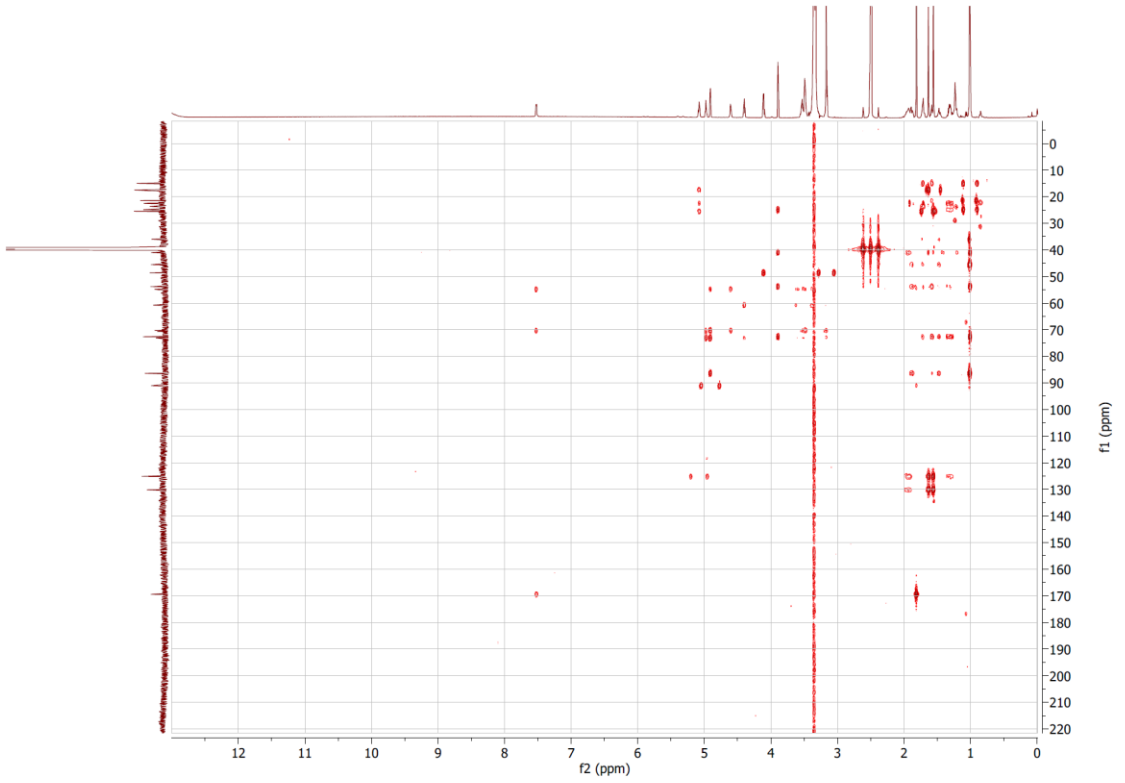

Figure S15. HMBC (DMSO-d_6) spectrum of compound **5**.

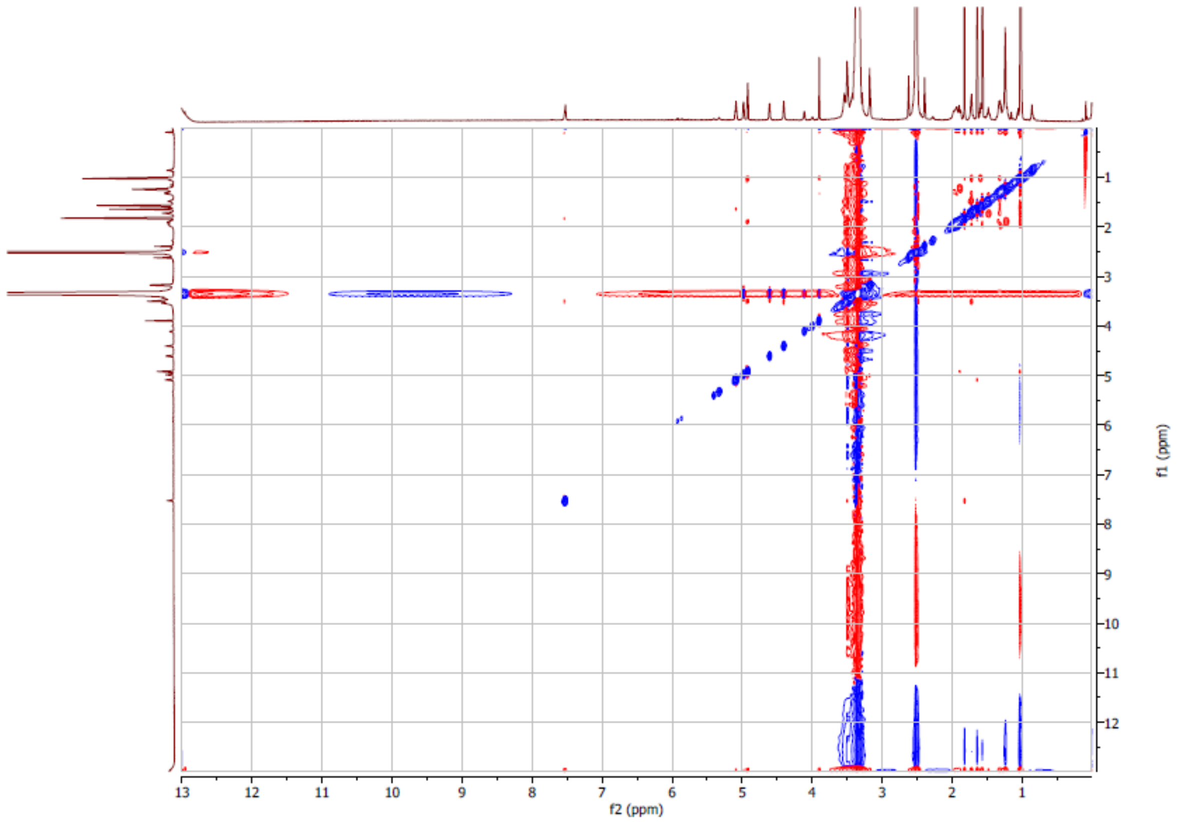

Figure S16. ROESY (DMSO-d_6) spectrum of compound **5**.

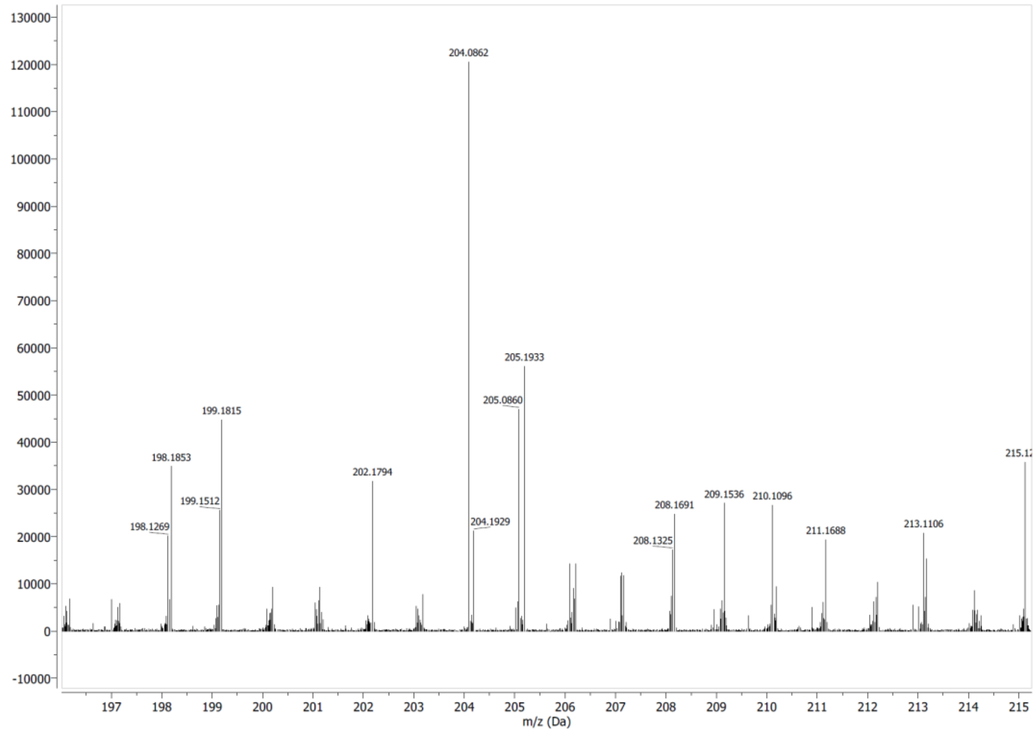

Figure S17. Partial HRESIMS spectrum of compound **5**.

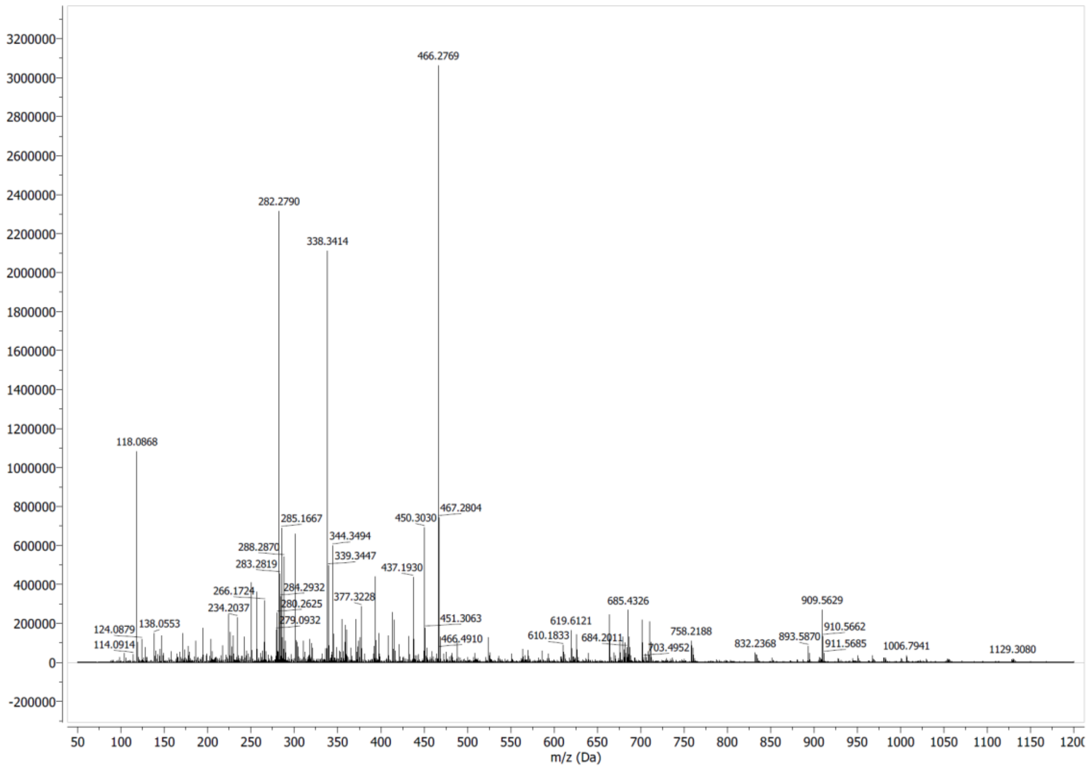

Figure S18. HRESIMS spectrum of compound **5**.

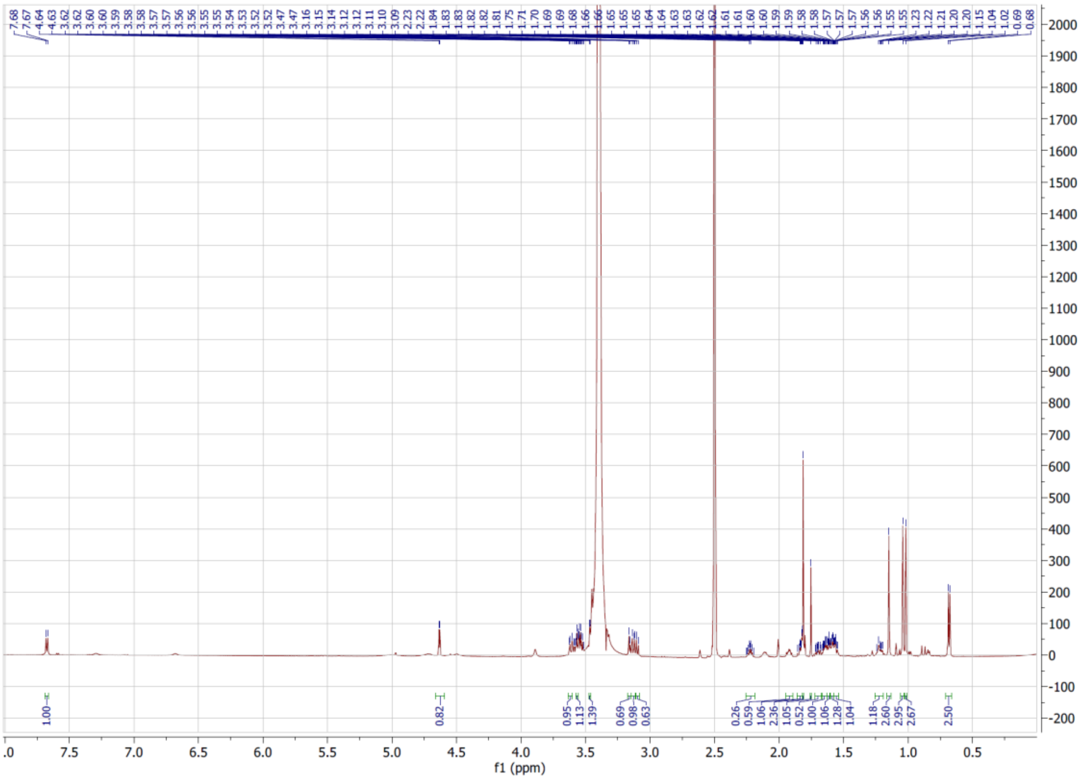

Figure S19. ^{1}H NMR (600 MHz, DMSO-d_6) spectrum of compound **6**.

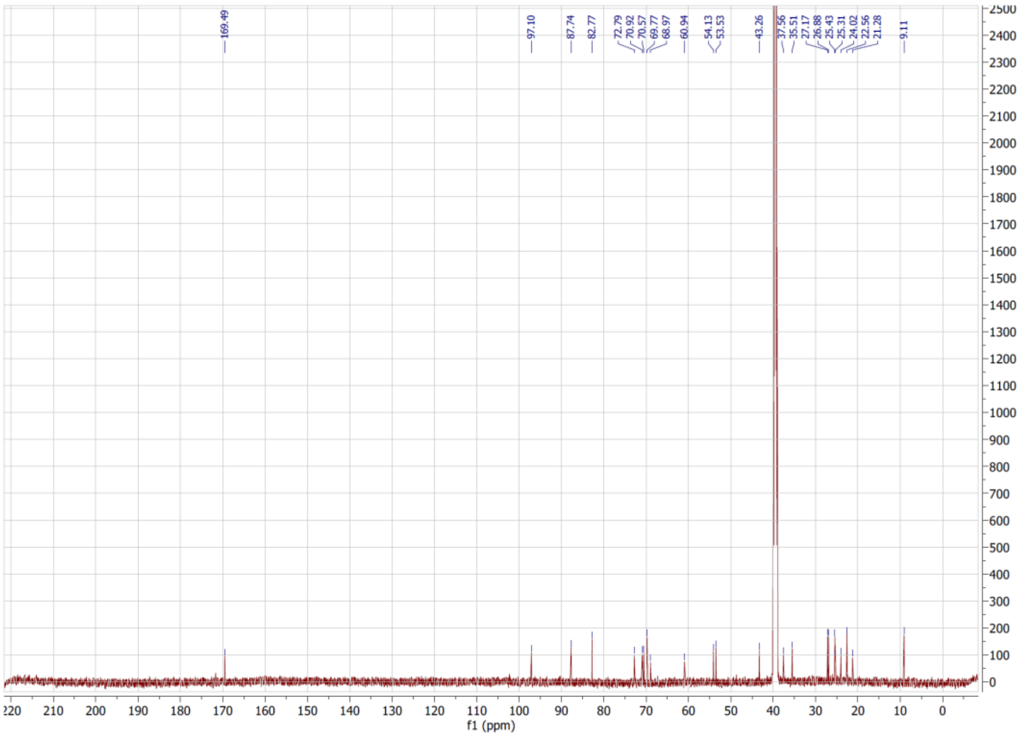

Figure S20. ^{13}C NMR (150 MHz, DMSO-d_6) spectrum of compound **6**.

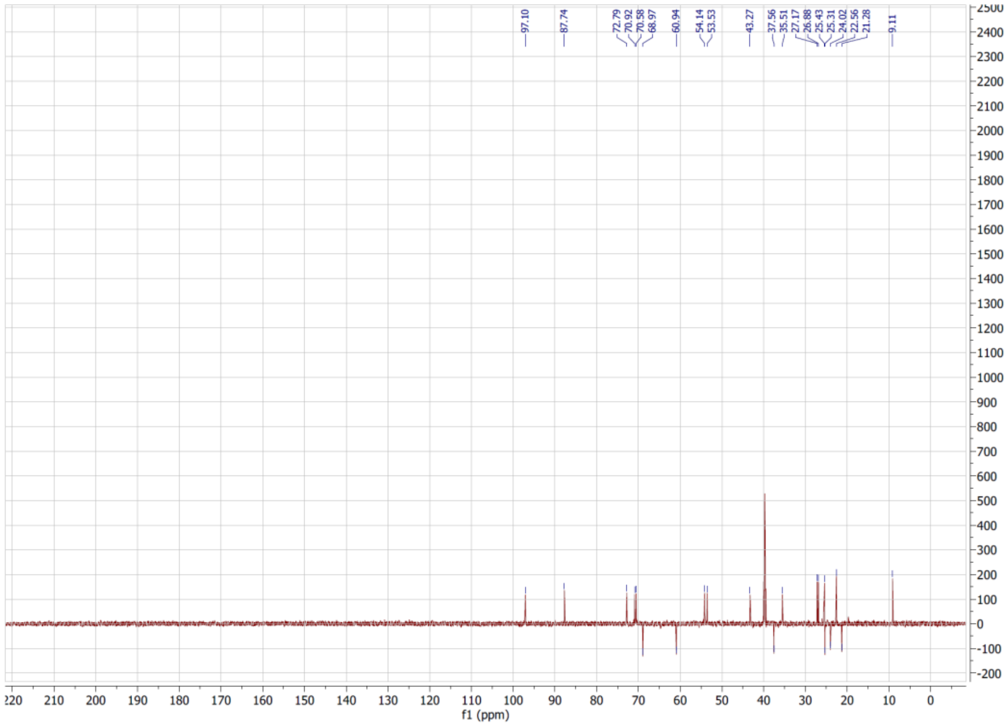

Figure S21. DEPT (150 MHz, DMSO-d_6) spectrum of compound **6**.

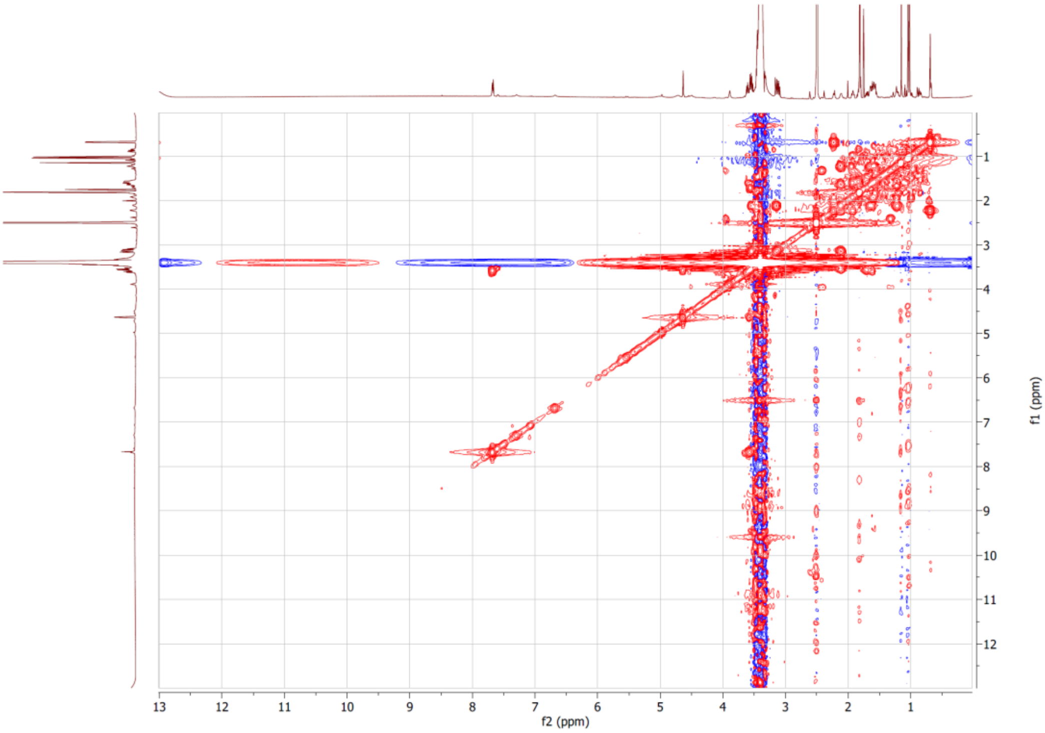

Figure S22. ^{1}H-^{1}H COSY (DMSO-d_6) spectrum of compound **6**.

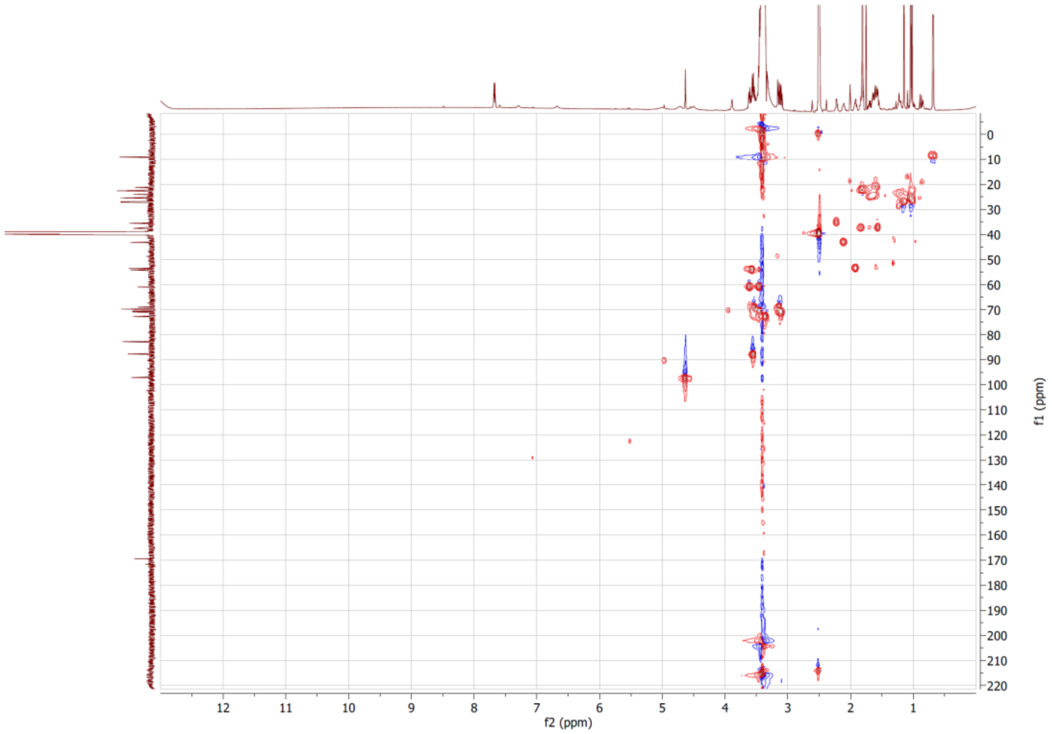

Figure S23. HSQC (DMSO-d_6) spectrum of compound **6**.

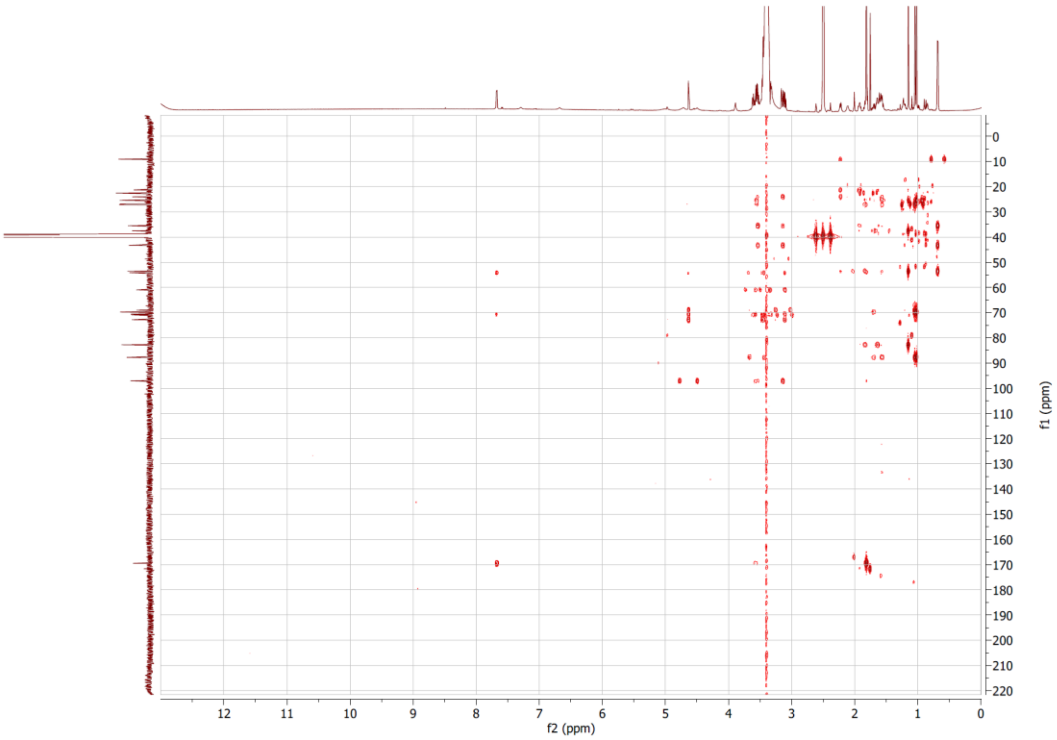

Figure S24. HMBC (DMSO-d_6) spectrum of compound **6**.

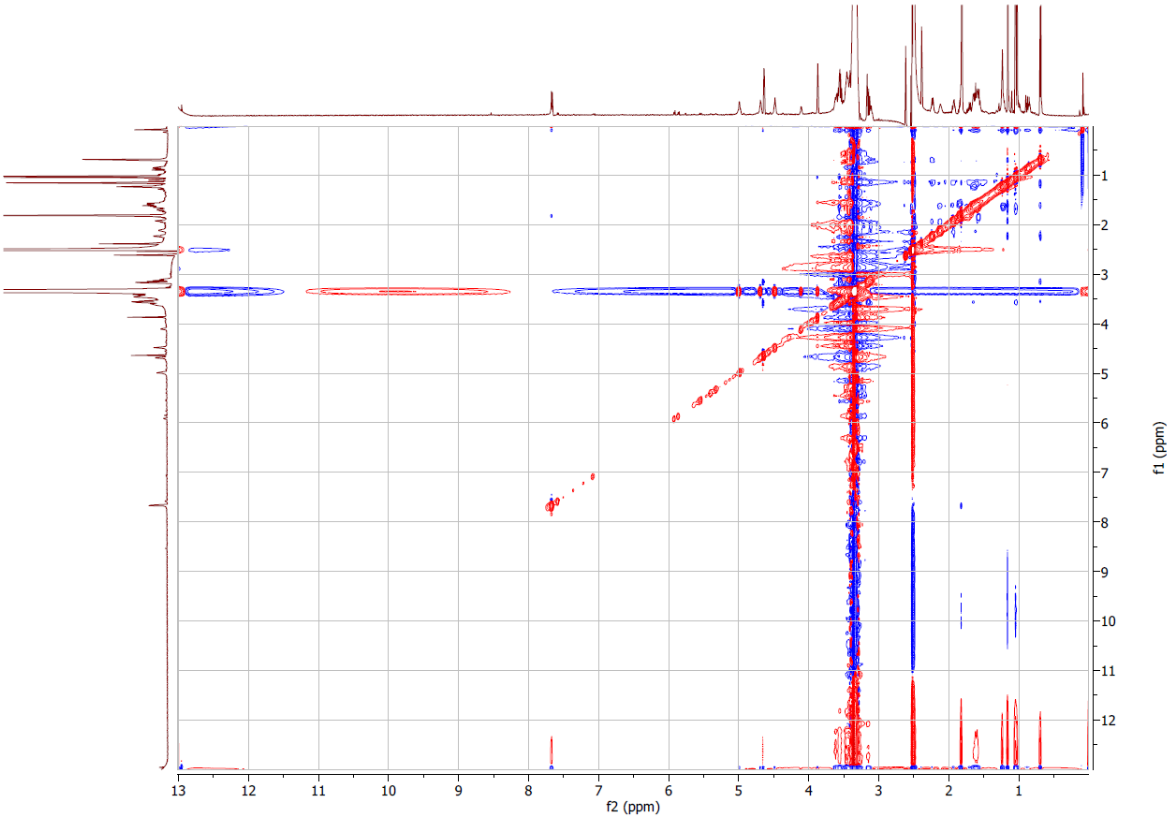

Figure S25. ROESY (DMSO-d_6) spectrum of compound **6**.

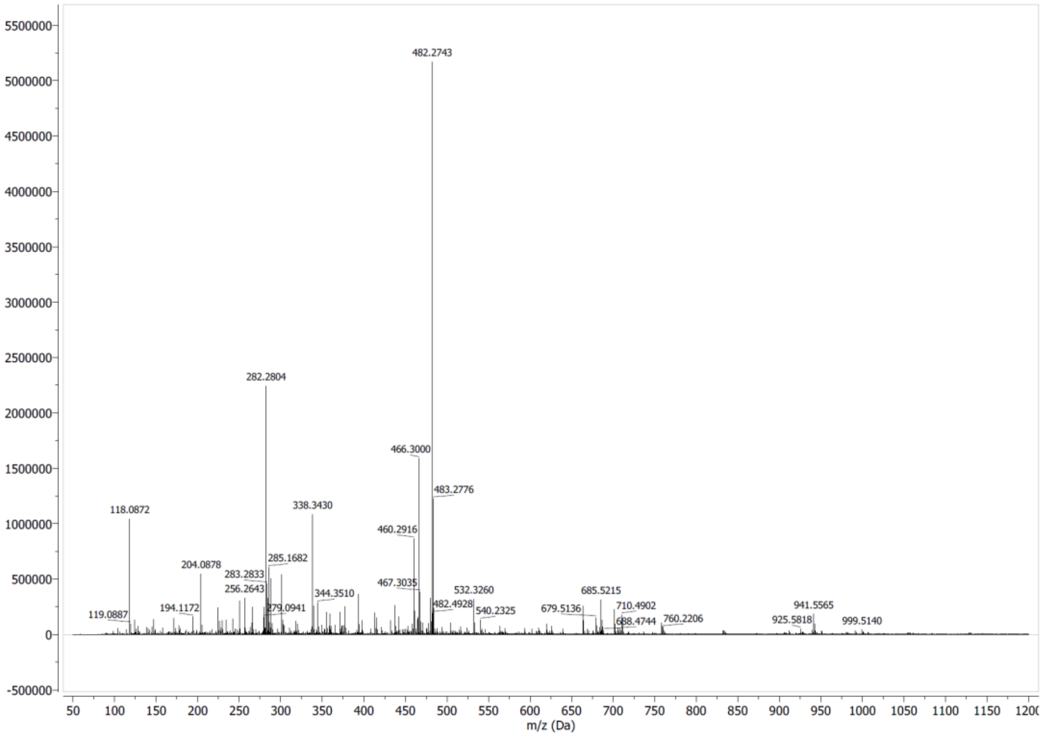

Figure S26. HRESIMS spectrum of compound **6**.

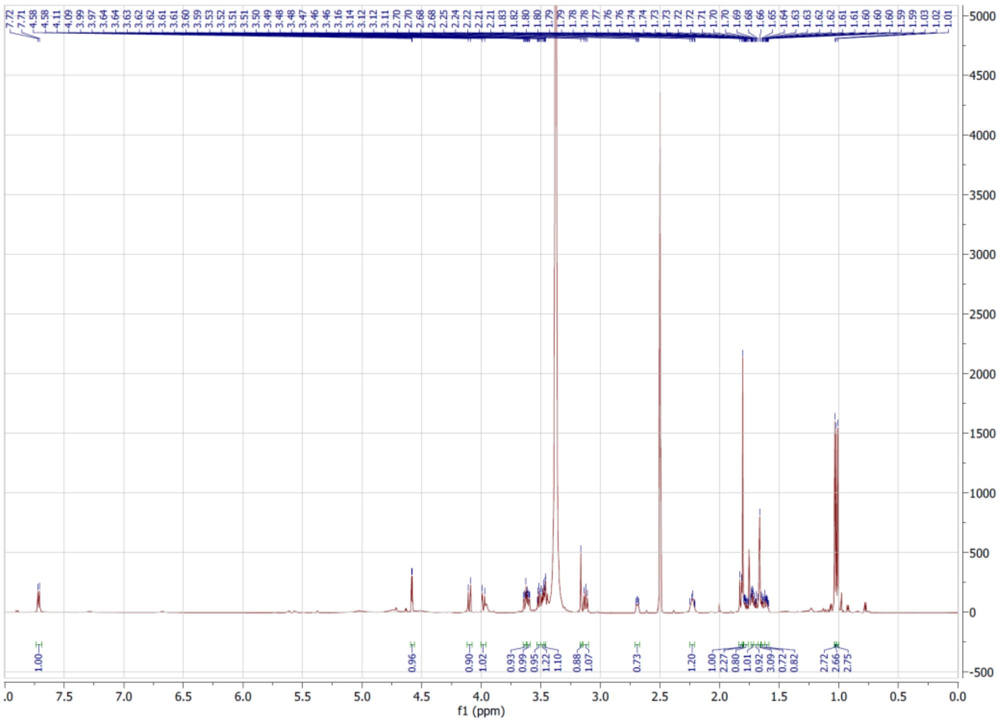

Figure S27. ^{1}H NMR (600 MHz, DMSO-d_6) spectrum of compound **7**.

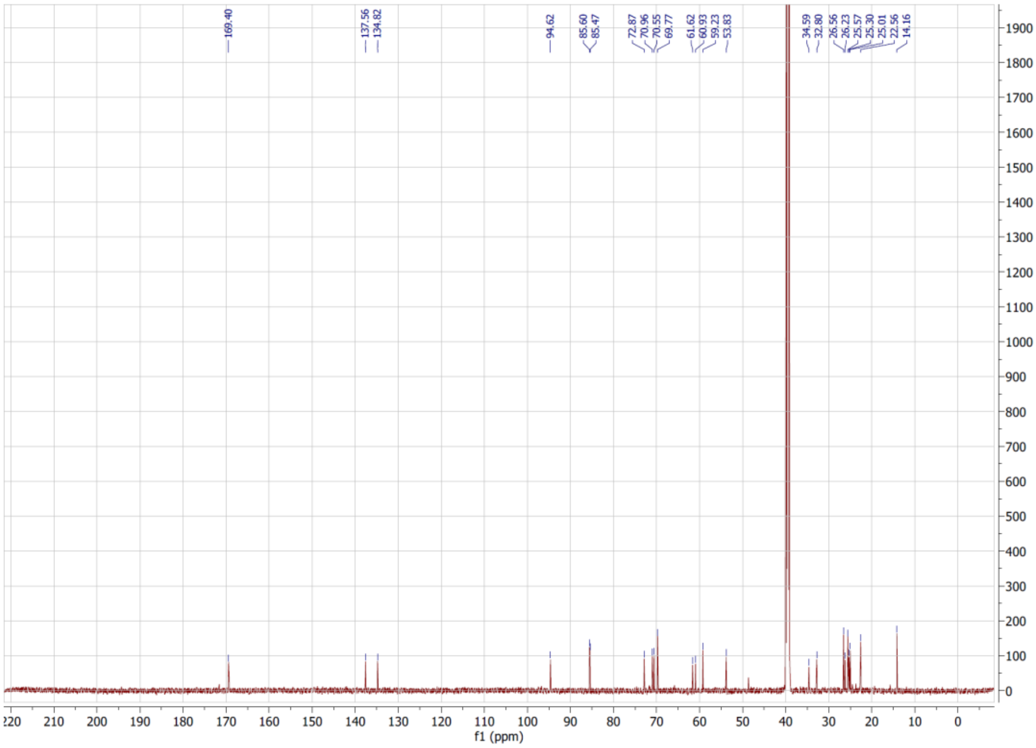

Figure S28. ^{13}C NMR (150 MHz, DMSO-d_6) spectrum of compound **7**.

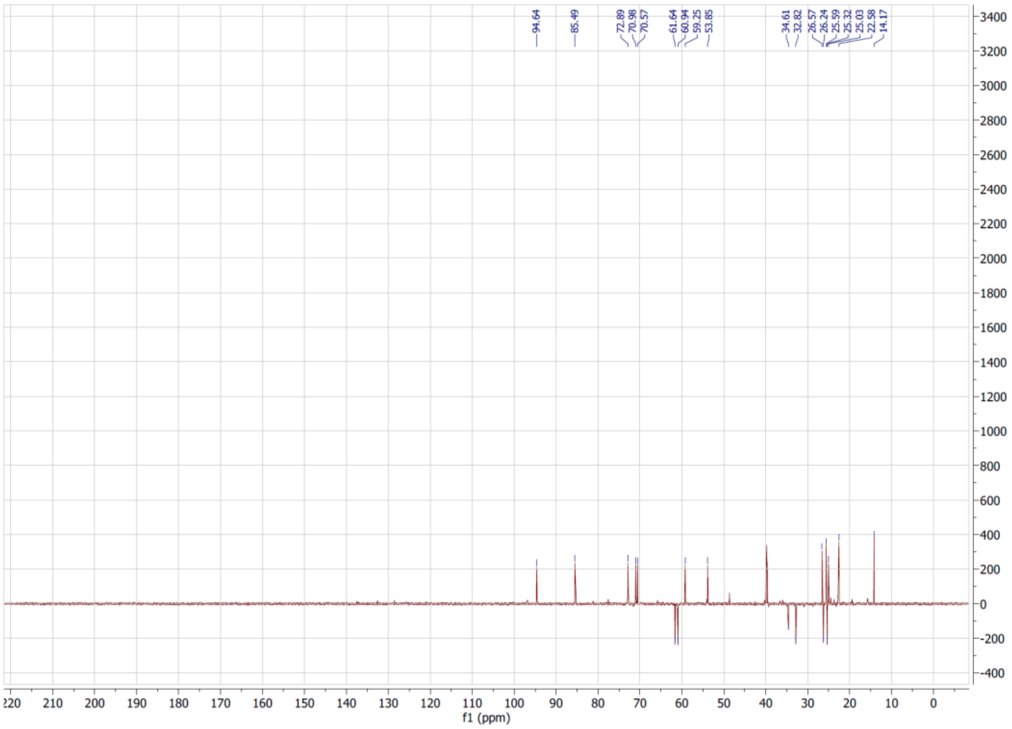

Figure S29. DEPT (150 MHz, DMSO-d_6) spectrum of compound **7**.

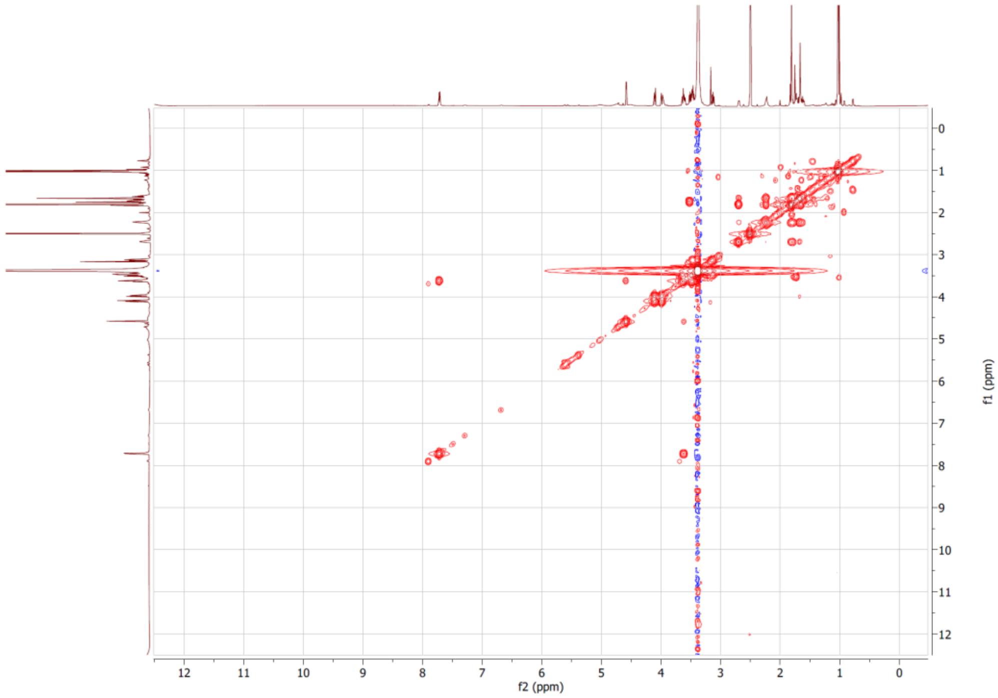

Figure S30. ^{1}H-^{1}H COSY (DMSO-d_6) spectrum of compound **7**.

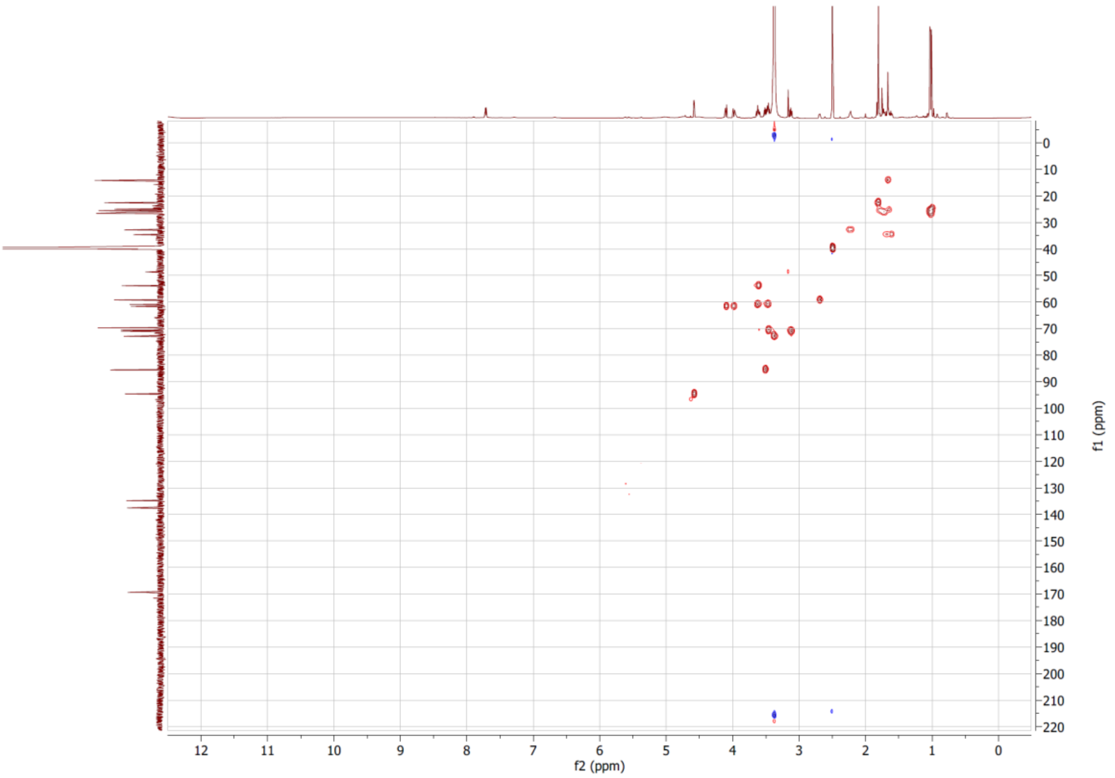

Figure S31. HSQC (DMSO-d_6) spectrum of compound **7**.

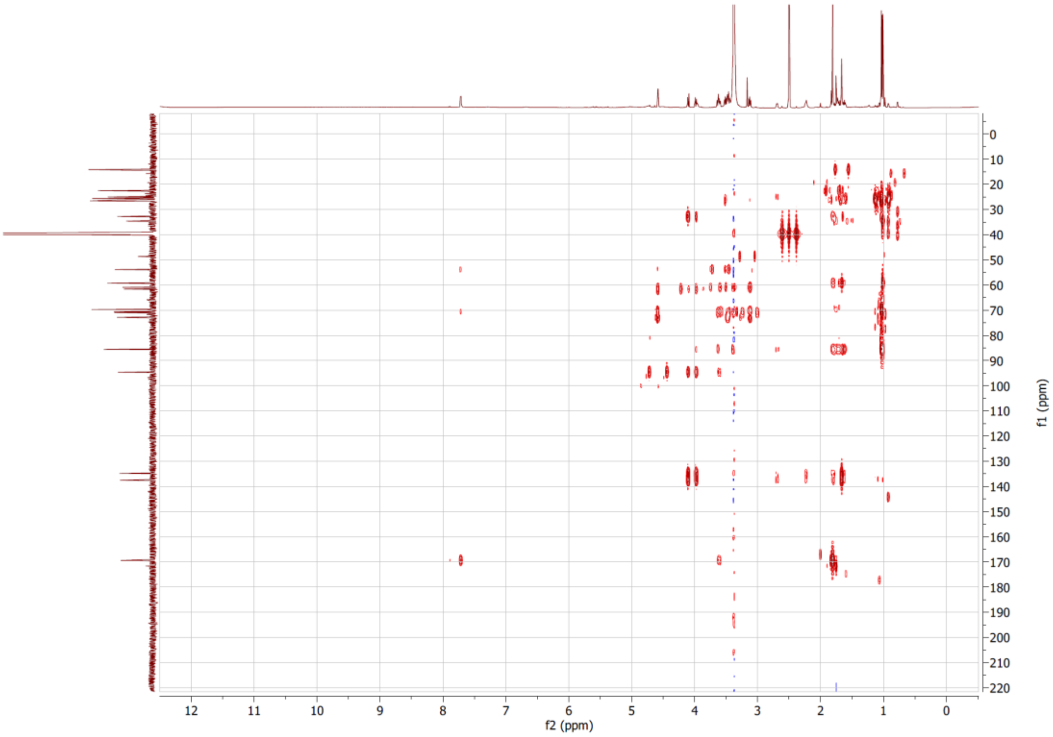

Figure S32. HMBC (DMSO-d_6) spectrum of compound **7**.

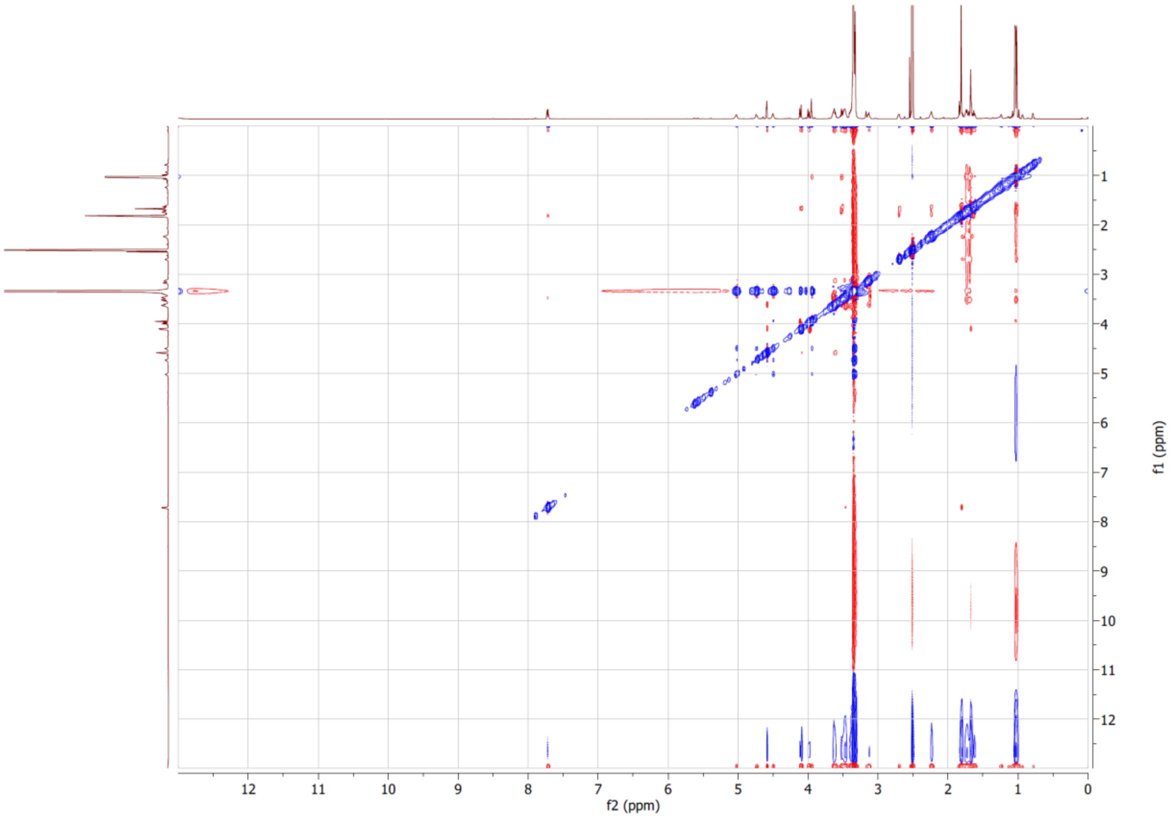

Figure S33. ROESY (DMSO-d_6) spectrum of compound **7**.

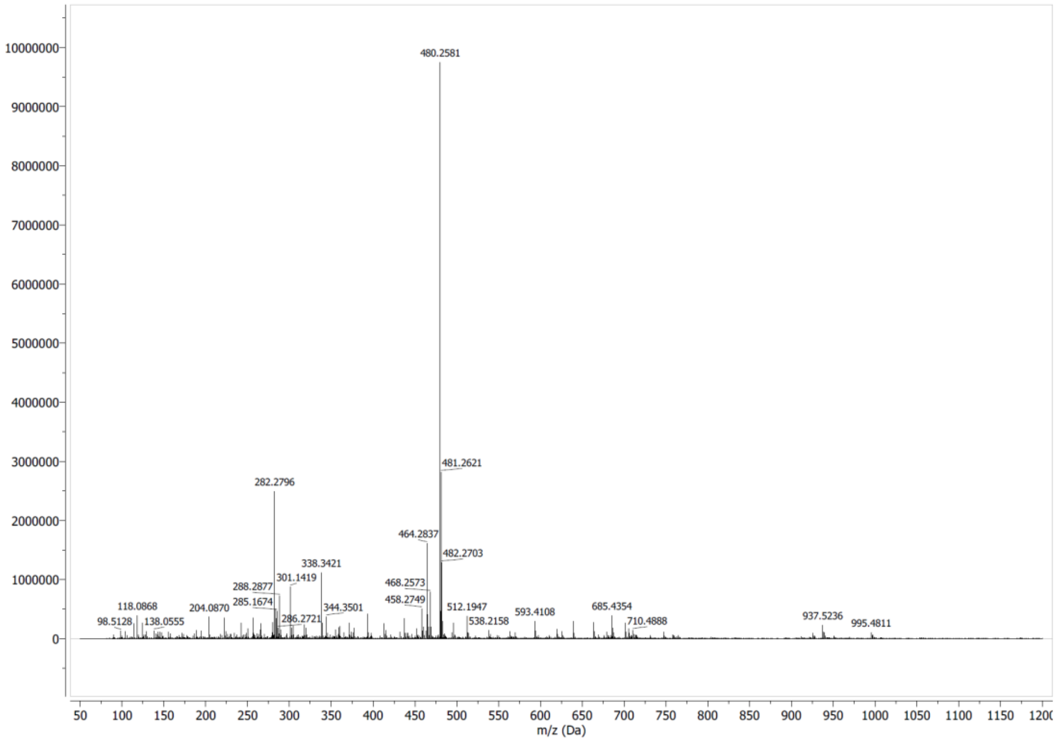

Figure S34. HRESIMS spectrum of compound **7**.

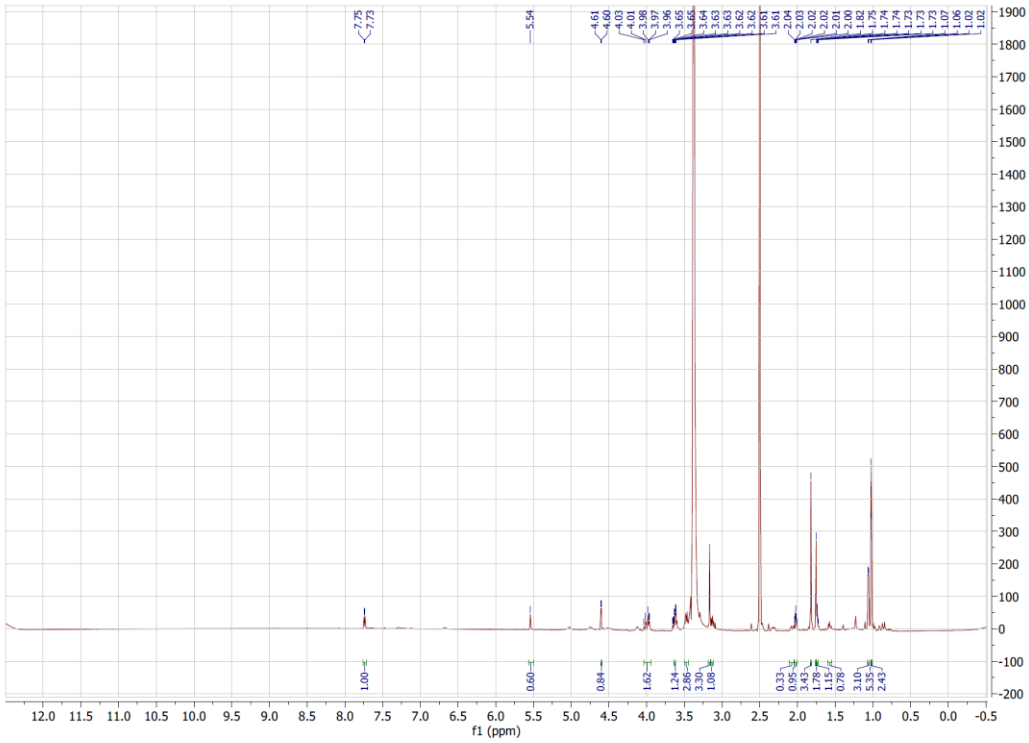

Figure S35. ^{1}H NMR (600 MHz, DMSO-d_6) spectrum of compound **8**.

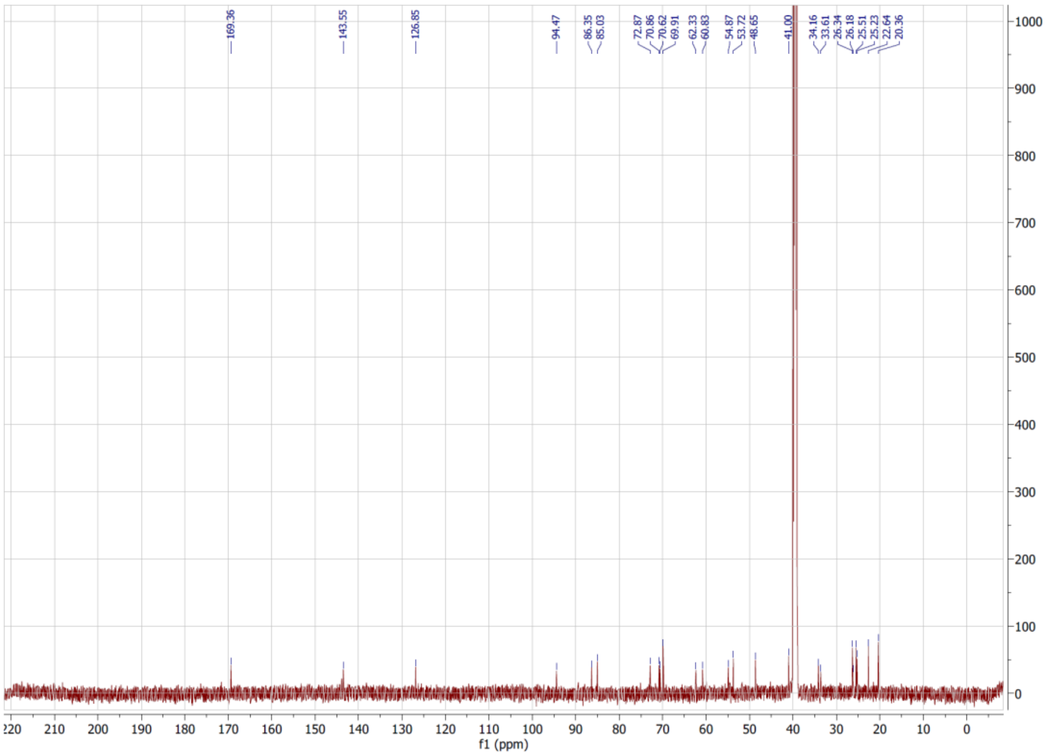

Figure S36. ^{13}C NMR (150 MHz, DMSO-d_6) spectrum of compound **8**.

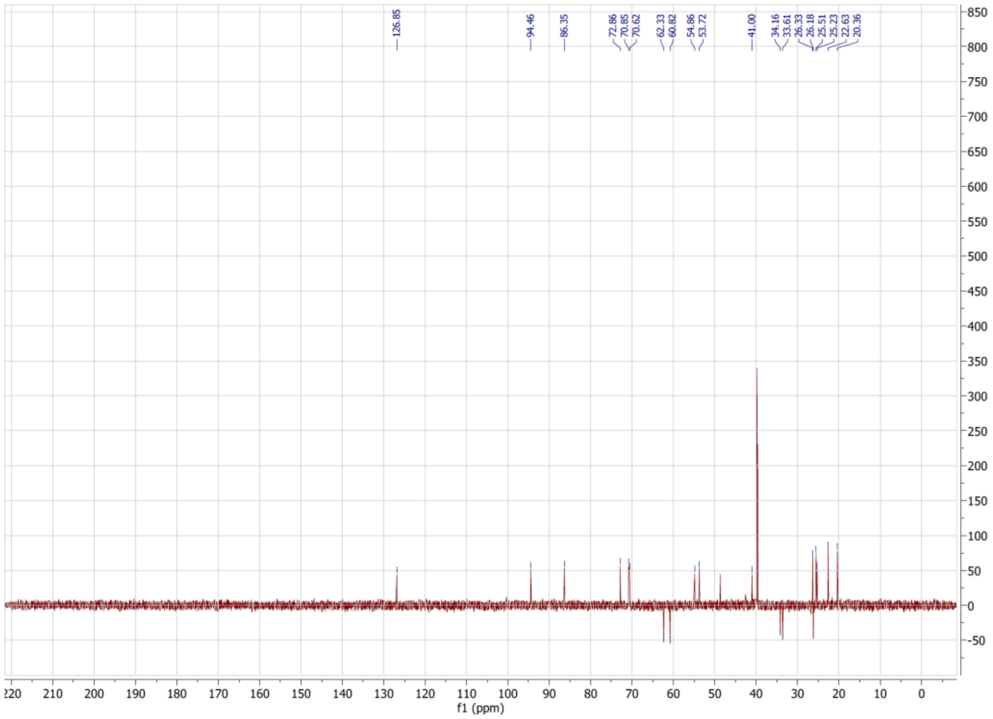

Figure S37. DEPT (150 MHz, DMSO-d_6) spectrum of compound **8**.

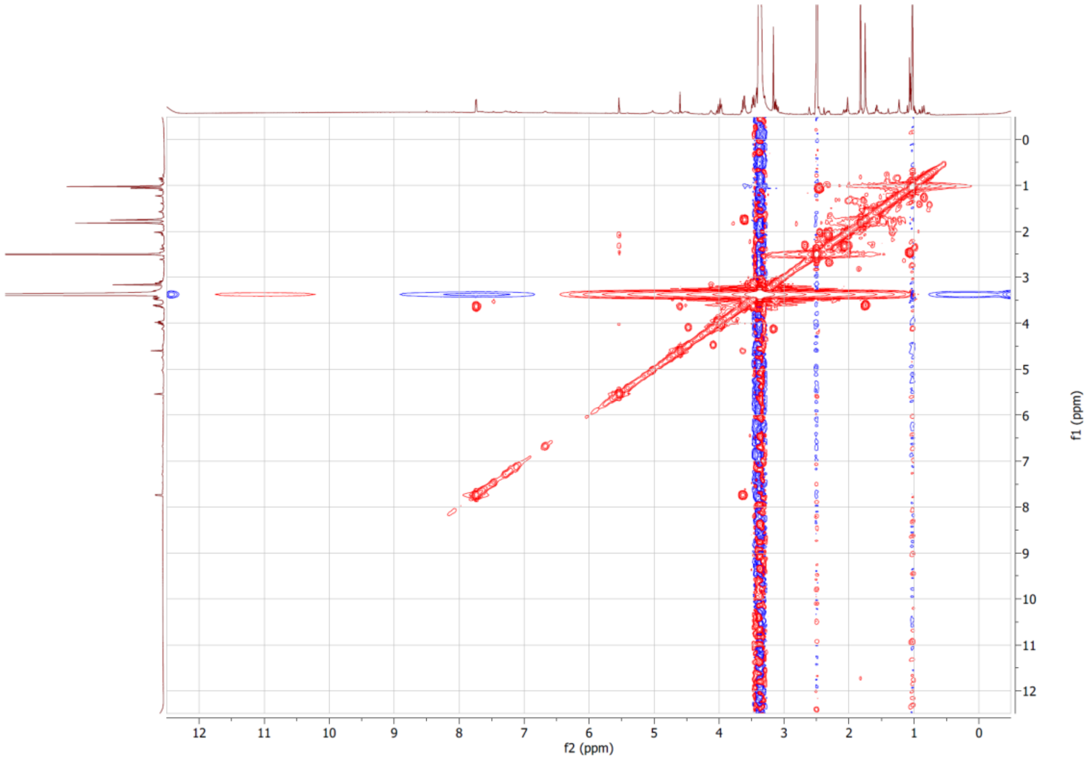

Figure S38. ^{1}H-^{1}H COSY (DMSO-d_6) spectrum of compound **8**.

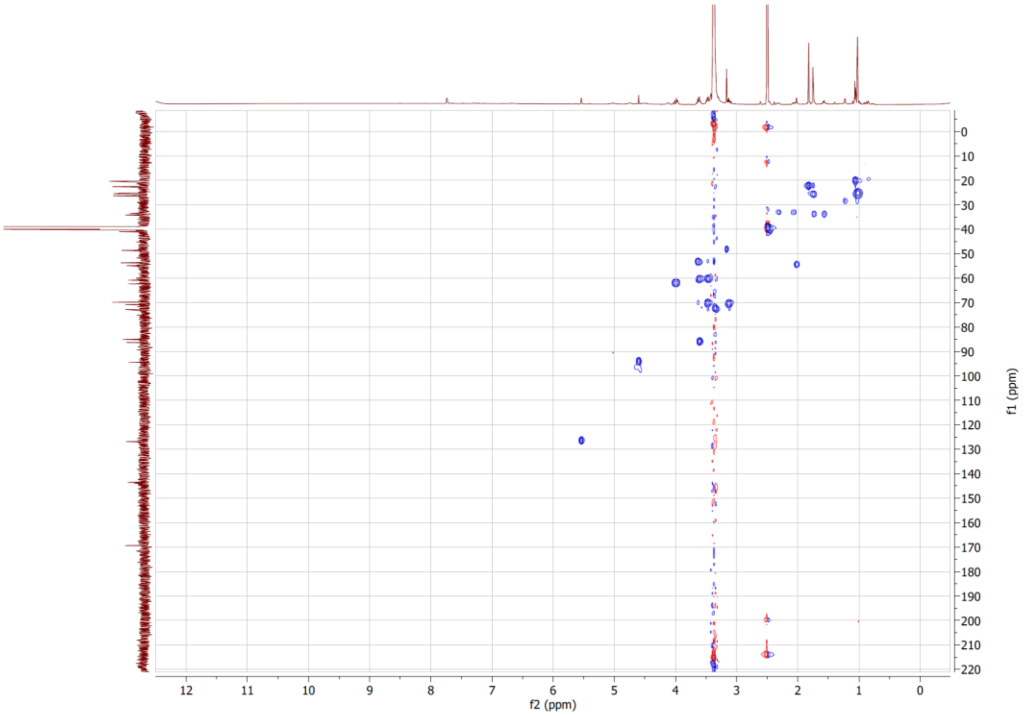

Figure S39. HSQC (DMSO-d_6) spectrum of compound **8**.

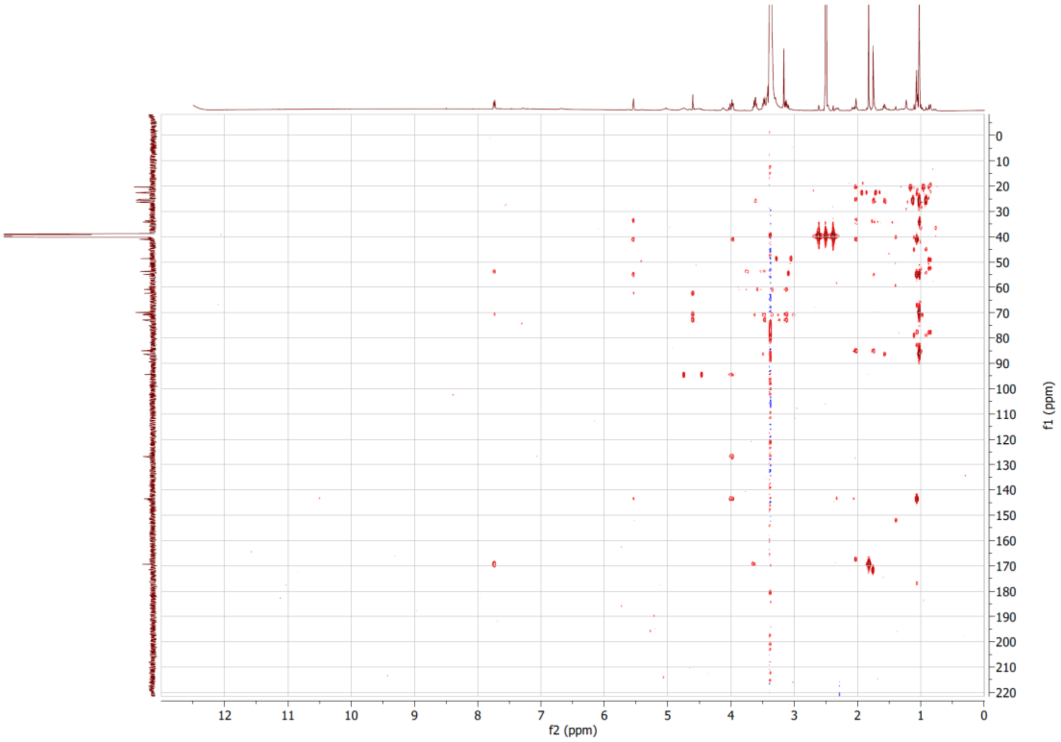

Figure S40. HMBC (DMSO-d_6) spectrum of compound **8**.

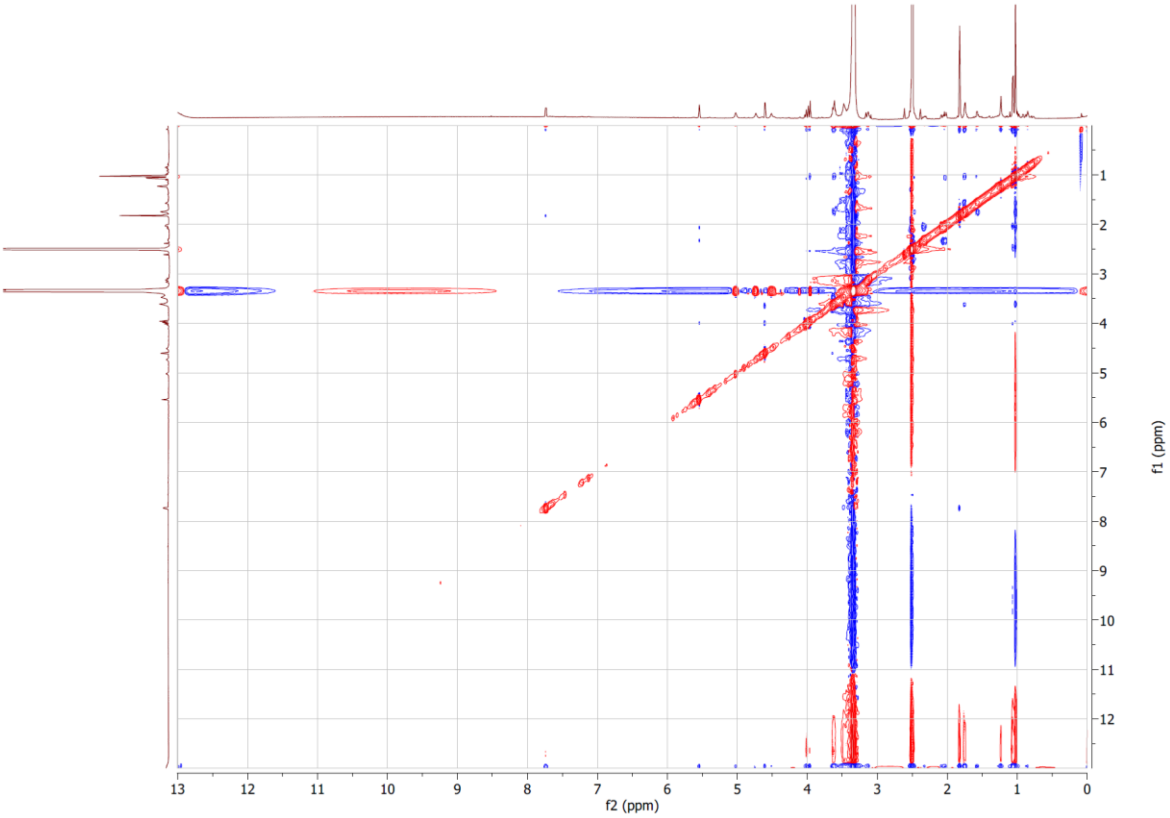

Figure S41. ROESY (DMSO-d_6) spectrum of compound **8**.

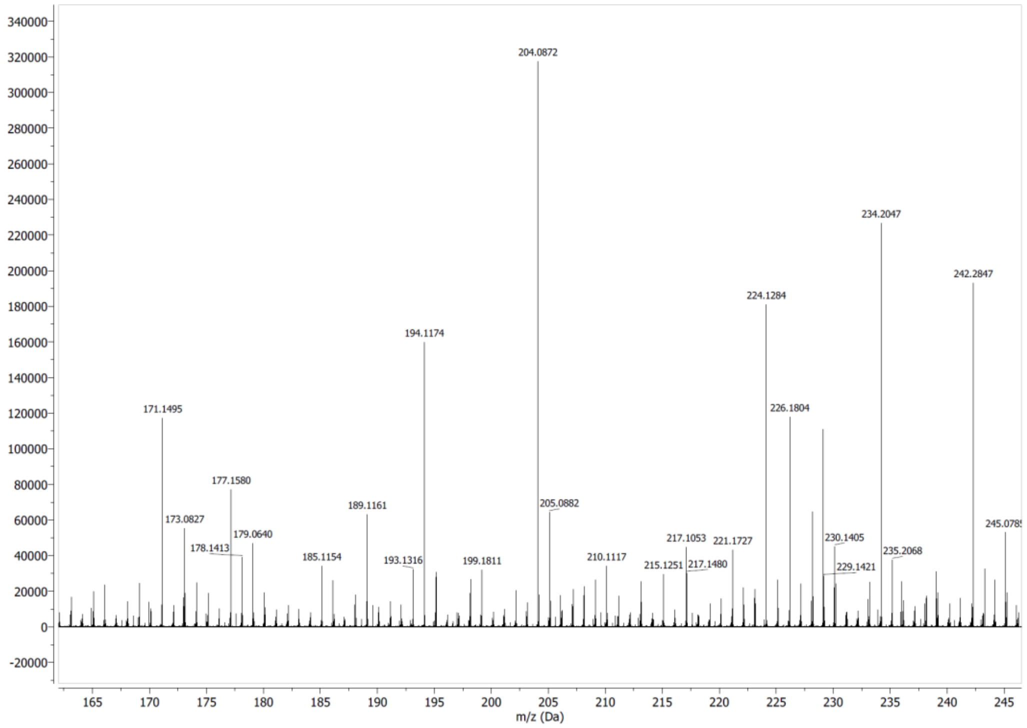

Figure S42. Partial HRESIMS spectrum of compound **8**.

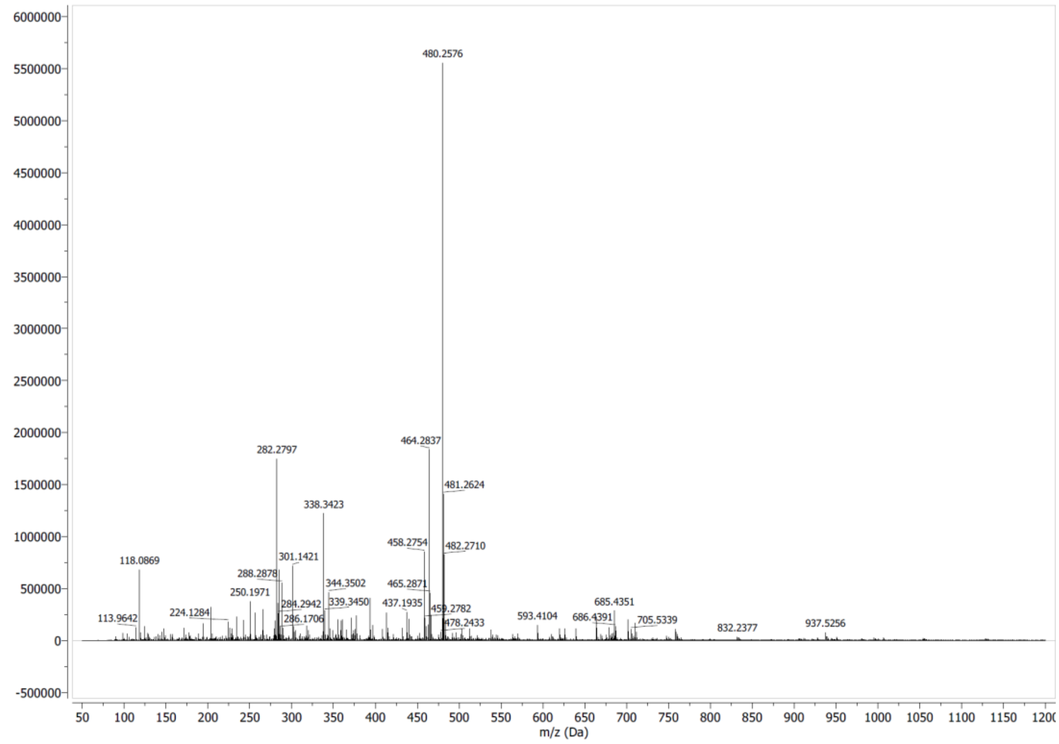

Figure S43. HRESIMS spectrum of compound **8**.

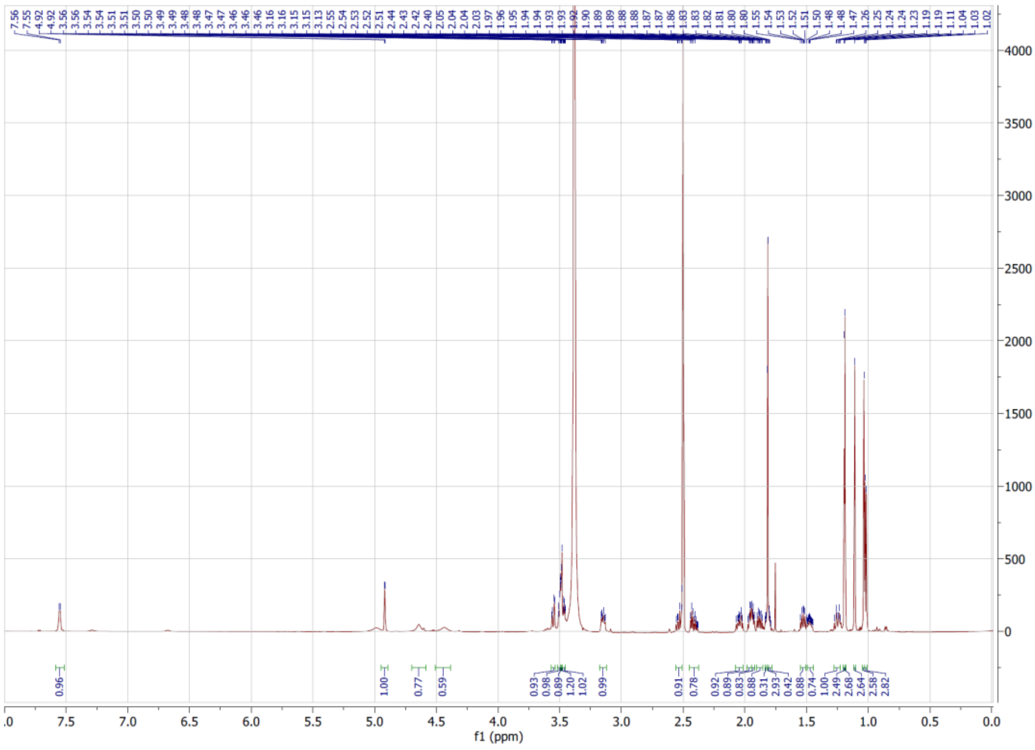

Figure S44. ^{1}H NMR (600 MHz, DMSO-d_6) spectrum of compound **9**.

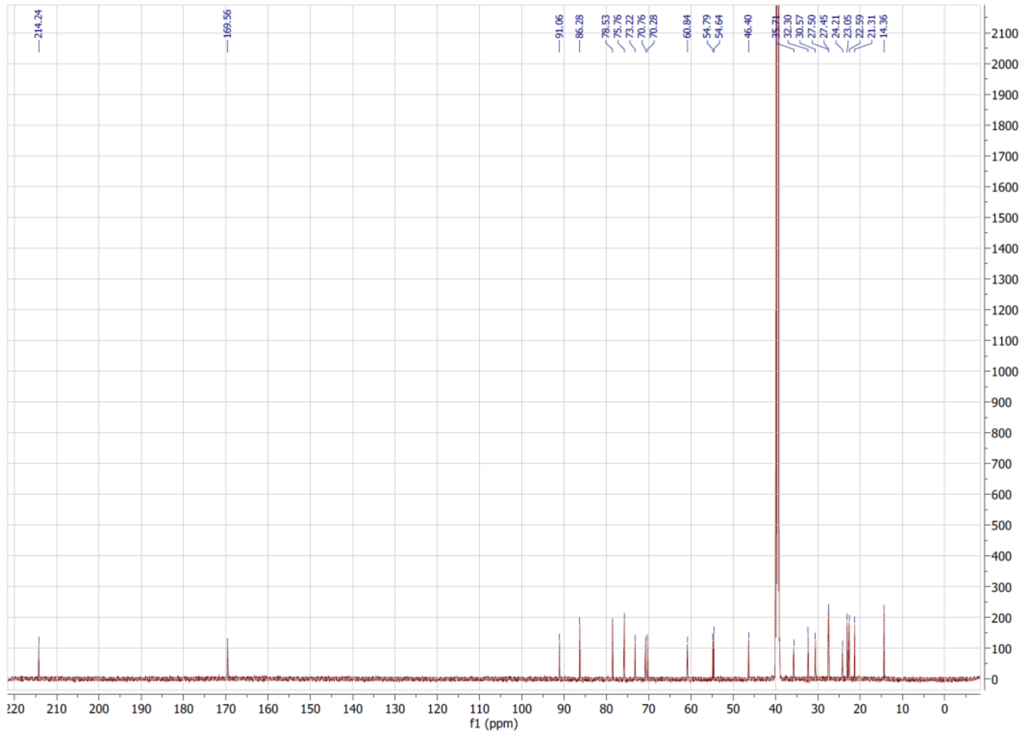

Figure S45. ^{13}C NMR (150 MHz, DMSO-d_6) spectrum of compound **9**.

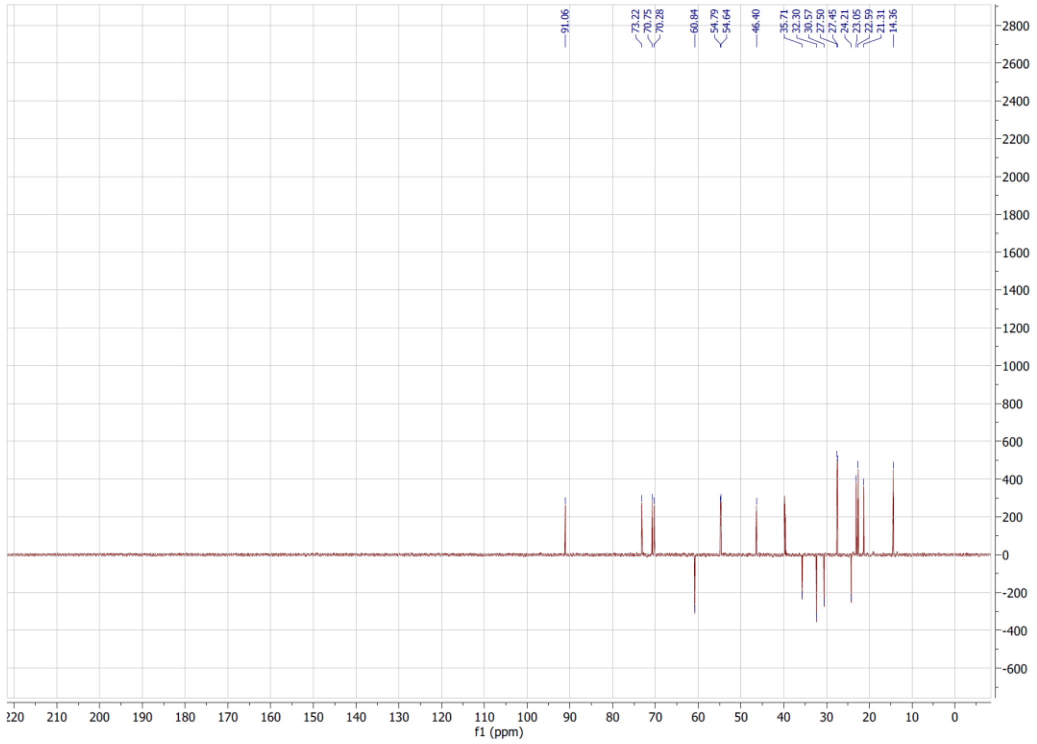

Figure S46. DEPT (150 MHz, DMSO-d_6) spectrum of compound **9**.

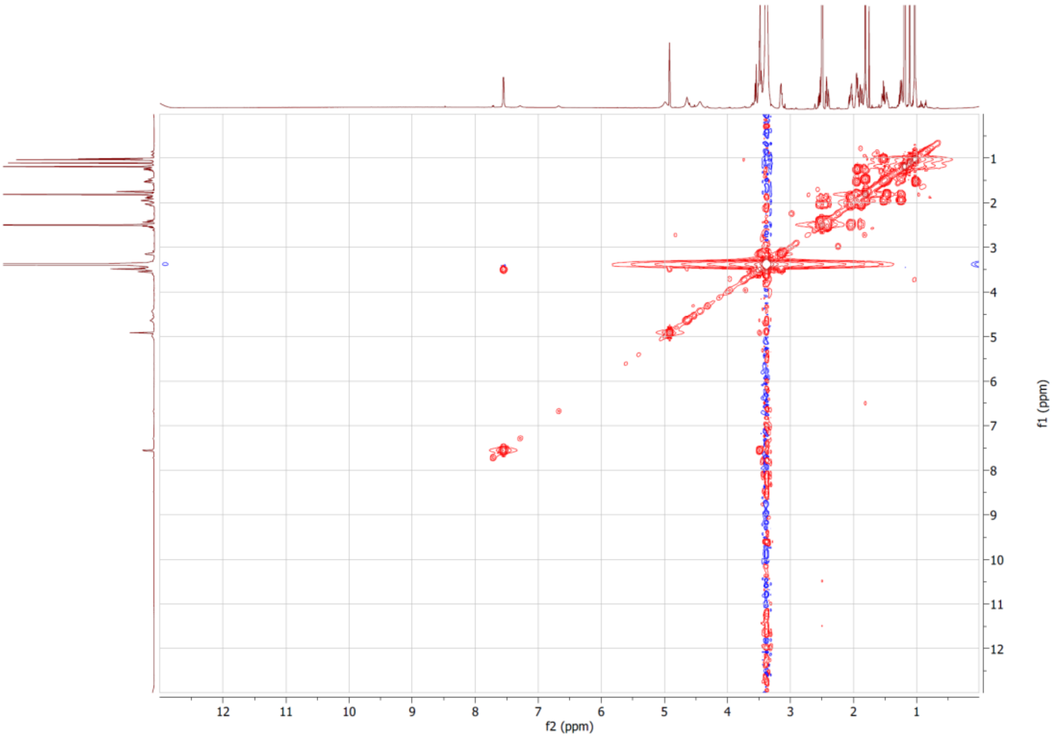

Figure S47. ^{1}H-^{1}H COSY (DMSO-d_6) spectrum of compound **9**.

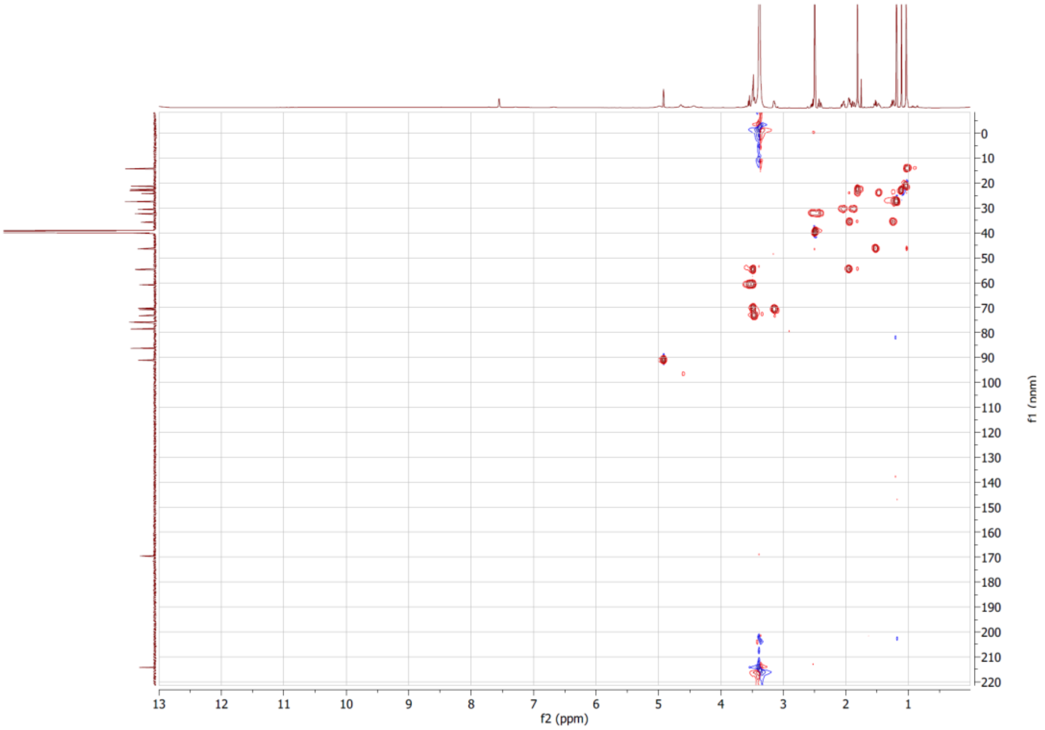

Figure S48. HSQC (DMSO-d_6) spectrum of compound **9**.

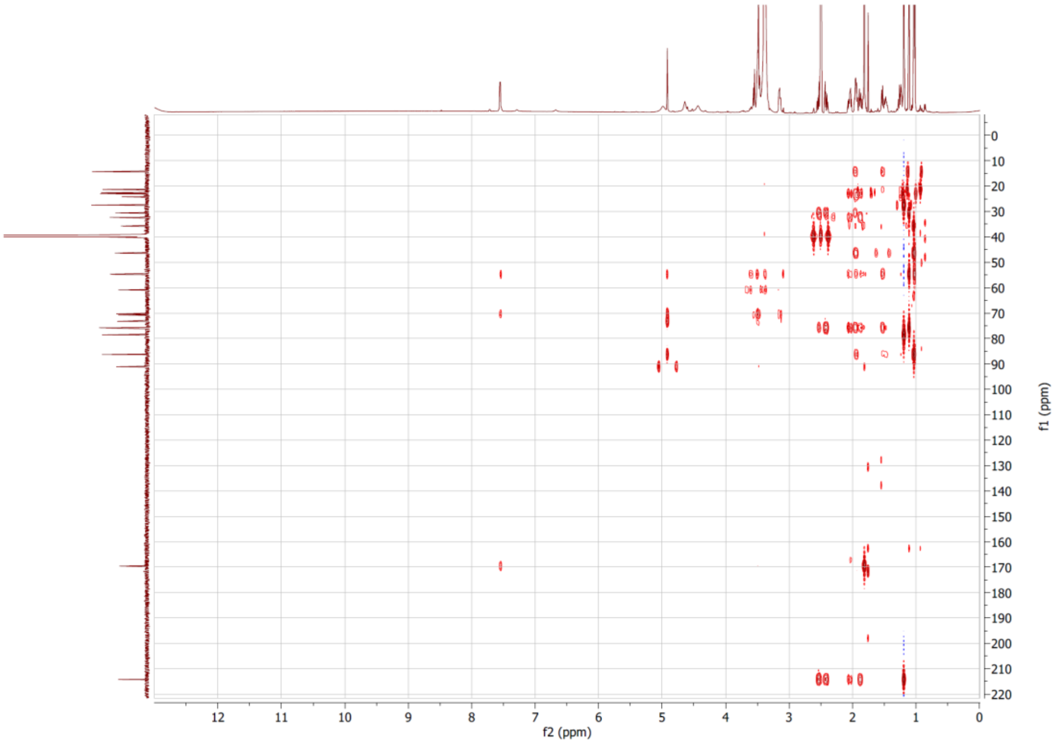

Figure S49. HMBC (DMSO-d_6) spectrum of compound **9**.

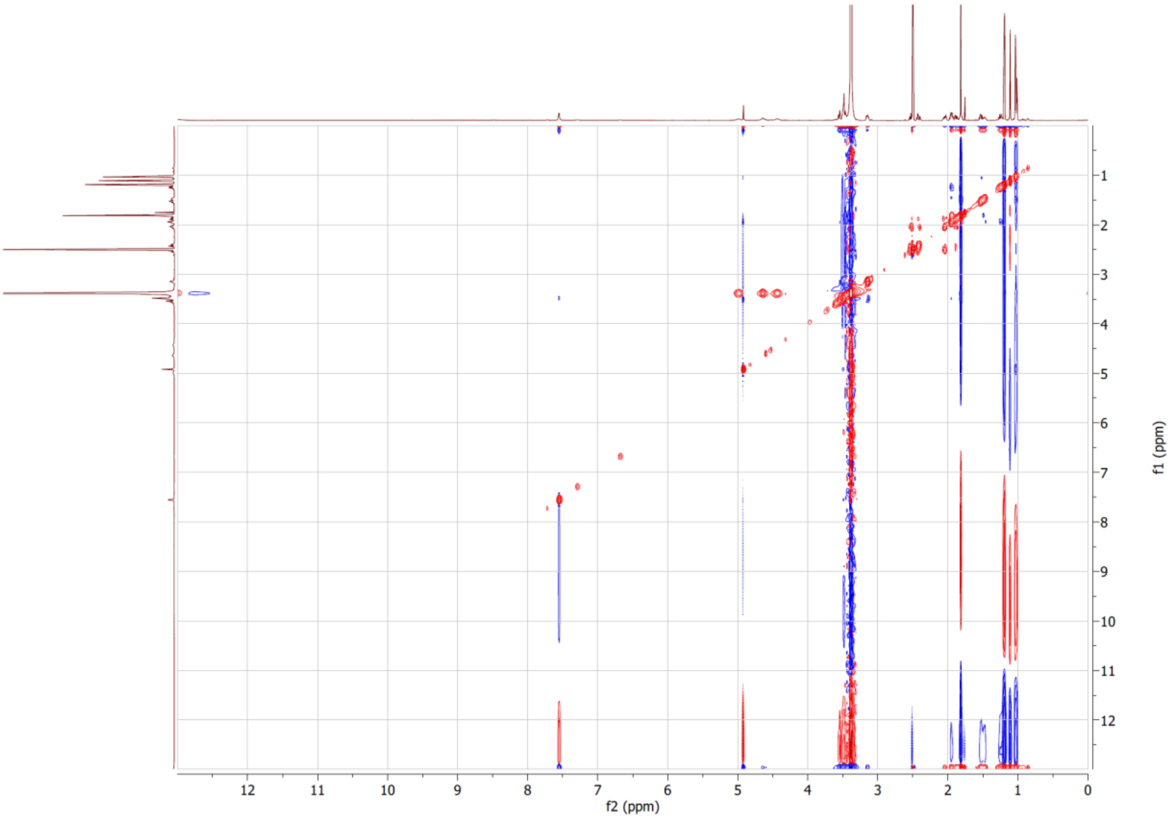

Figure S50. ROESY (DMSO-d_6) spectrum of compound **9**.

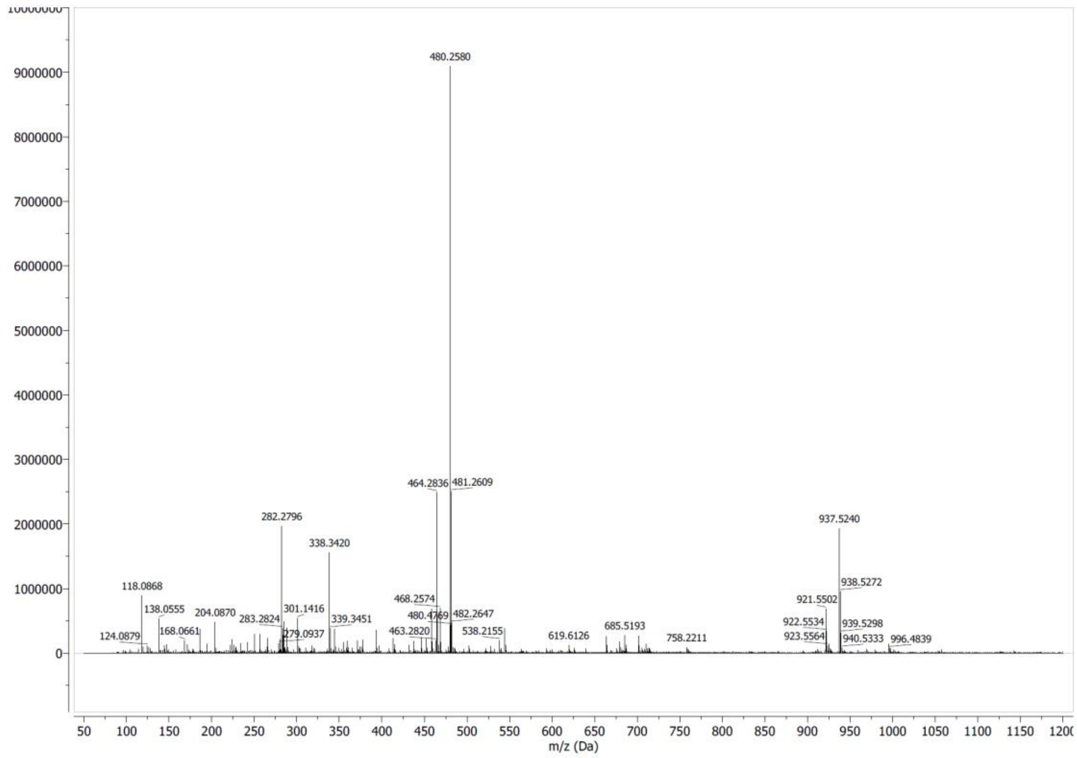

Figure S51. HRESIMS spectrum of compound **9**.

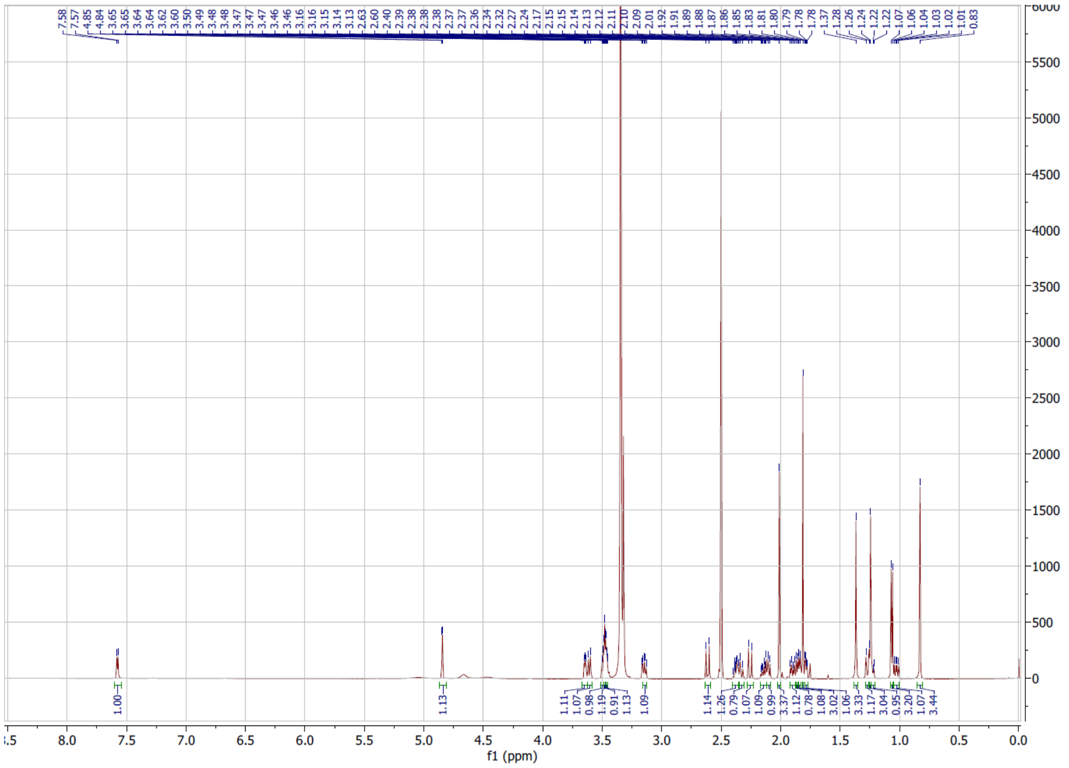

Figure S52. ^{1}H NMR (600 MHz, DMSO-d_6) spectrum of compound **10**.

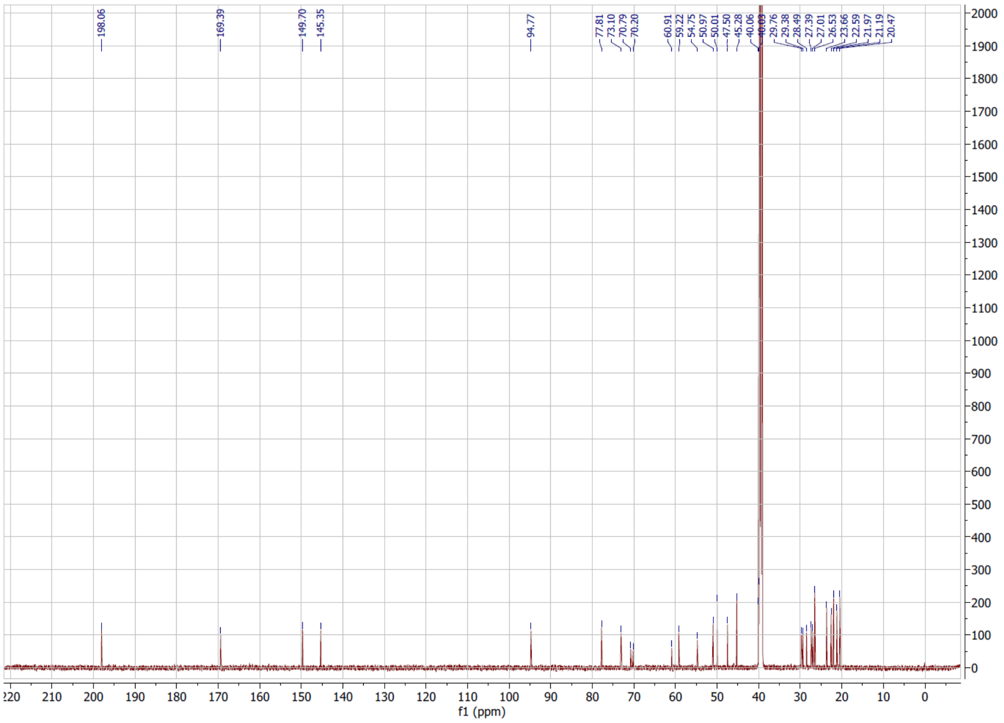

Figure S53. ^{13}C NMR (150 MHz, DMSO-d_6) spectrum of compound **10**.

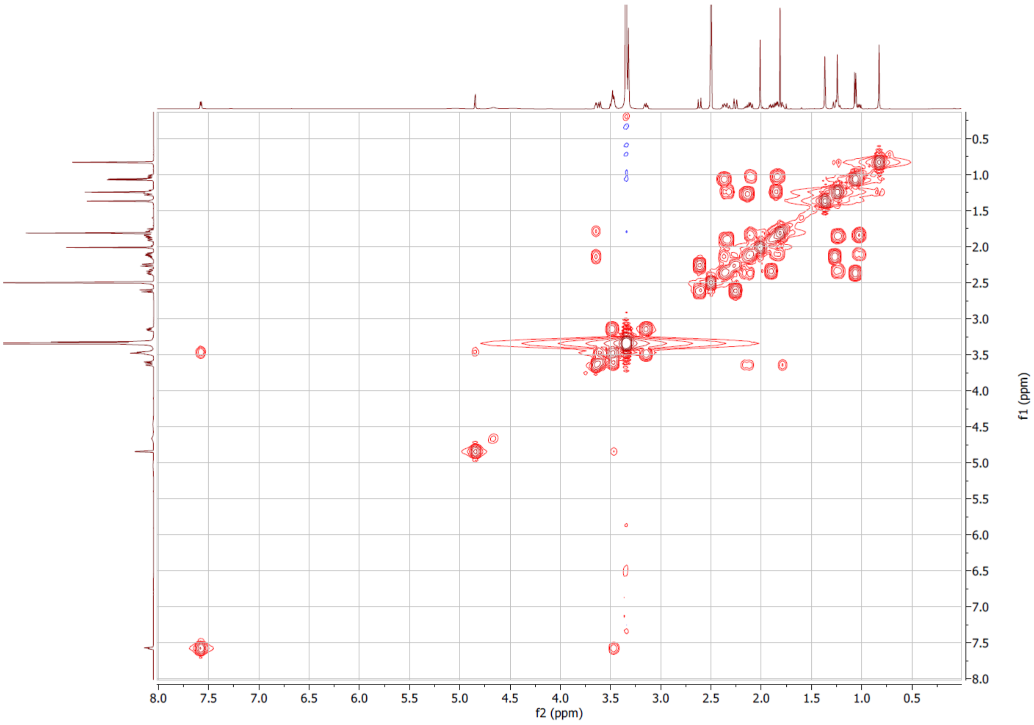

Figure S54. ^{1}H-^{1}H COSY (DMSO-d_6) spectrum of compound **10**.

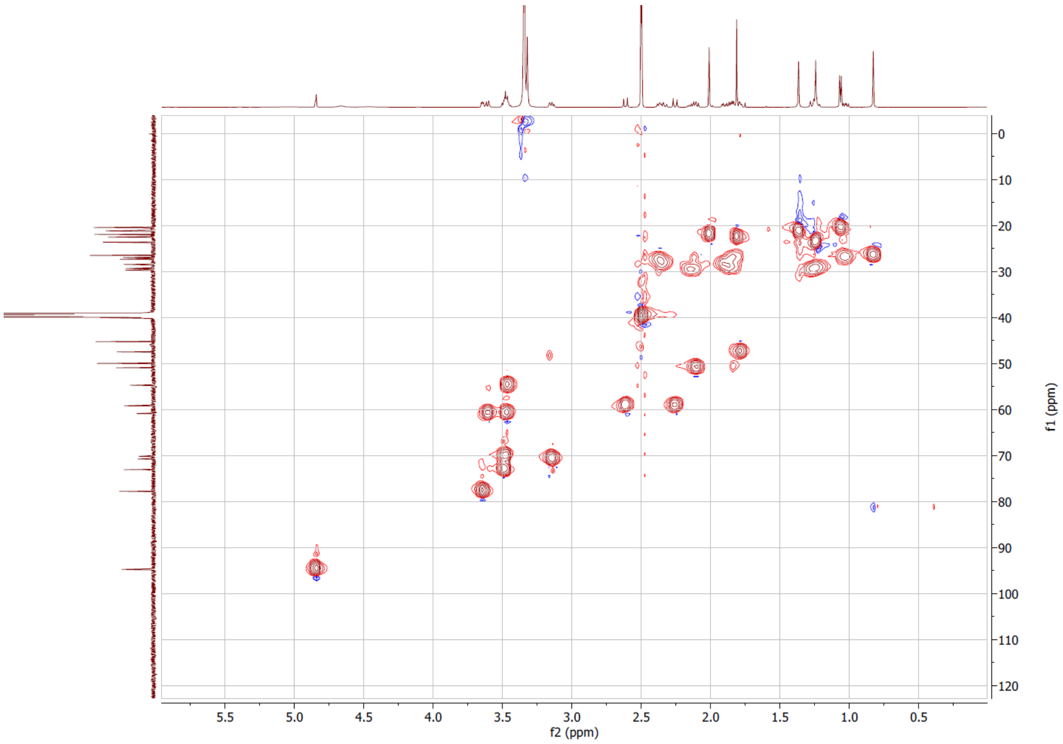

Figure S55. HSQC (DMSO-d_6) spectrum of compound **10**.

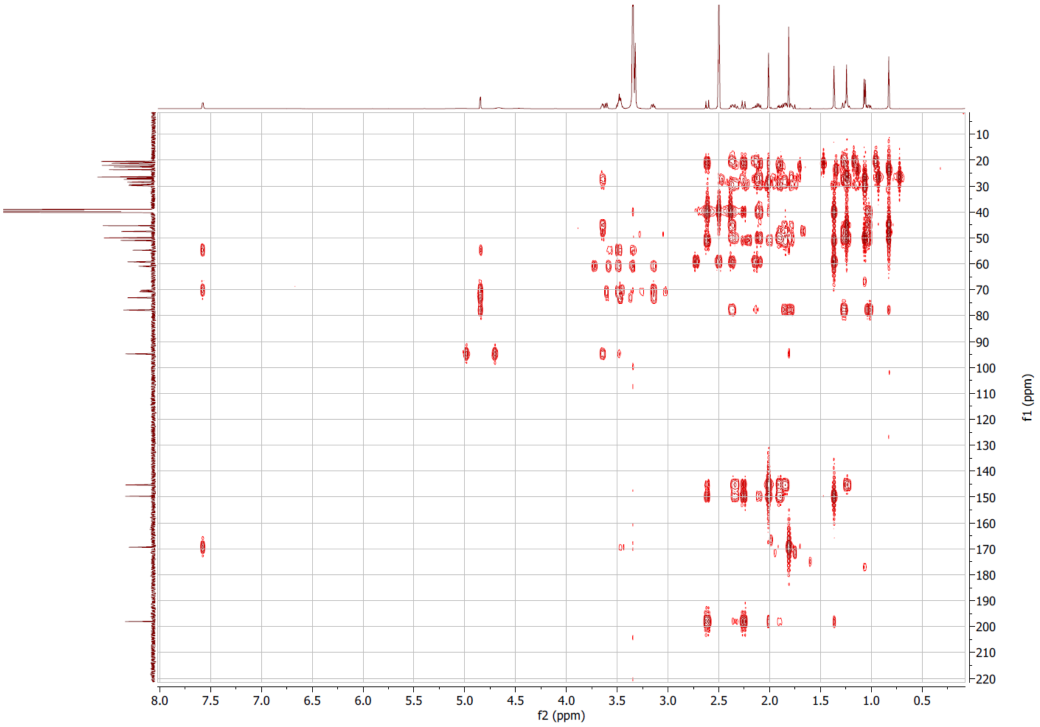

Figure S56. HMBC (DMSO-d_6) spectrum of compound **10**.

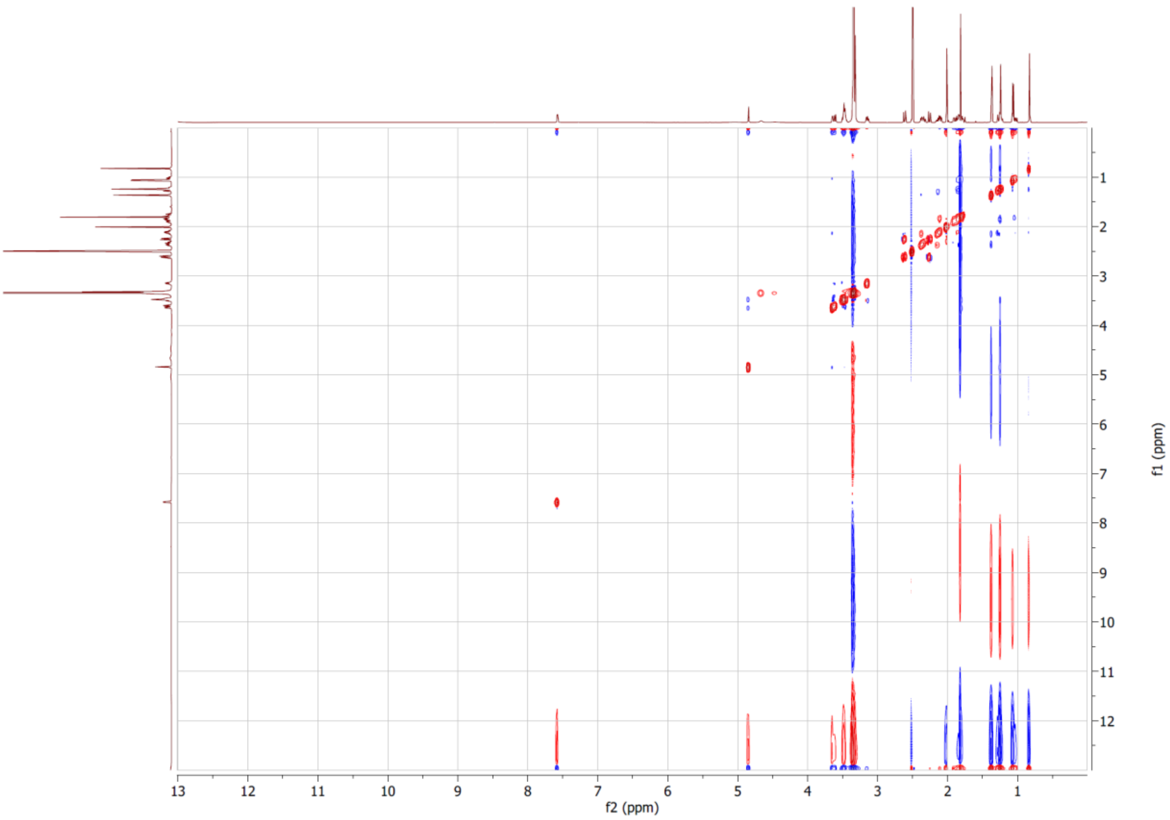

Figure S57. ROESY (DMSO-d_6) spectrum of compound **10**.

Mass Spectrum SmartFormula Report

Analysis Info

Analysis Name D:\Data\MS\data\202106\wangliyan_34A-9_pos_3_01_10959.d
Method LC_Direct Infusion_pos_70-500mz.m
Sample Name wangliyan_34A-9_pos
Comment

Acquisition Date 6/21/2021 10:17:51 AM
Operator SCSIO
Instrument maXis 255552.00029

Acquisition Parameter

Source Type	ESI	Ion Polarity	Positive	Set Nebulizer	0.4 Bar
Focus	Active	Set Capillary	4500 V	Set Dry Heater	180 °C
Scan Begin	70 m/z	Set End Plate Offset	-500 V	Set Dry Gas	4.0 l/min
Scan End	1500 m/z	Set Charging Voltage	0 V	Set Divert Valve	Waste
		Set Corona	0 nA	Set APCI Heater	0 °C

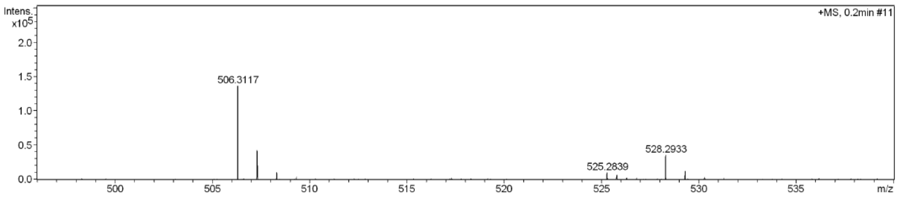

Meas. m/z	#	Ion Formula	Score	m/z	err [ppm]	err [mDa]	mSigma	rdb	e⁻ Conf	N-Rule
506.3117	1	C28H44NO7	100.00	506.3112	0.9	0.5	6.7	7.5	even	ok
528.2933	1	C28H43NNaO7	100.00	528.2932	-0.3	-0.2	16.9	7.5	even	ok
1011.6161	1	C56H87N2O14	100.00	1011.6152	0.9	0.9	16.1	14.5	even	ok
1033.5993	1	C56H86N2NaO14	100.00	1033.5971	2.1	2.1	37.0	14.5	even	ok

wangliyan_34A-9_pos_3_01_10959.d
Bruker Compass DataAnalysis 4.1 printed: 6/21/2021 10:22:28 AM by: SCSIO Page 1 of 1

Figure S58. HRESIMS spectrum of compound **10**.

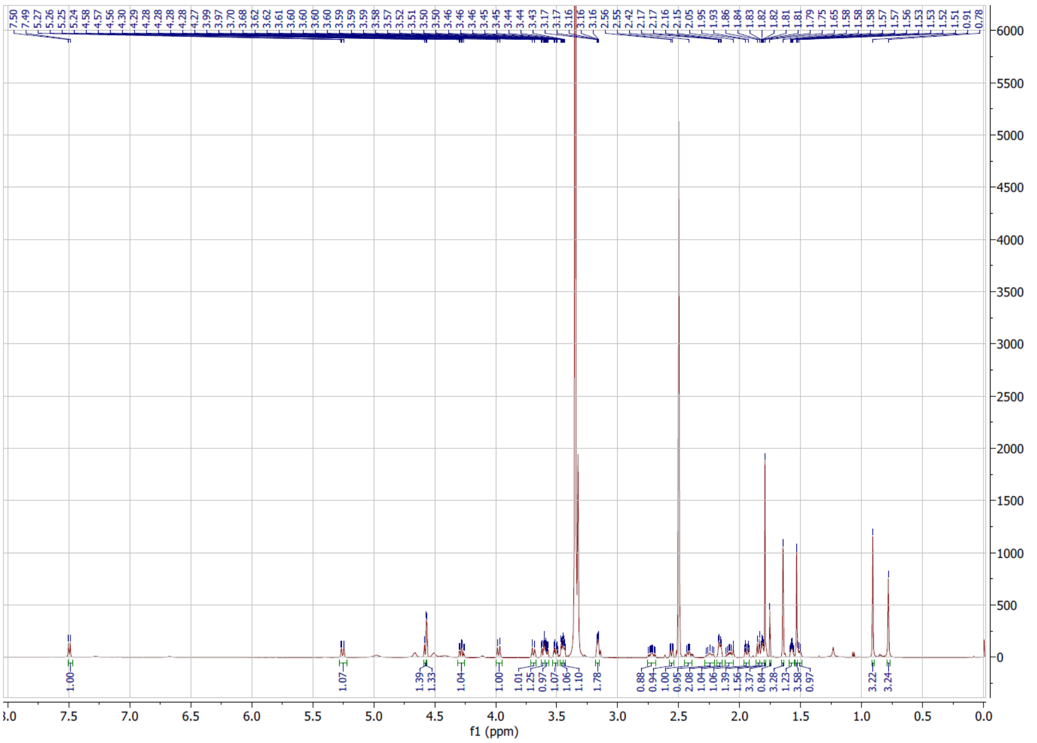

Figure S59. ^{1}H NMR (600 MHz, DMSO-d_6) spectrum of compound **11**.

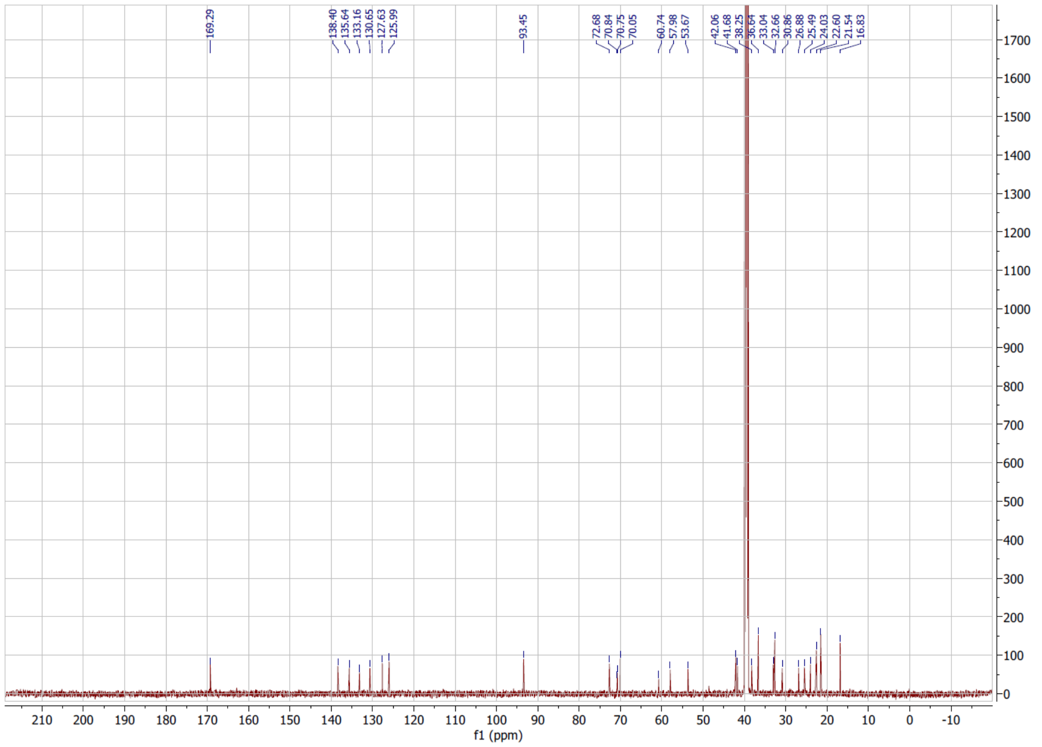

Figure S60. ^{13}C NMR (150 MHz, DMSO-d_6) spectrum of compound **11**.

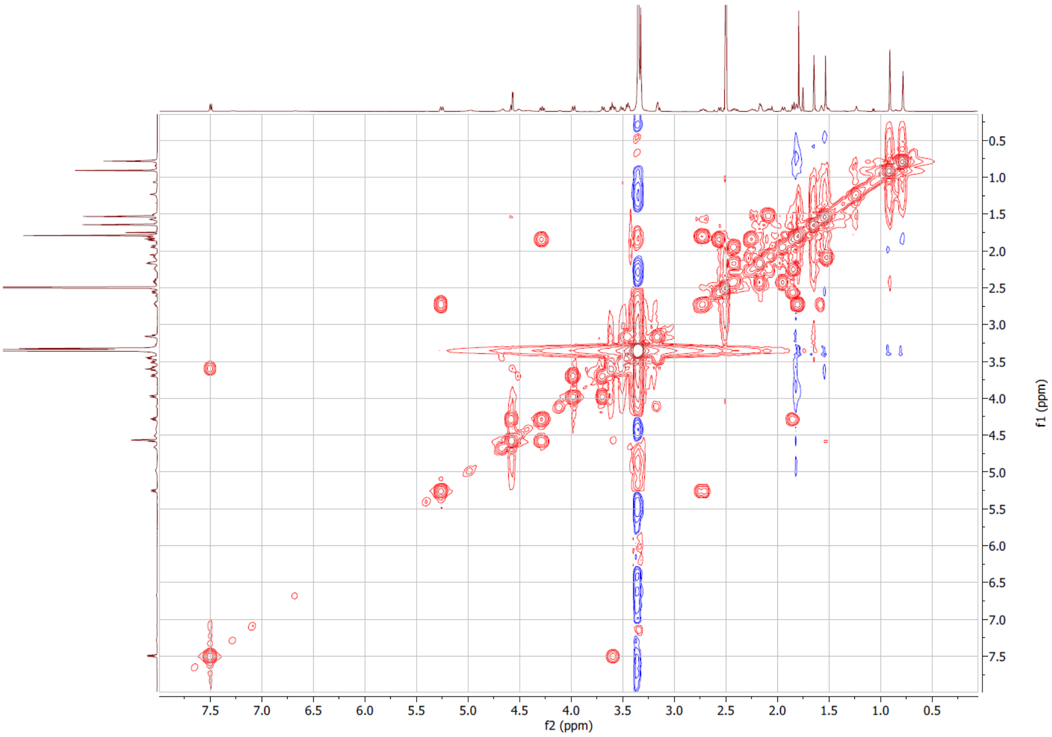

Figure S61. ^{1}H-^{1}H COSY (DMSO-d_6) spectrum of compound **11**.

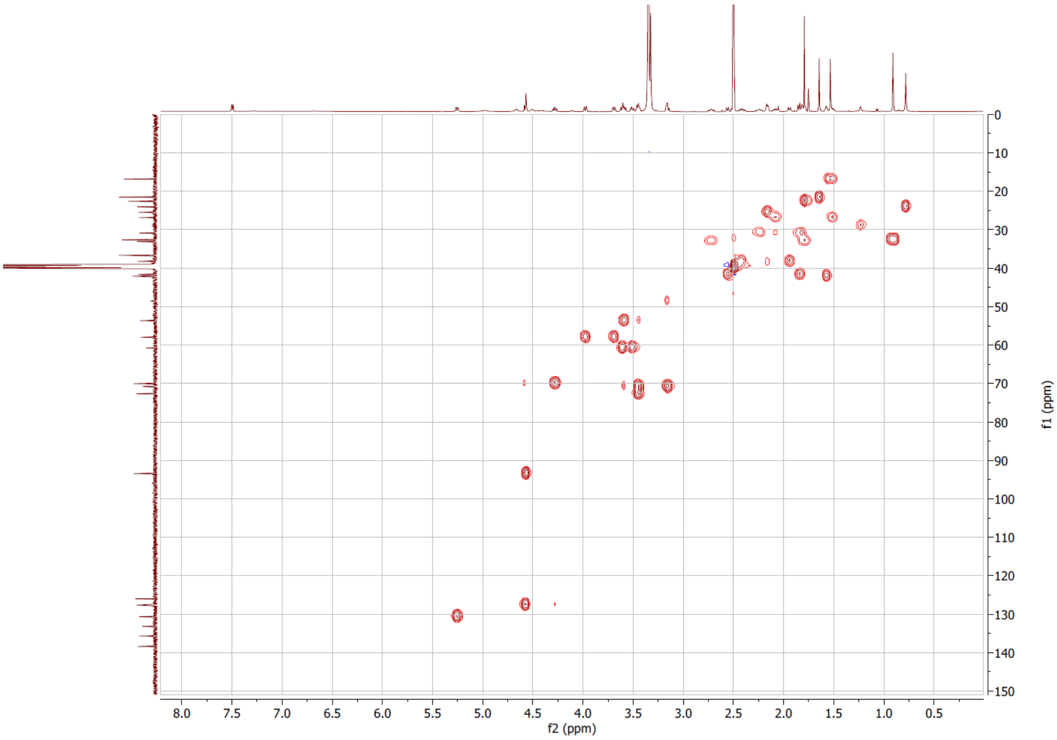

Figure S62. HSQC (DMSO-d_6) spectrum of compound **11**.

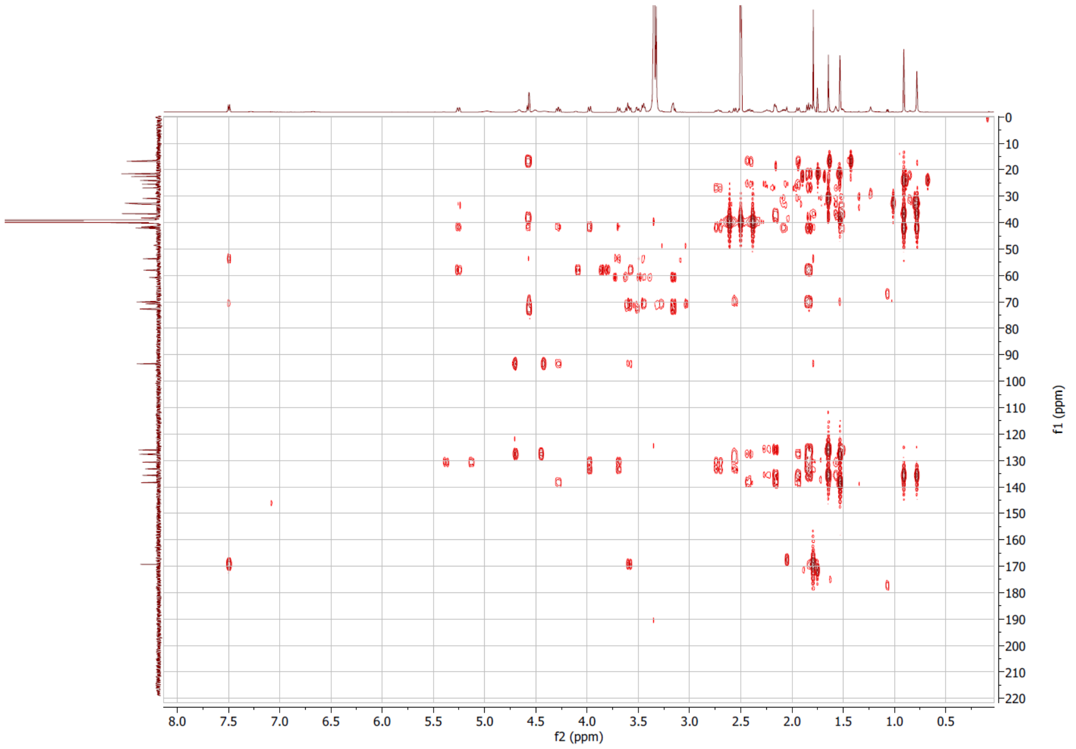

Figure S63. HMBC (DMSO-d_6) spectrum of compound **11**.

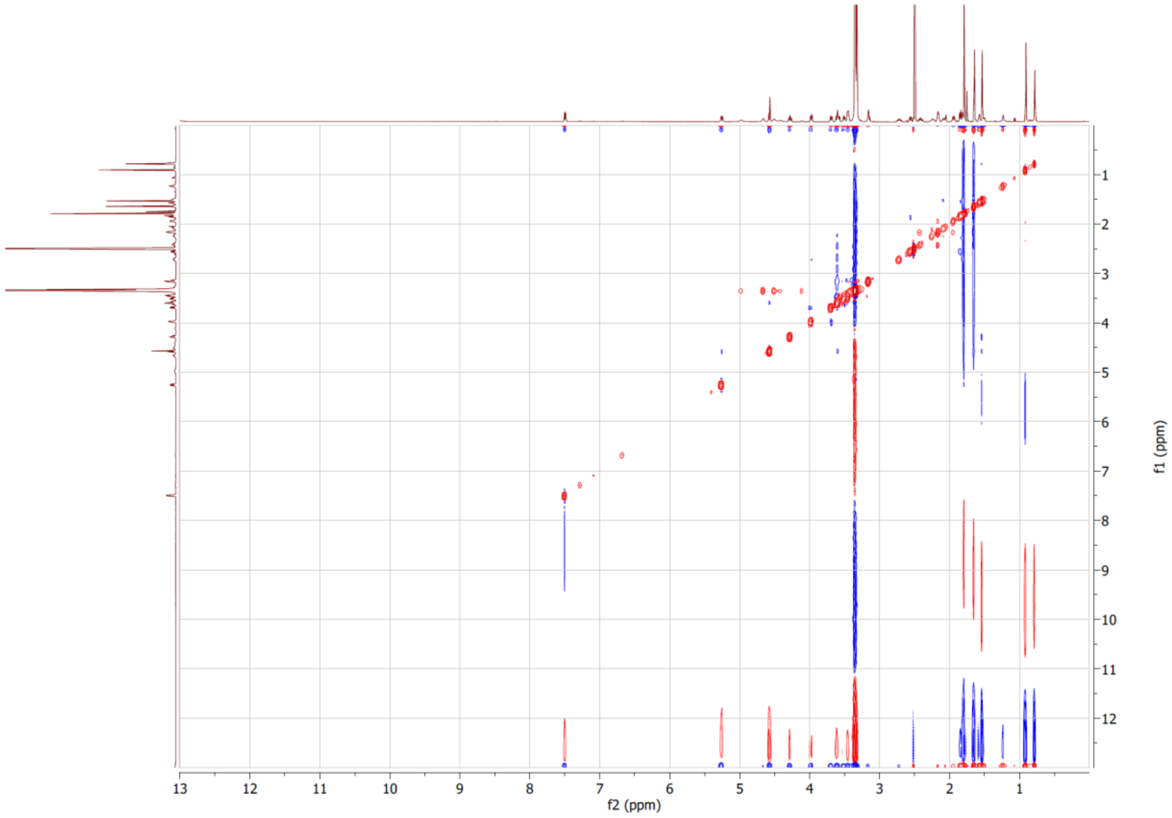

Figure S64. ROESY (DMSO-d_6) spectrum of compound **11**.

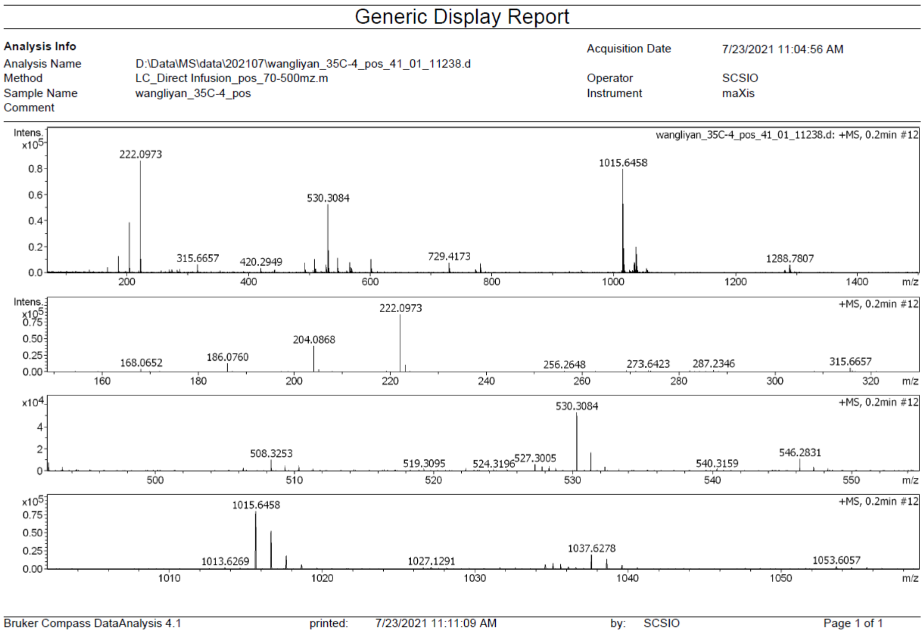

Figure S65. HRESIMS spectrum of compound **11**.
